# Modular Synthesis and Antiproliferative Activity of New Dihydro-1*H*-pyrazolo[1,3-*b*]pyridine Embelin Derivatives

**DOI:** 10.3390/ph14101026

**Published:** 2021-10-08

**Authors:** Pedro Martín-Acosta, Ángel Amesty, Miguel Guerra-Rodríguez, Borja Guerra, Leandro Fernández-Pérez, Ana Estévez-Braun

**Affiliations:** 1 Departamento de Química Orgánica, Instituto Universitario de Bio-Orgánica Antonio González, Universidad de La Laguna, Avda. Astrofísico Francisco Sánchez No. 2, 38206 Tenerife, Spain; pmartina@ull.edu.es (P.M.-A.); aarnesty@ull.edu.es (Á.A.); 2 Instituto Universitario de Investigaciones Biomédicas y Sanitarias (IUIBS), Farmacología Molecular y Traslacional (BIOPharm), Universidad de Las Palmas de Gran Canaria (ULPGC), 35001 Las Palmas de Gran Canaria, Spain; miguel.guerra106@alu.ulpgc.es (M.G.-R.); borja.guerra@ulpgc.es (B.G.)

**Keywords:** multicomponent reaction, embelin, antiproliferative activity, SAR

## Abstract

A set of new dihydro-1*H*-pyrazolo[1,3-*b*]pyridine and pyrazolo[1,3-*b*]pyridine embelin derivatives was synthesized through a multicomponent reaction from natural embelin, 3-substituted-5-aminopyrazoles and aldehydes. The synthesized compounds were evaluated against three hematologic tumor cell lines, HEL (acute erythroid leukemia), K-562 (chronic myeloid leukemia) and HL-60 (acute myeloid leukemia), and five breast cancer cell lines (SKBR3, MCF-7, MDA-MB-231, BT-549, HS-578T). The primate non-malignant kidney Vero cell line was used as the control of cytotoxicity. From the obtained results, some structure–activity relationships were outlined. Furthermore, in silico prediction of physicochemical properties and ADME parameters were determined for the derivatives with the best antiproliferative values.

## 1. Introduction

**Figure 1:**
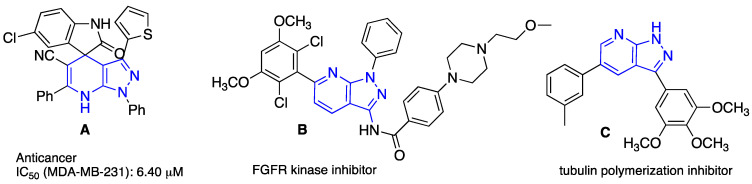
Antitumoral compounds with a dihydro-1*H*-pyrazolo[1,3-*b*]pyridine or a pyrazolo[1,3-*b*]pyridine moiety.

Heterocyclic compounds are of great importance in medicinal chemistry [1]. Molecules with these structures are present in many essential compounds for life as nucleic acids, amino acids, chlorophyll or vitamins, among others [2]. Titarenko et al. demonstrated during the development of the BioCore strategy [3] that more than 67% of the molecules included in the “Comprehensive Medicinal Chemistry” database contain heterocyclic rings. The non-aromatic heterocycles are twice as abundant as the heteroaromatics [4] and the combination of both is frequently present in alkaloids and drugs [5]. Among all heterocycles, nitrogenated heterocycles are particularly relevant in medicinal chemistry. Furthermore, an analysis of the approved drugs by the FDA database conducted by Njardarson et al. [6], revealed that 59% of them contained nitrogenated heterocycles. Pyrazoles are very attractive nitrogenated scaffolds since molecules having pyrazoles fused to other heterocycles such as dihydro-1*H*-pyrazolo[1,3-*b*]pyridines and pyrazolo[3,4-*b*]pyridines have raised great interest due to the biological activities that they exhibit. Figure 1 shows some examples of antitumoral compounds that exhibit this type of structure such as spirooxindoles (**A**) with cytotoxic activity against triple-negative breast cancer MDA-MB-231 cell line [7], compound (**B**) with potent and selective inhibitory activity against FGFR kinase [8] and compound (**C**) as potent tubulin polymerization inhibition [9]. 

Several synthetic methodologies for the preparation of dihydro-1*H*-pyrazolo[1,3-*b*] pyridines and pyrazolo[3,4-*b*]pyridines are found in the literature. Of special interest are those based on multicomponent reactions (MCRs), such as those involving the condensation of 1,3-dicarbonyl compounds with aldehydes and aminopyrazole derivatives [10]. Thus, for instance, some coumarin-fused pyridines/dihydropyridines were obtained from aminopyrazoles, 4-hydroxycoumarin and arylglyoxals [11,12]. Another example is the preparation of spiro[indoline-3,4′-pyrazolo[3,4-*b*]pyridines] from isatins, pyrazol-5-amines and β-ketonitriles [13].

Although the use of MCR allows us a quick and easy access to molecules with complex structural motifs, one of the problems associated with the use of this kind of methodology is related to the selectivity in the formation of the reaction products. Consequently, when a synthetic strategy involving a domino mechanism is designed, it is of significant importance to evaluate the different functionalities of the reagents used, the possible reaction products, as well as the different reaction parameters in order to control the selectivity of the transformation. An example of the former is the work published by Kappe et al. where the multicomponent condensation reaction of 3-aminopyrazoles, with 1,3-diketones and aromatic aldehydes, allows the obtaining of different products under different reaction conditions, due to the presence of three non-equivalent nucleophilic centers in the aminopyrazole component [14].

With these antecedents and because of our interest in the preparation of bioactive derivatives from the natural benzoquinone embelin [15,16,17,18], we decided to synthesize new dihydro-1*H*-pyrazolo[1,3-*b*]pyridine and pyrazolo[1,3-*b*]pyridine embelin analogues and evaluate their potential as antiproliferative agents against eight different human hematological (HEL, K-562, HL-60), and breast cancer cell lines (SKBr3, MCF-7, MDA-MB-231, BT-549 and HS-578).

## 2. Results and Discussion

Since embelin (**1**) has a masked 1,3-dicarbonyl moiety, the condensation of (**1**) with 4-nitrobenzaldehyde (**2a**) and 3-phenyl-5-aminopyrazole (**3a**) was carried out in order to obtain the desired dihydro-1*H*-pyrazolo[1,3-*b*]pyridine derivatives, using EtOH as solvent at room temperature. Ethylendiamine diacetate (EDDA, 10 mol %) was also used as an effective organocatalyst for the initial Knoevenagel condensation. Under these reaction conditions the product **4a** was obtained in 33% yield. The structure of **4a** was determined and corroborated by 1D and 2D NMR experiments (Supplementary Materials). 

Next, we proceeded to optimize the reaction conditions, focusing on achieving the highest yield to use small amounts of natural embelin due to the limited amount of it available in our laboratory. When the reaction was carried out under conventional heating using an equimolar ratio of the reagents (Table 1, entry 2), the product **4a** was obtained in 75% yield. Since heating was necessary in order to obtain a significant yield, we decided to test the use of MW irradiation to improve yields by shortening the reaction time and minimizing the side products. Additionally, we tried to use an alternative solvent, and modify the ratio of the reagents. In this sense, we selected DCE since in a previous work with embelin the use of DCE under MW irradiation produced very good results [19]. Considering this, it was found that the combination of DCE, and an excess of 0.5 equiv of aldehyde and 5-aminopyrazol afforded the corresponding adduct in 94% yield (entry 7).

Once the reaction conditions were optimized, we examined the scope of this three-component reaction using different substituted aromatic, heteroaromatic and aliphatic aldehydes. The structures and yields of the obtained products are shown in Table 2.

As we can observe, different yields were obtained depending on the type of aldehyde used in the Knoevenagel condensation. Thus, with aromatic aldehydes substituted with both electron-withdrawing (**4a**–**4k**) and electron-donating groups (**4l**–**4o**) good yields (73–98%) were obtained. The use of heteroaromatic aldehydes led to the corresponding products (**4p**–**4s**) in moderate yields (42–63%). This methodology is also tolerant to aliphatic aldehydes since the corresponding dihydro-1*H*-pyrazolo[1,3-*b*]pyridine adducts (**4t**–**4w**) were obtained.

**Scheme 1:**
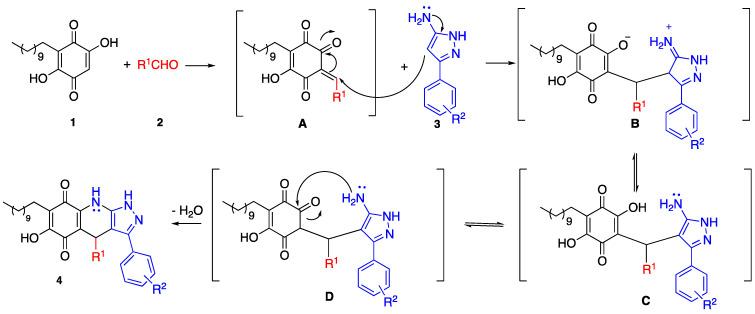
Plausible formation of dihydropyrazolopyridine embelin derivatives.

A plausible mechanism for the formation of these embelin derivatives (**4**) is shown in Scheme 1. Knoevenagel condensation between the aldehyde (**2**) and embelin (**1**) results in the formation of a methylene quinone reactive intermediate (**A**), which is trapped by the 3-phenyl-5-aminopyrazole (**3**). The reaction takes place via Michael addition to the methylene quinone system by the nucleophilic carbon in the α position to the amino group of the 3-phenyl-5-aminopyrazole to give the intermediate (**B**) which after a proton transfer gives the dihydropyridine system by intramolecular cyclization and dehydration.

These compounds were subsequently subjected to cellular phenotypic assays against eight different tumor cell lines. Three hematologic tumor cell lines, HEL (acute erythroid leukemia), K-562 (chronic myeloid leukemia) and HL-60 (acute myeloid leukemia), and five breast cancer cell lines (SKBR3, MCF-7, MDA-MB-231, BT-549, HS-578T). The primate non-malignant kidney Vero cell line was used as control of cytotoxicity. The results obtained are shown in Table 3.

None of the compounds evaluated showed significant cytotoxic activity in the triple negative breast cancer cell lines MDA-MB-231 and HS-578T. Several of the compounds showed good cytotoxic activity against the rest of cell lines evaluated with IC_50_ values between 0.7 and 7.5 μM.

In general terms, the best results were obtained in the leukemia cell lines compared to the breast cancer cell lines. Concerning the leukemia cell lines, compound **4a**, with a 4-NO_2_ group, exhibited the best cytotoxic activity in HL60 with an IC_50_ of 0.70 ± 0.14 μM. This compound also showed good activity values in HEL (1.05 ± 0.35 μM) and K562 (1.25 ± 0.35 μM). Derivatives **4c**, **4d**, **4e** and **4g**, with substituents 4-Cl, 4-Br, 4-F and 4-CF_3_, respectively, also presented good values of cytotoxic activity in the three leukemia cell lines with IC_50_ values between 0.90 and 3.30 μM. The best IC_50_ value in acute erythroid leukemia (HEL) was achieved with compound **4g** (1.00 ± 0.42 μM), whereas in chronic myeloid leukemia cell line (K-562), the derivative **4k** (4-CN-Ph) showed the best IC_50_ value (0.92 ± 0.32 μM).

According to these first results, the importance of substituents in *para* position of the phenyl ring at the 1,4-dihydropyridine ring, is highlighted. For the derivatives **4f**, and **4h**, having a -F and a -NO_2_ group, at the *meta* position a loss of cytotoxic activity was detected compared to the corresponding *para*-substituted derivatives (**4e** and **4a**). Interestingly, the replacement of these electron-withdrawing groups at the *para* position of the phenyl ring by others such as the -COOH (**4i**), or -CO_2_CH_3_ (**4j**) also leads to a loss of activity.

On the other hand, the presence of electron-donor groups at the phenyl ring such as -NMe_2_, -3F-4MeO, 3-4-MeO, 3,4-methylenedioxy (**4l**, **4m**, **4n** and **4o**) leads to worse values of IC_50_ in the leukemia cell lines, compared with those of **4b** (-Ph). A clear loss of activity when heteroaromatic rings were attached at the dihydropyridin nucleus (**4p**–**4s**) was also observed, while aliphatic substituents (**4u**, **4v**, **4w**) leads to higher values of IC_50_ with the exception of the derivative **4t**, with a cyclohexyl group, which keeps a good cytotoxic activity (1.30 ± 0.28 μM) against HEL.

With respect to the breast cancer cell lines (SKBR3, MCF7 and BT549), derivative **4a** (4-NO_2_) showed the best cytotoxic activity against SKBR3 (1.30 ± 0.28 μM). In the MCF7 cell line, only the derivatives **4a** (4-NO_2_) and **4u** (-CH_2_CH_3_) were active, with IC_50_ values of 3.25 ± 1.91 and 4.75 ± 1.20 μM, respectively. Finally, several of the derivatives evaluated in BT549 cell line (**4a**, **4c**, **4d**, **4e** and **4g**) presented IC_50_ values around 3.00 μM.

All previously synthesized nitrogenated embelin derivatives were also evaluated in the Vero cell line of primate kidney, to determine its cytotoxicity in a non-tumor cell line and to study its viability for carrying out future in vivo tests. Interestingly, inhibition of cell viability induced by several compounds were 8- to 20-fold lower in Vero cells compared to hematological (e.g., **4c**, **4d**, **4g**, **4t**) and breast cancer (e.g., **4c**, **4d**, **4g**) cell lines. Taking advantage of the versatility of this multicomponent reaction, and given the good results of cytotoxic activity obtained for some of the dihydro-1*H*-pyrazolo[3,4-*b*]pyridine derivatives previously synthesized, we decided to carry out modifications in the quinone and aminopyrazole components to evaluate the effect of these structural variations on the cytotoxic activity.

Several of the compounds evaluated exhibit good values of cytotoxic activity in the different cell lines studied. The derivative **4g** shows a better selectivity against hematological tumor cell lines, a good cytotoxic activity in erythroleukemia (HEL, 1.00 ± 0.42 μM) and the presence of fluorinated groups in biologically active molecules, such as the trifluoromethyl group, is of great interest [20] due to metabolic degradation resistance [21]. Furthermore, **4g** showed low cytotoxicity (IC_50_ > 25 µM) in normal Vero cell line with a calculated selectivity index higher than 25-fold.

Taking into account all data mentioned above, compound **4g** was selected to continue with the preparation of analogues with the -4-CF_3_Ph group retained.

**Scheme 2:**
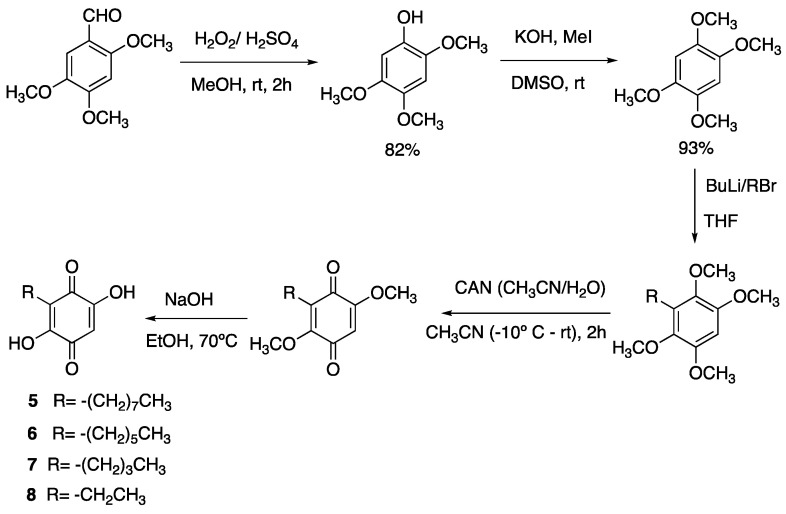
Preparation of benzoquinone analogues of embelin (**5**–**8**).

The influence of the side chain length on the cytotoxic activity was analyzed by the preparation of the benzoquinones **5** (R^2^ = (CH_2_)_7_CH_3_), **6** (R^2^ = (CH_2_)_5_CH_3_), **7** (R^2^ = (CH_2_)_3_CH_3_) and **8** (R^2^ = CH_2_CH_3_), which were synthesized following the methodology shown in Scheme 2 [22]. Table 4 shows the yields obtained in the preparation of derivatives **9**–**12**.

As we can observe in Table 5, the shortening of the side chain leads to a loss of activity, except in the SKBR3 cell line, where compound **10** (R^2^ = (CH_2_)_5_CH_3_) showed an increased antiproliferative activity (IC_50_ = 1.50 ± 1.27 μM) with respect to derivative **4g**. Derivatives **9**–**12** were also inactive against MDA-MB-231 and HS-578T cell lines.

**Scheme 3:**
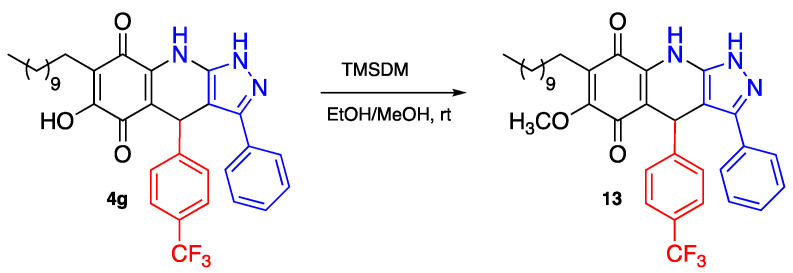
Synthesis of derivative **13** from **4g**.

The derivative **13** (Scheme 3) was also prepared using trimethyl silyl diazomethane in ether/methanol in order to evaluate the role of the free hydroxyl group on the cytotoxic activity. Compound **13** showed an IC_50_ value > 10 μM in all cell lines evaluated, which confirms the importance of the hydroxyl group for the cytotoxic activity.

Then, we focused on the component phenylaminopyrazole with the preparation of analogues having the 4-CF_3_-Ph moiety, the 11-carbon aliphatic chain (R^2^ = (CH_2_)_10_CH_3_) and the free hydroxyl group. Thus, different substituted phenylaminopyrazoles, 3-(furan-2-yl)-5-aminopyrazole and 3-methyl-5-aminopyrazole were used in the MCR.

**Scheme 4:**
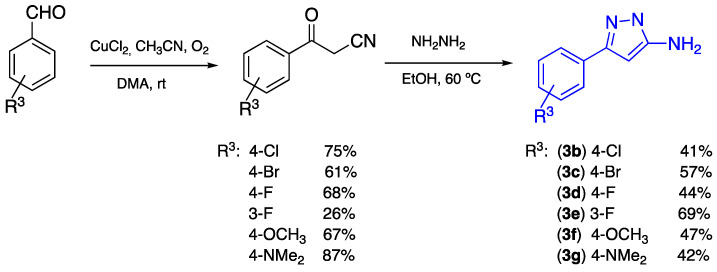
Synthesis of substituted 3-phenyl-5-aminopyrazoles (**3b**–**3g**) from aldehydes.

The different substituted 3-phenyl-5-aminopyrazoles were synthesized from the corresponding β-ketonitriles and hydrazine in EtOH at reflux [23]. On the other hand, the β-ketonitriles could be easily obtained by modifying the procedure described by Liu et al. [24], based on the copper catalyzed addition of acetonitrile to substituted benzylic alcohols, in the presence of oxygen. Thus, different substituted 3-phenyl-5-aminopyrazoles (**3b**–**3g**) were synthesized (Scheme 4).

The aminopyrazoles (**3b**–**3g**) as well as 3-methyl-5-aminopyrazole (**3h**) and 3-(furan-2-yl)-5-aminopyrazole (**3i**), were used for the preparation of the corresponding dihydro-1*H*-pyrazolo[3,4-*b*]pyridine derivatives through corresponding MCRs using embelin (**1**) and 4-(trifluoromethyl)benzaldehyde (**2g**). The results obtained are shown in Table 6.

The corresponding derivatives were obtained in good yields, from 76 to 94%, regardless of the nature of the substituent at the pyrazole ring (**14a**–**14f**). Furthermore, as we can see in Table 6, this methodology also allows us to use aminopyrazoles substituted with heteroaromatic groups such as derivative **14g** (82%) having a 2-furyl group, or aliphatic groups such as 5-methyl-3-aminopyrazole (**14h**, 78%).

The modified derivatives were evaluated against the eight tumor cell lines (Table 7).

Some of the new derivatives showed good cytotoxic activities, especially in breast cancer cell lines. The derivative **14c**, with a 4-F-Ph moiety at the pyrazole ring, showed the best cytotoxic activity with an IC_50_ of 0.59 ± 0.00 μM in MCF7, the derivatives **14e** with a 4-OMe substituent and **14g** with a 2-furyl group also presented an IC_50_ of 0.85 ± 0.03 μM and 0.93 ± 0.74 μM in the same cell line. Regarding the BT549 cell line, the presence of different substituents in the aromatic ring of the pyrazole seems to have a negative effect on the activity. For the SKBR3 cell line, several of the derivatives such as **14c** (4-F-Ph), **14d** (3-F-Ph), **14e** (4-MeO-Ph) and **14g** (2-furyl) presented a significant improvement in their cytotoxic activity with values of IC_50_ from 2.01 ± 1.12 µM to 2.40 ± 0.28 µM. All of them also showed an IC_50_ > 10 µM in MDA-MB-231 and HS-578BT cell lines.

Concerning the hematological tumor cell lines, we can observe an improvement in the cytotoxic activity for the derivative **14d** (3-F-Ph) with an IC_50_ of 1.05 ± 0.64 μM in HL60 cell line and 0.95 ± 0.4 μM in K562 cell line. Derivatives **14e** (4-MeO-Ph) and **14g** (2-furyl), as in some of the breast cancer cell lines, also have good cytotoxicity values in HL60 with IC_50_ of 1.10 ± 0.14 and 1.10 ± 0.04 μM, respectively.

**Scheme 5:**
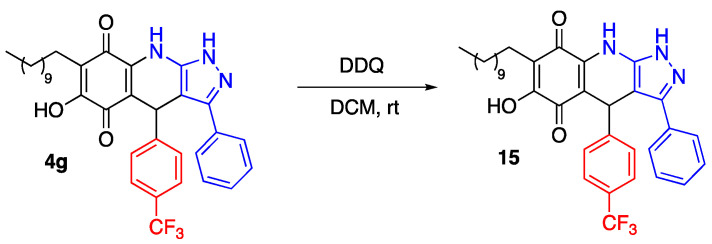
Synthesis of derivative **15** from **4g**.

Next, we decided to evaluate the effect of the planarity of this type of structure on cytotoxic activity. Thus, when compound **4g** was treated with DDQ, the corresponding pyrazolopyridin derivative (**15**) was obtained in 82% yield (Scheme 5), and it presented IC_50_ values higher than 10 μM in all cell lines studied.

**Figure 2:**
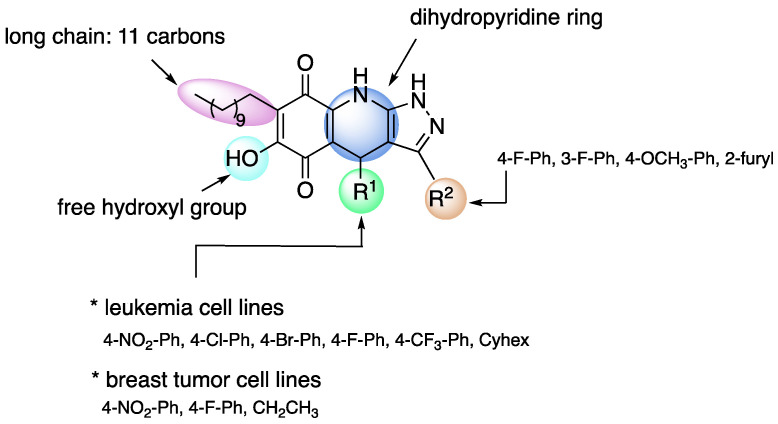
Structure–activity relationships.

Taking into account all mentioned results, Figure 2 displays a summary of the established structure–activity relationships (SARs) for these new antiproliferative dihydro-1*H*-pyrazolo[1,3-*b*]pyridine embelin derivatives.

**Figure 3:**
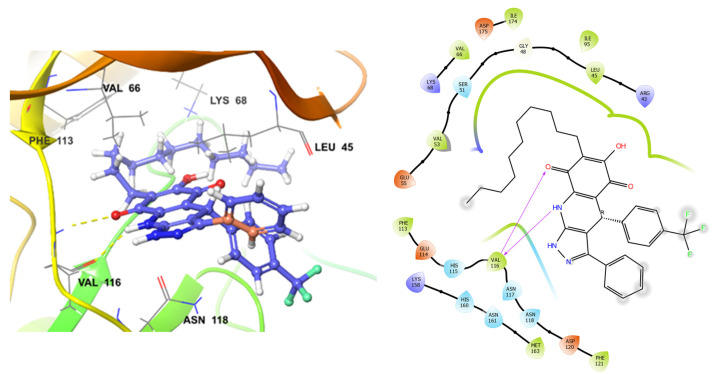
3D (**Left**) and 2D (**Right**) representation of the binding mode of **4g** with CK2 (PDB 3PE1).

Since other embelin derivatives have shown inhibitory activity against the human protein kinase CK2 [17,18], we think that CK2 could be also the target of this type of compounds. In this sense, docking studies were carried out with the most active compounds using Glide software [25] on the reported crystal structure of human protein kinase CK2 alpha subunit in complex with the inhibitor CX-4945 (PDB 3PE1). An analysis of the docking results showed that the compounds fit well and, as shown in Figure 3, the active site is fully occupied by the compound **4g**, the aliphatic chain was located at the bottom of the pocket. In this case, the alkyl chain established hydrophobic interactions with the residues Phe 113, Ile 95, Val 66 and Lys 68. Furthermore, two hydrogen bond interactions in the hinge region were observed between the residue Val 116 and the NH of the dihydropyridine ring and one of the quinonic carbonyl which explains the good value of docking score obtained during the simulation (−8.430 Kcal/mol).

Understanding the physicochemical properties and the pharmacokinetic profile (absorption, distribution, metabolism and excretion) of molecules is an essential step to avoid failures in the selection of lead molecules during the different stages of development and discovery of new drugs. Therefore, it is essential to design chemical leads with acceptable ADME and good drug-like properties. However, about the drug-likeness of natural product derivatives, it is important to mention that many derivatives of them do not comply with drug-like rules during the lead optimization process. A lot of these compounds are found outside the drug-like space because they usually have a higher molecular weight and can be more complex than drug-like. Although the greatest value of the use of the privileged structures present in natural products is that they were optimized during the course of evolution and also, they can explore parts of chemical space that synthetic drug-like compounds do not essentially cover.

In this sense in silico prediction of physicochemical properties, ADME parameters, Lipinski’s rule of five (Ro5), and Jorgensen’s rule of three and drug-likeness were determined for dihydro-1*H*-pyrazolo[1,3-*b*]pyridine embelin derivatives with the best antiproliferative values (**4a**, **4c**, **4e**, **4g**, **14c**, **14d**, **14e** and **14g**). This study was performed using the Qikprop module of Schrödinger software [25], which predicts physically and pharmaceutically relevant properties of organic molecules on the full 3D molecular structure and provides range to compare with the molecular properties of known 95% drugs. The predicted parameters and their recommended values are presented in Table 8. The rule of five [26] is a widely used filter to indicate if a compound presents good oral bioavailability. However, other parameters such as the total number of rotatable bonds and the polar surface area (PSA) are found to be important predictors of good oral absorption and bioavailability, independent of molecular weight [27].

As expected, the results presented in Table 8 show that the selected compounds do not meet all the properties required by the rules, such as having a MW < 500, log P < 5, thus showing two violations for Lipinski’s rule and a violation of the rule of three is established because the values of log S are <−5.7. Therefore, according the rules of three and five, embelin derivatives have higher lipophilicity and lower solubility in water, this result is reasonable due to the presence of the 11-carbon aliphatic chain of the embelin moiety. However, for an orally active compound, two violations of Lipinski’s rule and one violation of the rule of three are acceptable. Thus, these compounds show their properties having the maximum number of infractions of these rules.

Most of the molecules showed excellent predicted blood/brain partition co-efficient (QPlogBB) values which is a measure of the ability of a drug to cross the blood-brain barrier and also the blood-brain barrier mimic MDCK cell permeability (QPPMDCK) show satisfactory predictions for all the compounds. The calculated theoretical PSA values for all compounds allow to support the low ability of the compounds to penetrate the blood-brain barrier with the exception of compound **4a**. The most widely recommended PSA cutoff values are about 120–140 Å. all molecules showed excellent predicted Caco-2 cell (model for the gut-blood barrier) permeability.

On the other hand, the prediction of oral drug absorption (percent human oral absorption) was highly satisfactory for all the compounds, moreover human serum albumin binding co-efficient (QPlogKhsa) were found to be within an acceptable range which indicates their strong binding with plasma protein with the exception of compounds **4g**, **14d** and **14e**. Human serum albumin binding co-efficient is one of the key factors since it is related to the transport of many of the drugs to its targets once they enter into the circulatory system.

The presence of reactive functional groups (#rtvFG) is an important predicted additional property that alerts us to the presence of different groups such as silicon, aluminum, diazo, azide, carbonate, anhydride, etc., which is related to decomposition, reactivity and toxicity in vivo and also the presence of these groups can lead to false positives when the compounds are evaluated biologically while the predicted skin permeability (logKp) is within the recommended values.

Finally, the ADME profile of our compounds showed good values, mainly in those properties related to well calculated permeability, low toxicity and good protein-plasma interaction. These predictions linked to the values of biological activity are promising and, therefore, deserve further investigation for further optimization of the synthesized compounds.

## 3. Materials and Methods

### 3.1. General Experimental Procedures

IR spectra were obtained using a Fourier transform infrared spectrometer. NMR spectra were recorded in CDCl_3_ or DMSO-d_6_ at 500 or 600 MHz for ^1^H NMR and 125 or 150 MHz for ^13^C NMR. Chemical shifts are given in (δ) parts per million and coupling constants (*J*) in hertz (Hz). ^1^H and ^13^C spectra were referenced using the solvent signal as internal standard. Melting points were taken on a capillary melting point apparatus and are uncorrected. HREIMS were recorded using a high-resolution magnetic trisector (EBE) mass analyzer. Analytical thin-layer chromatography plates Polygram-Sil G/UV254 were used. Preparative thin-layer chromatography was carried out with Analtech silica gel GF plates (20 × 20 cm, 1000 Microns) using appropriate mixtures of ethyl acetate and hexanes. Microwave reactions were conducted in sealed glass vessels (capacity 5 mL) using a Biotage initiator microwave reactor. All solvents and reagents were purified by standard techniques reported [28] or used as supplied from commercial sources. All compounds were named using the ACD40 Name-Pro program, which is based on IUPAC rules. The embelin (**1**) used in the reactions was obtained from *Oxalis erythrorhiza* Gillies ex Hook and Arn following the procedure described in reference [29].

### 3.2. General Procedure for the Synthesis of Pyrazolo[3,4-b] Quinolin-5,8(4H,9H)-Dione Derivatives

To a MW tube equipped with a magnetic stir bar, embelin, 1.5 equiv of aldehyde, 1.5 equiv of 3-amino-5-phenylpyrazole and 10 mol % EDDA in 2 mL of DCE were added. The MW tube was sealed, and the reaction mixture was irradiated at 150 °C for 10 min. The products were isolated by filtration or purified by Shepadex LH-20.

### 3.3. 6-Hydroxy-4-(4-Nitrophenyl)-3-Phenyl-7-Undecyl-1H-Pyrazolo[3,4-b]Quinoline-5,8(4H,9H)-Dione (***4a***)

Following the general procedure described above, in a 5 mL MW tube, 30 mg of embelin (0.1 mmol), 23.4 mg of 4-nitrobenzaldehyde (0.15 mmol) and 24.4 mg of 3-amino-5-phenylpyrazole (0.15 mmol) were dissolved in 2 mL DCE and treated with 1.8 mg of EDDA (10 mol %). The tube was sealed, and the reaction mixture was irradiated at 150 °C for 10 min. Then, the reaction mixture was filtered and the obtained solid was washed with n-hex to yield 54.5 mg (94%) of **4a** as an amorphous violet solid. Mp: 234.0–234.7 °C; ^1^H-NMR (500 MHz, CDCl_3_) δ 0.86 (t, *J* = 7.1 Hz, 3H, H-11′’’), 1.22 (bs, 16H, H, H-3′’’-H-10′’’), 1.43 (m, 2H, H-2′’’), 2.41 (t, *J* = 7.6 Hz, 2H, H-1′’’), 5.64 (s, 1H, H-4), 7.31 (m, 2H, H-2′’ + H-6′’), 7.39 (m, 5H, H-2′-H-6′), 8.00 (d, *J* = 8.5 Hz, 2H, H-3′’ + H-5′’); ^13^C-NMR (125 MHz, CDCl_3_) δ 14.1 (CH_3_), 22.6 (CH_2_), 22.7 (CH_2_), 28.0 (CH_2_), 28.1 (CH_2_), 29.3 (CH_2_), 29.4 (CH_2_), 29.5 (CH_2_), 29.5 (CH_2_), 29.6 (CH_2_), 29.7 (CH_2_ X 2), 31.9 (CH_2_), 36.3 (CH), 102.9 (C), 106.6 (C), 116.6 (C), 123.6 (CH x 2), 124.0 (CH) 125.7 (CH), 126.7 (CH), 128.1 (C), 128.7 (C), 129.0 (CH X 2), 129.2 (CH x 2), 129.6 (C), 141.3 (C), 146.6 (C), 152.0 (C), 154.1 (C), 178.6 (C), 182.2 (C); EIMS *m*/*z* (%): 568 ([M^+^], 100), 446 (89), 427 (24), 306 (8); HREIMS *m*/*z* 568.2682 (calcd for C_33_H_36_N_4_O_5_ [M^+^] 568.2686); IR v_max_ 3430 (N-H), 3315 (O-H), 2922 (C-H aliph), 2851 (C-H aliph), 2322 (C-C arom), 1640 (C=O), 1586, 1518, 1482, (C=N, C=C), 1347 (N-N), 1313, 1272, 1237, 1187, 1139, 1119, 1009, 981, 864 cm^−1^.

### 3.4. 6-Hydroxy-3,4-Diphenyl-7-Undecyl-1H-Pyrazolo[3,4-b]Quinoline-5,8 (4H,9H)-Dione (***4b***)

Following the general procedure described above, in a 5 mL MW tube, 30 mg of embelin (0.1 mmol), 16.2 mg of benzaldehyde (0.15 mmol, 15.6 µL) and 24.4 mg of 3-amino-5-phenylpyrazole (0.15 mmol) were dissolved in 2 mL DCE and treated with 1.8 mg of EDDA (10 mol %). The tube was sealed, and the reaction mixture was irradiated at 150 °C for 10 min. Then, the reaction mixture was filtered and the obtained solid was washed with n-hex to yield 44.3 mg (83%) of **4b** as an amorphous violet solid. Mp: 216.4–217.4 °C; ^1^H-NMR (500 MHz, CDCl_3_) δ 0.86 (t, *J* = 6.9 Hz, 3H, H-11′’’), 1.23 (bs, 16H, H-3′’’-H-10′’’), 1.44 (m, 2H, H-2′’’), 2.38 (t, *J* = 7.8 Hz, 2H, H-1′’’); 5.48 (s, 1H, H-4); 7.10 (t, *J* = 7.3 Hz, 1H); 7.18 (t, *J* = 7.6 Hz, 2H); 7.27 (m, 2H); 7.33 (m, 5H); ^13^C-NMR (125 MHz, CDCl_3_) δ 14.1 (CH_3_), 22.6 (CH_2_), 22.7 (CH_2_), 28.2 (CH_2_), 29.3 (CH_2_), 29.5 (CH_2_), 29.6 (CH_2_ x 2), 29.7 (CH_2_ x 2), 31.9 (CH_2_), 36.2 (CH), 104.1 (C), 108.1 (C), 116.0 (C), 126.6 (CH), 126.8 (CH x 2), 128.3 (CH x 4), 128.8 (C), 128.9 (CH x 2), 129.0 (CH), 140.2 (C), 141.1 (C), 145.6 (C), 147.5 (C), 154.6 (C), 178.8 (C), 182.6 (C); EIMS *m*/*z* (%) 523 ([M^+^], 76), 446 (100), 382 (13), 276 (5); HREIMS 523.2852 (calcd for C_33_H_37_N_3_O_3_ [M^+^] 523.2835); IR v_max_ 3433 (N-H), 3259 (O-H), 2921 (C-H aliph), 2851 (C-H aliph), 1641 (C=O), 1571, 1528, 1506, 1483, 1437 (C=N, C=C), 1352 (N-N), 1271, 1235, 1182, 1138, 1084, 1029, 981, 878, 842 cm^−1^.

### 3.5. 4-(4-Chlorophenyl)-6-Hydroxy-3-Phenyl-7-Undecyl-1H-Pyrazolo[3,4-b]Quinoline-5,8 (4H,9H)-Dione (***4c***)

Following the general procedure described above, in a 5 mL MW tube, 30 mg of embelin (0.1 mmol), 21.5 mg of 4-chlorobenzaldehyde (0.15 mmol) and 24.4 mg of 3-amino-5-phenylpyrazole (0.15 mmol) were dissolved in 2 mL DCE and treated with 1.8 mg of EDDA (10 mol %). The tube was sealed, and the reaction mixture was irradiated at 150 °C for 10 min. Then, the reaction mixture was filtered and the obtained solid was washed with n-hex to yield 56.5 mg (98%) of **4c** as an amorphous blue solid. Mp: 220.3–221.1 °C; ^1^H-NMR (500 MHz, CDCl_3_) δ 0.86 (t, *J* = 7.0 Hz, 3H, H-11′’’), 1.23 (bs, 16H, H-3′’’-H-10′’’), 1.43 (m, 2H, H-2′’’), 2.39 (t, *J* = 7.6 Hz, 2H, H-1′’’), 5.48 (s, 1H, H-4), 7.14 (d, *J* = 8.4 Hz, 2H, H-3′’ + H-5′’), 7.20 (d, *J* = 8.4 Hz, 2H, H-2′’ + H-6′’), 7.39 (m, 5H, H-2′-H-5′); ^13^C-NMR (125 MHz, CDCl_3_) 14.1 (CH_3_), 22.6 (CH_2_), 22.7 (CH_2_), 28.2 (CH_2_), 29.3 (CH_2_), 29.5 (CH_2_), 29.6 (CH_2_ x 2), 29.7 (CH_2_ x 2), 31.9 (CH_2_), 35.7 (CH), 103.7 (C), 107.7 (C), 116.2 (C), 126.8 (CH x 2), 128.4 (CH x 2), 128.6 (C), 129.1 (CH x 2), 129.2 (CH), 129.5 (CH_2_ x 2), 132.4 (C), 149.1 (C), 141.0 (C), 143.9 (C), 147.4 (C), 154.4 (C), 178.8 (C), 182.35 (C); EIMS *m*/*z* (%) 541 ([M^+^], 100), 446 (8), 413 (19), 400 (68); HREIMS *m*/*z* 559.2476 (calc for C_33_H_36_N_3_O_3_^37^Cl [M^+^] 559.2416); 557.2471 (calcd for C_33_H_36_N_3_O_3_^35^Cl [M^+^] 557.2445); IR v_max_ 3428 (N-H), 3240 (O-H), 2924 (C-H aliph), 2853 (C-H aliph), 2322 (C-C arom), 1642 (C=O), 1587, 1528, 1484, 1437 (C=N, C=C), 1352 (N-N), 1272, 1205, 1182, 1138, 1089 (C-Cl), 1014, 982, 826 cm^−1^.

### 3.6. 4-(4-Bromophenyl)-6-Hydroxy-3-Phenyl-7-Undecyl-1H-Pyrazolo[3,4-b]Quinoline-5,8 (4H,9H)-Dione (***4d***)

Following the general procedure described above, in a 5 mL MW tube, 30 mg of embelin (0.1 mmol), 28.3 mg of 4-bromobenzaldehyde (0.15 mmol) and 24.4 mg of 3-amino-5-phenylpyrazole (0.15 mmol) were dissolved in 2 mL DCE and treated with 1.8 mg of EDDA (10 mol %). The tube was sealed, and the reaction mixture was irradiated at 150 °C for 10 min. Then, the reaction mixture was filtered and the obtained solid was washed with n-hex to yield 50.5 mg (82%) of **4d** as an amorphous violet solid. Mp: 231.8–232.5 °C; ^1^H-NMR (500 MHz, CDCl_3_) δ 0.86 (t, *J* = 7.1 Hz, 3H, H-11′’’), 1.23 (bs, 16H, H-3′’’-H-10′’’), 1.44 (m, 2H, H-2′’’), 2.39 (t, *J* = 7.9 Hz, 2H, H-1′’’), 5.47 (s, 1H, H-4), 7.13 (d, *J* = 8.3 Hz, 2H, H-3′’ + H-5′’), 7.30 (d, *J* = 8.3 Hz, 2H, H-2′’ + H-6′’), 7.32 (m, 2H), 7.38 (m, 3H); ^13^C-NMR (125 MHz, CDCl_3_) δ 14.1 (CH_3_), 22.6 (CH_2_), 22.7 (CH_2_), 28.1 (CH_2_), 29.3 (CH_2_), 29.5 (CH_2_), 29.6 (CH_2_ x 2), 29.6 (CH_2_), 29.7 (CH_2_), 31.9 (CH_2_), 35.7 (CH), 103.5 (C), 107.6 (C), 116.3 (C), 120.6 (C), 126.8 (CH x 2), 128.5 (C), 129.0 (C), 129.1 (CH_2_ x 2), 129.2 (CH), 129.9 (CH x 2), 131.4 (CH x 2), 140.1 (C), 140.9 (C), 144.4 (C), 147.4 (C), 178.7 (C), 182.3 (C); EIMS *m*/*z* (%) 601 ([M^+^], 47), 460 (7), 446 (100), 306 (7); HREIMS *m*/*z* 601.1951 (calc for C_33_H_36_N_3_O_3_^79^Br [M^+^] 601.1940); 603.1866 (calcd for C_33_H_36_N_3_O_3_^81^Br [M^+^] 603.1920); IR v_max_ 3389 (N-H), 3256 (O-H), 2923, 2852 (C-H aliph), 1640 (C=O), 1573, 1526, 1503, 1482 (C=N, C=C), 1351 (N-N), 1315, 1270, 1237, 1181, 1136, 1071 (C-Br), 1009, 984, 827 cm^−1^.

### 3.7. 4-(4-Fluorophenyl)-6-Hydroxy-3-Phenyl-7-Undecyl-1H-Pyrazolo[3,4-b]Quinoline-5,8 (4H,9H)-Dione (***4e***)

Following the general procedure described above, in a 5 mL MW tube, 30 mg of embelin (0.1 mmol), 19 mg of 4-fluorobenzaldehyde (0.15 mmol, 16.4 µL) and 24.4 mg of 3-amino-5-phenylpyrazole (0.15 mmol) were dissolved in 2 mL DCE and treated with 1.8 mg of EDDA (10 mol %). The tube was sealed, and the reaction mixture was irradiated at 150 °C for 10 min. Then, the reaction mixture was filtered and the obtained solid was washed with n-hex to yield 46.5 mg (84%) of **4e** as an amorphous violet solid. Mp: 234.5–235.4 °C; ^1^H-NMR (500 MHz, CDCl_3_) δ 0.86 (t, *J* = 7.1 Hz, 3H, H-11′’’), 1.23 (bs, 16H, H-3′’’-H-10′’’), 1.43 (m, 2H, H-2′’’), 2.40 (t, *J* = 7.5 Hz, 2H, H-11′), 5.50 (s, 1H, H-4), 6.86 (t, *J* = 8.5 Hz, 2H, H-3′’ + H-5′’), 7.22 (m, 2H, H-2′’ + H-6′’), 7.35 (m, 5H, H-2′-H-5′); ^13^C-NMR (125 MHz, CDCl_3_) 14.1 (CH_3_), 22.6 (CH_2_), 22.7 (CH_2_), 28.2 (CH_2_), 29.3 (CH_2_), 29.5 (CH_2_), 29.6 (CH_2_ x 2), 29.7 (CH_2_), 31.9 (CH_2_), 35.5 (CH), 104.0 (C), 107.8 (C), 115.1 (CH x 2, *J* = 21.4 Hz), 116.2 (C), 126.8 (CH x 2), 128.6 (C), 129.0 (CH x 2), 129.2 (CH), 129.7 (CH x 2, *J* = 7.9 Hz), 140.0 (C), 141.1 (C), 141.3 (C), 141.4 (C), 147.4 (C), 154.3 (C), 161.5 (C-F, *J* = 248.5 Hz), 178.7 (C), 182.6 (C); EIMS *m*/*z* (%) 541 ([M^+^], 96); 446 (100); 401 (13); 304 (8); HREIMS *m*/*z* 541.2744 (calcd for C_33_H_36_N_3_O_3_F [M^+^] 541.2741); IR v_max_ 3426 (N-H), 3234 (O-H), 2924, 2851 (C-H aliph), 1641 (C=O), 1571, 1528, 1483 (C=C, C=N), 1353 (N-N), 1295, 1272, 1223, 1205, 1155, 1137 (C-F), 1014, 983, 835 cm^−1^.

### 3.8. 4-(3-Fluorophenyl)-6-Hydroxy-3-Phenyl-7-Undecyl-1H-Pyrazolo[3,4-b]Quinoline-5,8 (4H,9H)-Dione (***4f***)

Following the general procedure described above, in a 5 mL MW tube, 30 mg of embelin (0.1 mmol), 19 mg of 3-fluorobenzaldehyde (0.15 mmol, 16.1 µL) and 24.4 mg of 3-amino-5-phenylpyrazole (0.15 mmol) were dissolved in 2 mL of DCE and treated with 1.8 mg of EDDA (10 mol %). The tube was sealed, and the reaction mixture was irradiated at 150 °C for 10 min. Then, the reaction mixture was filtered and the obtained solid was washed with n-Hex to yield 43 mg (79%) of **4f** as an amorphous violet solid. Mp: 224.7–225.5 °C; ^1^H-NMR (500 MHz, CDCl_3_) δ 0.86 (t, *J* = 7.0 Hz, 3H, H-11′’’), 1.23 (bs, 16H, H-3′’’-H-10′’’), 1.46 (m, 2H, H-2′’’), 2.40 (t, *J* = 7.8 Hz, 2H, H-11′), 5.51 (s, 1H, H-4), 6.80 (td, *J* = 1.8, 8.3 Hz, 1H, H-2′’), 6.96 (dt, *J* = 2.5, 9.6 Hz, 1H, H-4′’), 7.05 (d, *J* = 7.7 Hz, 1H, H-1′’), 7.14 (m, 1H), 7.32 (m, 2H), 7.37 (m, 3H); ^13^C-NMR (125 MHz, CDCl_3_) 14.2 (CH_3_), 22.6 (CH_2_), 22.7 (CH_2_), 28.2 (CH_2_), 29.3 (CH_2_), 29.5 (CH_2_), 29.6 (CH_2_ x 2), 29.7 (CH_2_ x 2), 31.9 (CH_2_), 35.8 (CH), 103.5 (C), 107.4 (C), 113.5 (C, *J* = 20.8 Hz), 115.2 (CH, *J* = 21.6 Hz), 116.2 (C), 123.8 (CH), 126.7 (CH x 4), 128.5 (C), 129.0 (CH), 129.2 (CH), 129.6 (CH, *J* = 7.8 Hz), 140.1 (C), 141.1 (C), 147.3 (C), 147.8 (C), 154.3 (C), 162.8 (C, *J* = 247.8 Hz), 178.6 (C), 182.4 (C); EIMS *m*/*z* (%) 541 ([M^+^], 79); 446 (100); 400 (18); 305 (9); HREIMS 541.2756 (calcd for C_33_H_36_N_3_O_3_F [M^+^] 541.2741); IR v_max_ 3429 (N-H), 3256 (O-H), 2924, 2851 (C-H aliph), 1639 (C=O), 1585, 1527, 1481,1439 (C=C, C=N), 1350 (N-N), 1296, 1199, 1145 (C-F), 983, 929, 841, 729, 690 cm^−1^.

### 3.9. 6-Hydroxy-3-Phenyl-4-(4-(Trifluoromethyl)Phenyl)-7-Undecyl-1H-Pyrazolo [3,4-b] Quinoline-5,8 (4H,9H)-Dione (***4g***)

Following the general procedure described above, in a 5 mL MW tube, 30 mg of embelin (0.1 mmol), 26.6 mg of 4-(trifluromethyl)-benzaldehyde (0.15 mmol, 21 µL) and 24.4 mg of 3-amino-5-phenylpyrazole (0.15 mmol) were dissolved in 2 mL of DCE and treated with 1.8 mg of EDDA (10 mol %). The tube was sealed, and the reaction mixture was irradiated at 150 °C for 10 min. Then, the reaction mixture was filtered and the obtained solid was washed with n-hex to yield 53.7 mg (89%) of **4g** as an amorphous violet solid. Mp: 213.9–214.9 °C; ^1^H-NMR (500 MHz, CDCl_3_) δ 0.87 (t, *J* = 7.1 Hz, 3H, H-11′’’), 1.22 (bs, 16H, H-3′’’-H-10′’’), 1.43 (m, 2H, H-2′’’), 2.37 (t, *J* = 7.1 Hz, 2H, H-1′’’), 5.55 (s, 1H, H-4), 7.31 (m, 2H), 7.35 (m, 5H), 7.40 (d, *J* = 8.0 Hz, 2H, H-3′’ + H-5′’); ^13^C-NMR (125 MHz, CDCl_3_) 14.1 (CH_3_), 22.7 (CH_2_ x 2), 28.2 (CH_2_), 29.3 (CH_2_), 29.5 (CH_2_), 29.6 (CH_2_ x 2), 29.7 (CH_2_ x 2), 31.9 (CH_2_), 36.2 (CH), 103.4 (C), 107.2 (C), 116.3 (C), 123.1 (C), 125.2 (CH x 2), 126.8 (CH x 2), 128.5 (CH x 2), 128.5 (C), 128.7 (C), 129.1 (CH x 2), 129.3 (CH), 140.2 (C), 141.5 (C), 147.3 (C), 149.2 (C), 179.2 (C), 182.1 (C); EIMS *m*/*z* (%) 591 ([M^+^], 100); 446 (95); 306 (8); 276 (6); HREIMS *m*/*z* 591.2720 (calcd for C_34_H_36_N_3_O_3_F_3_ [M^+^] 591.2709); IR v_max_ 3431 (N-H), 3253 (O-H), 2924, 2853 (C-H aliph), 1642 (C=O), 1586, 1569, 1529, 1484 (C=C, C=N), 1322, (N-N), 1272, 1236, 1161, 1120 (C-F), 1066, 1017, 986, 834 cm^−1^.

### 3.10. 6-Hydroxy-4-(3-Nitrophenyl)-3-Phenyl-7-Undecyl-1H-Pyrazolo[3,4-b]Quinoline-5,8 (4H,9H)-Dione (***4h***)

Following the general procedure described above, in a 5 mL MW tube, 30 mg of embelin (0.1 mmol), 23.4 mg of 3-nitrobenzaldehyde (0.15 mmol) and 24.4 mg of 3-amino-5-phenylpyrazole (0.15 mmol) were dissolved in 2 mL DCE and treated with 1.8 mg of EDDA (10 mol %). The tube was sealed, and the reaction mixture was irradiated at 150 °C for 10 min. Then, the reaction mixture was filtered and the obtained solid was washed with n-hex to yield 52 mg (90%) of **4h** as an amorphous violet solid. Mp: 210.2–211.8 °C; ^1^H-NMR (500 MHz, DMSO-d_6_) δ 0.85 (t, *J* = 7.1 Hz, 3H, H-11′’’), 1.22 (bs, 16H, H-3′’’-H-10′’’), 1.33 (m, 2H, H-2′’’), 2.25 (t, *J* = 7.5 Hz, 2H, H-1′’’), 3.03 (bs, 1H), 5.71 (s, 1H), 7.29 (t, *J* = 7.5 Hz, 1H), 7.37 (t, *J* = 7.5 Hz, 2H), 7.41 (t, *J* = 8.1 Hz,1H), 7.51 (d, *J* = 7.2 Hz, 2H), 7.58 (d, *J* = 7.8 Hz, 1H), 7.88 (dd, *J* = 1.3, 8.1 Hz, 1H), 8.00 (t, *J* = 2.1 Hz, 1H); ^13^C-NMR (125 MHz, DMSO-d_6_) 13.9 (CH_3_), 22.0 (CH_2_), 22.1(CH_2_), 27.7 (CH_2_), 28.7 (CH_2_), 28.8 (CH_2_), 28.9 (CH_2_), 29.0 (CH_2_ x 2), 29.1 (CH_2_), 31.3 (CH_2_), 35.9 (CH), 101.8 (C), 106.5 (C), 115.5 (C), 121.0 (CH), 122.3 (CH), 126.4 (CH x 2), 128.2 (CH), 128.6 (CH x 2), 129.4 (CH), 137.6 (CH), 138.7 (C), 140.5 (C), 146.2 (C), 147.1 (C), 148.1 (C), 157.6(C), 179.0 (C), 180.4 (C); EIMS *m*/*z* (%) 568 ([M^+^], 92); 538 (7); 446 (100); 427(20); 306 (8). HREIMS 568.2669 (calcd for C_33_H_36_N_4_O_5_ [M^+^] 568.2686); IR 3415 (N-H), 3267 (O-H), 2924, 2851 (C-H aliph), 1639 (C=O), 1578, 1527, 1485 (C=C, C=N), 1346 (N-N), 1315 (NO_2_), 1269, 1192, 1123, 1088, 814, 690 cm^−1^.

### 3.11. 4-(6-Hydroxy-5,8-Dioxo-3-Phenyl-7-Undecyl-4,5,8,9-Tetrahydro-1H-Pyrazolo[3,4-b]Quinolin-4-yl)Benzoic Acid (***4i***)

Following the general procedure described above, in a 5 mL MW tube, 30 mg of embelin (0.1 mmol), 23 mg of 4-formylbenzoic acid (0.15 mmol, 14.4 μL) and 24.4 mg of 3-amino-5-phenylpyrazole (0.154 mmol) were dissolved in 2 mL of DCE and treated with 1.8 mg of EDDA (10 mol %). The tube was sealed, and the reaction mixture was irradiated at 150 °C for 10 min. Then, the reaction mixture was filtered and the obtained solid was washed with n-hex to yield 54.7 mg (93%) of **4i** as an amorphous violet solid. Mp: 240.1–242.0 °C; ^1^H-NMR (500 MHz, DMSO-d_6_) δ 0.77 (t, *J* = 7.1 Hz, 3H, H-11′’’), 1.13 (bs, 16H, H-3′’’-H-10′’’), 1.30 (m, 2H, H-2′’’), 2.23 (t, *J* = 7.6 Hz, 2H, H-1′’’), 5.54 (s, 1H, H-4), 7.23 (d, *J* = 8.3 Hz, 2H, H-2′’ + H-6′’), 7.30 (m, 1H, H-4′), 7.37 (t, *J* = 7.5 Hz, 2H, H-3′ + H-5′), 7.46 (d, *J* = 7.5 Hz, 2H, H-2′ + H-6′), 7.66 (d, *J* = 8.2 Hz, 2H, H-3′’ + H-5′’); ^13^C-NMR (125 MHz, DMSO-d_6_) 14.1 (CH_3_), 22.0 (CH_2_ x 2), 27.7 (CH_2_), 28.7 (CH_2_), 28.8 (CH_2_), 28.9 (CH_2_), 29.0 (CH_2_ x 2), 29.1 (CH_2_), 31.3 (CH_2_), 36.0 (CH), 102.2 (C), 107.1 (C), 115.3 (C), 124.6 (C), 124.9 (C), 126.2 (CH x 2), 128.0 (CH x 2), 128.1 (CH), 128.5 (C), 128.7 (CH x 2), 129.0 (CH x 2), 129.8 (C), 138.5 (C), 140.3 (C), 150.9 (C), 167.0 (C), 177.3 (C), 179.0 (C); EIMS *m*/*z* (%) 567 ([M^+^], 38), 566 (100), 551 (62), 446 (96), 410 (31); HREIMS 567.2757 (calcd for C_34_H_37_N_3_O_5_ [M^+^] 567.2733); IR v_max_ 3413 (OH), 2375 (C-H arom), 2924, 2854 (C-H aliph), 1685 (C=O), 1574, 1527, 1427 (C=C, C=N), 1366 (N-N), 1258, 1184, 1141, 976, 860, 694 cm^−1^.

### 3.12. Methyl-4-(6-Hydroxy-5,8-Dioxo-3-Phenyl-7-Undecyl-4,5,8,9-Tetrahydro-1H-Pyrazolo[3,4-b]Quinolin-4-yl)Benzoate (***4j***)

Following the general procedure described above, in a 5 mL MW tube, 33 mg of embelin (0.112 mmol), 24.4 mg of methyl 4-formylbenzoate (0.17 mmol) and 27.1 mg of 3-amino-5-phenylpyrazole (0.17 mmol) were dissolved in 2 mL of DCE and treated with 2 mg of EDDA (10 mol %). The tube was sealed, and the reaction mixture was irradiated at 150 °C for 10 min. Then, the reaction mixture was filtered and the obtained solid was washed with n-hex to yield 62.2 mg (95%) of **4j** as an amorphous violet solid. Mp: 219.1–220.4 °C. ^1^H-NMR (500 MHz, CDCl_3_) δ 0.86 (t, *J* = 7.1 Hz, 3H, H-11′’’), 1.23 (bs, 16H, H-3′’’-H-10′’’), 1.45 (m, 2H, H-2′’’), 2.40 (t, *J* = 8.2 Hz, 2H, H-1′), 3.85 (s, 3H, -OCH_3_), 5.57 (s, 1H, H-4), 7.30 (m, 2H), 7.34 (d, *J* = 8.3 Hz, 2H, H-2′’ + H-6′’), 7.37 (m, 3H), 7.86 (d, *J* = 8.3 Hz, 2H, H-3′’ + H-5′’); ^13^C-NMR (125 MHz, CDCl_3_) δ 14.1 (CH_3_), 22.6 (CH_2_), 22.7 (CH_2_), 28.1 (CH_2_), 29.3 (CH_2_), 29.5 (CH_2_), 29.6 (CH_2_ x 2), 29.7 (CH_2_ x 2), 31.9 (CH_2_), 36.2 (CH), 52.0 (CH_3_), 103.4 (C), 107.2 (C), 116.3 (C), 126.8 (CH x 2), 128.2 (CH x 2), 128.4 (C), 128.5 (C), 129.1 (CH x 2), 129.3 (CH), 129.7 (CH x 2), 140.2 (C), 141.1 (C), 147.3 (C), 150.2 (C), 154.0 (C), 166.9 (C), 178.5 (C), 182.4 (C); EIMS *m*/*z* (%) 581 ([M^+^], 78); 446 (100); 305 (59); 159 (47); HREIMS 581.2916 (calcd for C_35_H_39_N_3_O_5_ [M^+^] 581.2890); IR v_max_ 3433 (OH), 2923, 2854 (C-H aliph), 2360 (C-H arom), 1639 (C=O), 1589, 1569, 1523, 1485 (C=C, C=N), 1353 (N-N), 1273, 1218, 1138, 1068, 995, 891, 690 cm^−1^.

### 3.13. 4-(6-Hydroxy-5,8-Dioxo-3-Phenyl-7-Undecyl-4,5,8,9-Tetrahydro-1H-Pyrazolo[3,4-b]Quinolin-4-yl)Benzonitrile (***4k***)

Following the general procedure described above, in a 5 mL MW tube, 30 mg of embelin (0.1 mmol), 20.1 mg of 4-cyanobenzaldehyde (0.15 mmol, 14.4 μL) and 24.4 mg of 3-amino-5-phenylpyrazole (0.154 mmol) were dissolved in 2 mL of DCE and treated with 1.8 mg of EDDA (10 mol %). The tube was sealed, and the reaction mixture was irradiated at 150 °C for 10 min. Then, the reaction mixture was filtered and the obtained solid was washed with n-hex to yield 45.3mg (81%) of **4k** as an amorphous violet solid. Mp: 206.2–207.8 °C; ^1^H-NMR (500 MHz, CDCl_3_) δ 0.86 (t, *J* = 7.1 Hz, 3H, H-11′’’), 1.23 (bs, 16H, H-3′’’-H-10′’’), 1.45 (m, 2H, H-2′’’), 2.40 (t, *J* = 7.7 Hz, 2H, H-1′’’), 5.56 (s, 1H, H-4), 7.29 (m, 2H), 7.34 (d, *J* = 8.1 Hz, 2H, H-2′’ + H-6′’), 7.38 (m, 3H), 7.45 (d, *J* = 8.1 Hz, 2H, H-3′’ + H-5′’); ^13^C-NMR (125 MHz, CDCl_3_) 14.1 (CH_3_), 22.6 (CH_2_), 22.7 (CH_2_), 28.1 (CH_2_), 29.3 (CH_2_), 29.5 (CH_2_), 29.6 (CH_2_ x 2), 29.7 (CH_2_ x 2), 31.9 (CH_2_), 36.5 (CH), 103.0 (C), 106.6 (C), 110.4 (C), 116.4 (C), 118.8 (C), 125.3 (C), 126.8 (CH x 2), 128.2 (C), 128.9 (CH x 2), 129.1 (CH x 2), 129.4 (CH), 132.1 (CH x 2), 140.3 (C), 141.4 (C), 147.1 (C), 150.2(C), 178.6 (C), 182.2 (C); EIMS *m*/*z* (%) 548 ([M^+^], 69), 532 (37), 395 (37), 274 (100), 159 (73); HREIMS 548.2787 (calcd for C_34_H_36_N_4_O_3_ [M^+^] 548.2787); IR v_max_ 3256 (OH), 2920, 2854 (CH-arom), 2804, 2677, 2229 (CN), 2052, 1643 (C=O), 1578, 1519, 1504, 1438 (C=C, C=N), 1350 (N-N), 1196, 1138, 1084, 986, 833, 694 cm^−1^.

### 3.14. 4-(4-(Dimethylamino)Phenyl)-6-Hydroxy-3-Phenyl-7-Undecyl-1H-Pyrazolo[3,4-b]Quinoline-5,8 (4H,9H)-Dione (***4l***)

Following the general procedure described above, in a 5 mL MW tube, 35 mg of embelin (0.12 mmol), 27 mg of 4-dimethylaminobenzaldehyde (0.18 mmol) and 28.7 mg of 3-amino-5-phenylpyrazole (0.18 mmol) were dissolved in 2 mL of DCE and treated with 3.4 mg of EDDA (10 mol %). The tube was sealed, and the reaction mixture was irradiated at 150 °C for 10 min. Then, the reaction mixture was filtered and the obtained solid was washed with n-hex to yield 59.9 mg (88%) of **4l** as an amorphous violet solid. Mp: 195.8–197.2 °C; ^1^H-NMR (500 MHz, CDCl_3_) δ 0.87 (t, *J* = 7.1 Hz, 3H, H-11′’’), 1.24 (bs, 16H, H-3′’’-H-10′’’), 1.45 (m, 2H, H-2′’’), 2.39 (t, *J* = 7.7 Hz, 2H, H-1′’’), 2.87 (s, 6H, -N(CH_3_)_2_), 3.07 (bs, 1H, -NH), 5.41 (s, 1H, H-4), 7.31 (m, 2H), 6.57 (d, *J* = 8.7 Hz, 2H, H-3′’ + H-5′’), 7.14 (d, *J* = 8.5 Hz, 2H, H-2′’ + H-6′’), 7.36 (m, 5H, H-2′-H-5′); ^13^C-NMR (125 MHz, CDCl_3_) δ 14.1 (CH_3_), 22.5 (CH_2_), 22.7 (CH_2_), 28.1 (CH_2_), 29.3 (CH_2_), 29.5 (CH_2_), 29.6 (CH_2_ x 2), 29.7 (CH_2_), 31.9 (CH_2_), 34.9 (CH_2_), 40.5 (CH), 104.4 (C), 108.8 (C), 112.3 (CH x 2), 115.8 (C), 125.3 (CH), 125.5 (C), 126.7 (CH x 2), 128.9 (CH x 2), 129.0 (CH x 2), 129.1 (C), 130.8 (C), 133.9 (C), 139.6 (C), 140.3 (C), 147.7 (C), 149.1 (C), 154.0 (C), 178.9 (C), 182.8 (C); EIMS *m*/*z* (%) 566 ([M^+^], 100), 550 (19), 447 (16), 426 (14), 290 (12); HREIMS 566.3282 (calcd for C_35_H_42_N_4_O_3_ [M^+^] 566.3257); IR v_max_ 3433 (N-H), 3251 (O-H), 2920, 2851, 2804 (C-H arom), 1666 (C=O), 1520, 1481, 1438 (C=C, C=N), 1354 (N-N), 1315, 1273, 1199, 1126, 1061, 1034, 964, 814, 690 cm^−1^.

### 3.15. 4-(3-Fluoro-4-Methoxyphenyl)-6-Hydroxy-3-Phenyl-7-Undecyl-1H-Pyrazolo[3,4-b]Quinoline-5,8 (4H,9H)-Dione (***4m***)

Following the general procedure described above, in a 5 mL MW tube, 30 mg of embelin (0.1 mmol), 23.6 mg of 3-fluoro-4-methoxybenzaldehyde (0.15mmol) and 24.4 mg of 3-amino-5-phenylpyrazole (0.15 mmol) were dissolved in 2 mL of DCE and treated with 1.8 mg of EDDA (10 mol %). The tube was sealed, and the reaction mixture was irradiated at 150 °C for 10 min. Then, the reaction mixture was filtered and the obtained solid was washed with n-hex to yield 57.6 mg (98%) of **4m** as an amorphous violet solid. Mp: 189.7–190.1 °C; ^1^H-NMR (500 MHz, CDCl_3_) δ 0.86 (t, *J* = 7.1 Hz, 3H, H-11′’’), 1.22 (bs, 16H, H-3′’’-H-10′’’), 1.43 (m, 2H, H-2′’’), 2.38 (t, *J* = 7.6 Hz, 2H, H-1′’’), 3.77 (s, 3H, -OCH_3_), 5.44 (s, 1H, H-4), 6.73 (t, *J* = 8.8 Hz, 1H), 6.98 (m, 2H), 7.51 (m, 5H); ^13^C-NMR (125 MHz, CDCl_3_) δ 14.1 (CH_3_), 22.6 (CH_2_), 22.7 (CH_2_), 28.2 (CH_2_), 29.3 (CH_2_), 29.5 (CH_2_), 29.6 (CH_2_ x 2), 29.7 (CH_2_), 29.7 (CH_2_), 31.9 (CH_2_), 35.3 (CH), 56.2 (CH_3_), 103.7 (C), 107.7 (C), 113.0 (C), 116.0 (CH, *J* = 19.2 Hz), 123.7 (CH, *J* = 2.6 Hz), 126.7 (CH x 2), 128.7 (C), 129.0 (CH x 2), 129.0 (CH), 138.7 (C, *J* = 4.8 Hz), 139.9 (C), 141.1 (C) 146.2 (C, *J* = 10.6 Hz), 147.4 (C), 151.2 (C), 154.0 (C-F, *J* = 239.4 Hz), 154.9 (C), 178.9 (C), 182.43 (C); EIMS *m*/*z* (%) 571 ([M^+^], 99); 446 (100); 295 (82); 159 (94); HREIMS 571.2845 (calcd for C_34_H_38_N_3_O_4_F [M^+^] 571.2846); IR v_max_ 3425 (N-H), 3251 (O-H), 2924, 2852 (C-H arom), 1641 (C=O), 1586, 1571, 1506, 1436 (C=N, C=C), 1352 (N-N), 1314, 1269, 1201, 1148, 1116, 1028, 981, 872, 803 cm^−1^.

### 3.16. 4-(3,4-Dimethoxyphenyl)-6-Hydroxy-3-Phenyl-7-Undecyl-1H-Pyrazolo[3,4-b]Quinoline-5,8 (4H,9H)-Dione (***4n***)

Following the general procedure described above, in a 5 mL MW tube, 30 mg of embelin (0.1 mmol), 25.4 mg of 3,4-dimethoxybenzaldehyde (0.15 mmol) and 24.4 mg of 3-amino-5-phenylpyrazole (0.15 mmol) were dissolved in 2 mL of DCE and treated with 1.8 mg of EDDA (10 mol %). The tube was sealed, and the reaction mixture was irradiated at 150 °C for 10 min. Then, the reaction mixture was filtered and the obtained solid was washed with n-hex to yield 54.2 mg (91%) of **4n** as an amorphous violet solid. Mp: 183.8–184.5 °C; ^1^H-NMR (500 MHz, CDCl_3_) δ 0.86 (t, *J* = 7.1 Hz, 3H, H-11′’’), 1.23 (bs, 16H, H-3′’’-H-10′’’), 1.44 (m, 2H), 2.40 (t, *J* = 7.8 Hz, 2H, H-1′’’), 3.72 (s, 3H, -OCH_3_), 3.78 (s, 3H, -OCH_3_), 5.47 (s, 1H, H-4), 6.68 (d, *J* = 8.4 Hz, 1H, H-5′’), 6.75 (dd, *J* = 8.3, 1.8 Hz, 1H, H-6′’), 6.82 (d, *J* = 1.8 Hz, 1H, H-2′’), 7.37 (m, 5H, H-2′-H-6′); ^13^C-NMR (125 MHz, CDCl_3_) 14.1 (CH_3_), 22.6 (CH_2_), 22.7 (CH_2_), 28.2 (CH_2_), 29.3 (CH_2_), 29.5 (CH_2_), 29.6 (CH_2_ x 2), 29.7 (CH_2_ x 2), 31.9 (CH_2_), 35.6 (CH), 55.8 (CH_3_), 55.9 (CH_3_), 104.3 (C), 108.1 (C), 111.1 (CH), 111.7 (CH), 116.1 (C), 120.1 (CH), 127.0 (CH x 2), 128.9 (C), 129.0 (CH x 2), 129.1 (CH), 138.4 (C), 139.9 (C), 141.1 (C), 147.5 (C), 147.7 (C), 148.7 (C), 154.3 (C), 178.9 (C), 182.8 (C); EIMS *m*/*z* (%) 583 ([M^+^], 66); 446 (100); 304 (26); HREIMS *m*/*z* 583.3026 (calc for C_35_H_41_N_3_O_5_ [M^+^] 583.3046); IR v_max_ 3431 (N-H), 3253 (O-H), 2922, 2852 (C-H arom), 1641 (C=O), 1586, 1506 (C=C, C=N), 1352 (N-N), 1263 (O-CH_3_), 1233, 1203, 1136, 1029, 982, 927, 852 cm^−1^.

### 3.17. 4-(Benzo[d][1,3]Dioxol-5-yl)-6-Hydroxy-3-Phenyl-7-Undecyl-1H-Pyrazolo[3,4-b]Quinoline-5,8 (4H, 9H)-Dione (***4o***)

Following the general procedure described above, in a 5 mL MW tube, 30 mg of embelin (0.1 mmol), 23 mg of piperonal (0.15 mmol) and 24.4 mg of 3-amino-5-phenylpyrazole (0.15 mmol) were dissolved in 2 mL of DCE and treated with 1.8 mg of EDDA (10 mol %). The tube was sealed, and the reaction mixture was irradiated at 150 °C for 10 min. Then, the reaction mixture was filtered and the obtained solid was washed with n-hex to yield 42.3 mg (73%) of **4o** as an amorphous violet solid. Mp: 227.5–228.3 °C; ^1^H-NMR (500 MHz, CDCl_3_) δ 0.86 (t, *J* = 7.1 Hz, 3H, H-11′’’), 1.23 (bs, 16H, H-3′’’-H-10′’’), 1.44 (m, 2H, H-2′’’), 2.39 (t, *J* = 7.7 Hz, 2H, H-1′’’), 5.43 (s, 1H, H-4), 5.86 (d, *J* = 4.2 Hz, 2H, -OCH_2_O-), 6.62 (d, *J* = 7.8 Hz, 1H, H-6′’), 6.75 (m, 2H), 7.37 (m, 5H); ^13^C-NMR (125 MHz, CDCl_3_) 14.1 (CH_3_), 22.6 (CH_2_), 22.7 (CH_2_), 28.2 (CH_2_), 29.3 (CH_2_), 29.5 (CH_2_), 29.6 (CH_2_ x 2), 29.7 (CH_2_ x 2), 31.9 (CH_2_), 35.7 (CH), 100.9 (CH_2_), 104.1 (C), 107.9 (C), 108.2 (C), 108.8 (C), 116.1 (C), 121.4 (C), 126.8 (CH x 2), 128.7 (CH), 129.0 (CH x 2), 129.1 (CH), 139.6 (CH), 139.9 (CH), 140.7 (C), 146.2 (C), 147.5 (C), 147.7 (C), 154.4 (C), 178.8 (C), 182.5 (C); EIMS *m*/*z* (%) 567 ([M^+^], 67), 446 (100), 427 (7), 304 (7); HREIMS *m*/*z* 567.2746 (calcd for C_34_H_37_N_3_O_5_ [M^+^] 567.2733); IR v_max_ 3427 (N-H), 3237 (O-H), 2923, 2853 (CH-aliph), 1641, (C=O), 1571, 1528, 1483, 1438 (C=C, C=N), 1315 (N-N), 1271, 1201, 1142, 1090, 1038, 981, 939, 922, 866 cm^−1^.

### 3.18. 6-Hydroxy-4-(1H-Imidazol-4-yl)-3-Phenyl-7-Undecyl-1H-Pyrazolo[3,4-b]Quinoline-5,8 (4H,9H)-Dione (***4p***)

Following the general procedure described above, in a 5 mL MW tube, 30 mg of embelin (0.1 mmol), 24.7 mg of 4-(5)-imidazolecarboxaldehyde (0.15 mmol) and 24.4 mg of 3-amino-5-phenylpyrazole (0.15 mmol) were dissolved in 2 mL of DCE and treated with 1.8 mg of EDDA (10 mol %). The tube was sealed, and the reaction mixture was irradiated at 150 °C for 10 min. Then, the reaction mixture was filtered and the obtained solid was washed with n-hex to yield 30 mg (57%) of **4p** as an amorphous violet solid. Mp: 265.4–266.1 °C; ^1^H-NMR (500 MHz, DMSO-d_6_) δ 0.88 (t, *J* = 7.1 Hz, 3H, H-11′’’), 1.26 (bs, 16H, H-3′’’-H-10′’’), 1.39 (m, 2H, H-2′’’), 2.30 (t, *J* = 7.7 Hz, 2H, H-1′’’), 5.58 (s, 1H, H-4), 6.75 (s, 1H, H-2′’), 7.38 (t, *J* = 7.4 Hz, 1H), 7.46 (m, 3H), 7.68 (d, *J* = 7.5 Hz, 2H); ^13^C-NMR (125 MHz, DMSO-d_6_) δ 13.9 (CH_3_), 22.0 (CH_2_ x 2), 27.8 (CH_2_), 28.4 (CH_2_), 28.6 (CH_2_), 28.9 (CH_2_ x 3), 29.0 (CH_2_) 29.1 (CH_2_), 31.2 (CH), 101.6 (C), 104.9 (C), 106.1 (C), 115.1 (C), 126.1 (CH x 2), 127.9 (CH), 128.7 (CH x 2), 133.9 (CH), 137.8 (CH), 140.5 (C), 146.9 (C), 157.6 (C), 178.9 (C), 181.0 (C); EIMS *m*/*z* (%) 513 ([M^+^], 50), 497 (100), 357 (29), 342 (7); HREIMS 513.2764 (calcd for C_30_H_35_N_5_O_3_ [M^+^] 513.2740); IR v_max_ 3420 (N-H), 3147 (O-H), 2921, 2850 (C-H aliph), 1637 (C=O), 1566, 1522, 1505, 1435 (C=C, C=N), 1360 (N-N), 1310, 1200, 1141, 1096, 980, 955, 843 cm^−1^.

### 3.19. 6-Hydroxy-3-Phenyl-4-(Pyridin-3-yl)-7-Undecyl-1H-Pyrazolo[3,4-b]Quinoline-5,8 (4H,9H)-Dione (***4r***)

Following the general procedure described above, in a 5 mL MW tube, 30 mg of embelin (0.1 mmol), 16.4 mg of 3-pyridinecarboxaldehyde (0.15 mmol, 14.4 μL) and 24.4 mg of 3-amino-5-phenylpyrazole (0.15 mmol) were dissolved in 2 mL of DCE and treated with 1.8 mg of EDDA (10 mol %). The tube was sealed, and the reaction mixture was irradiated at 150 °C for 10 min. Then, the reaction mixture was filtered and the obtained solid was washed with n-hex to yield 30.4 mg (57%) of **4r** as an amorphous violet solid. Mp: 273.6–274.6 °C; ^1^H-NMR (500 MHz, DMSO-d_6_) δ 0.81 (t, *J* = 7.0 Hz, 3H, H-11′’’), 1.19 (bs, 16H, H-3′’’-H-10′’’), 1.32 (m, 2H, H-2′’’), 2.25 (t, *J* = 7.1 Hz, 2H, H-1′’’), 5.56 (s, 1H, H-4), 7.14 (dd, *J* = 4.8, 7.8 Hz, 1H, H-6′’’), 7.30 (m, 1H), 7.37 (t, *J* = 7.4 Hz, 2H), 7.44 (dt, *J* = 1.9, 7.9 Hz, 1H), 7.49 (d, *J* = 7.6 Hz, 1H), 8.17 (dd, *J* = 1.4, 4.6 Hz, 1H, H-4′’), 8.39 (d, *J* = 1.8 Hz, 1H, H-2′’); ^13^C-NMR (125 MHz, DMSO-d_6_) δ 13.9 (CH_3_), 21.9 (CH_2_), 22.0 (CH_2_), 27.5 (CH_2_), 28.6 (CH_2_), 28.7 (CH_2_), 28.8 (CH_2_ x 2), 29.0 (CH_2_ x 2), 31.2 (CH_2_), 33.6 (CH), 101.9 (C), 106.9 (C), 115.9 (C), 123.5 (CH), 123.9 (C), 126.2 (CH x 2), 128.3 (CH), 128.7(CH x 2), 135.3 (CH), 137.0 (C), 140.0 (C), 141.3 (C), 147.0(CH), 148.6 (CH), 150.1 (C), 153.2(C), 178.6 (C), 181.4 (C); ESMS (-) *m*/*z* (%) 523 ([M-H]^+^, 30), 511 (100), 497(12), 391 (45); ESHRMS(-) 523.2708 (calcd for C_32_H_35_N_4_O_3_ [M^+^] 523.2709); IR v_max_ 3433 (OH), 2924, 2851 (C-H-alipha), 1639, (C=O), 1570, 1531, 1481, 1435 (C=C, C=N), 1357 (N-N), 1238, 1177, 1126, 1034, 976, 822, 694 cm^−1^.

### 3.20. 6-Hydroxy-3-Phenyl-4-(Pyridin-4-yl)-7-Undecyl-1H-Pyrazolo[3,4-b]Quinoline-5,8 (4H,9H)-Dione (***4s***)

Following the general procedure described above, in a 5 mL MW tube, 30 mg of embelin (0.1 mmol), 16.4 mg of 4-pyridinecarboxaldehyde (0.15 mmol, 14.4 μL) and 24.4 mg of 3-amino-5-phenylpyrazole (0.15 mmol) were dissolved in 2 mL DCE and treated with 1.8 mg of EDDA (10 mol %). The tube was sealed, and the reaction mixture was irradiated at 150 °C for 10 min. Then, the reaction mixture was filtered and the obtained solid was washed with n-hex to yield 33.8 mg (63%) of **4s** as an amorphous violet solid. Mp: 275.9–277.0 °C. ^1^H-NMR (500 MHz, DMSO-d_6_) δ 0.85 (t, *J* = 6.9 Hz, 3H, H-11′’’), 1.22 (bs, 16H, H-3′’’-H-10′’’), 1.34 (m, 2H, H-2′’’), 2.26 (t, *J* = 7.7 Hz, 2H, H-1′’’), 3.10 (bs, 1H, NH), 5.54 (s, 1H, H-4), 7.12 (d, *J* = 6.0 Hz, 1H, H-2′’ + H-4′’), 7.32 (m, 1H, H-4′), 7.40 (t, *J* = 7.8 Hz, 2H, H-3′ + H-5′), 7.52 (d, *J* = 7.2 Hz, 2H, H-2′ + H-6′), 8.29 (d, *J* = 6.0 Hz, 2H, H-3′’ + H-5′’); ^13^C-NMR (125 MHz, DMSO-d_6_) 13.9 (CH_3_), 21.9 (CH_2_), 22.0 (CH_2_), 27.6 (CH_2_), 28.7 (CH_2_), 28.8 (CH_2_), 28.9 (CH_2_ x 2), 29.0 (CH_2_ x 2), 31.2 (CH_2_), 35.6 (CH), 101.3 (C), 106.3 (C), 116.0 (C), 121.9 (C), 122.0 (CH x 2), 123.1 (C), 126.3 (CH x 2), 128.2 (CH), 128.7 (CH x 2), 140.3 (C), 149.1 (CH x 2), 150.2 (C), 150.4 (C), 154.0 (C), 178.5 (C), 181.4 (C); EIMS *m*/*z* (%) 524 ([M-H]^+^,100), 508 (100), 446 (43), 384 (33), 368 (70); HREIMS 524.2786 (calcd for C_32_H_36_N_4_O_3_ [M^+^] 524.2787). IR v_max_ 3433 (O-H), 2920, 2851 (C-H alipha), 1643 (C=O), 1589, 1531, 1481, 1439 (C=C, C=N), 1358 (N-N), 1269, 1142, 1007, 960, 791, 698, 679 cm^−1^.

### 3.21. 4-Cyclohexyl-6-Hydroxy-3-Phenyl-7-Undecyl-1H-Pyrazolo[3,4-b]Quinoline-5,8 (4H,9H)-Dione (***4t***)

Following the general procedure described above, in a 5 mL MW tube, 30 mg of embelin (0.1 mmol), 17.2 mg of cyclohexanaldehyde (0.15 mmol, 18.5 µL) and 24.4 mg of 3-amino-5-phenylpyrazole (0.15 mmol) were dissolved in 2 mL of DCE and treated with 1.8 mg of EDDA (10 mol %). The tube was sealed, and the reaction mixture was irradiated at 150 °C for 10 min. The crude was purified by Sephadex LH-20 using hex/DCM/MeOH (2:2:1) as eluent to yield 27.2 mg (50%) of **4t** as an amorphous blue solid. Mp: 184.6–185.2 °C; ^1^H-NMR (500 MHz, CDCl_3_) 0.64 (m, 2H), 0.87 (t, *J* = 7.1 Hz, 3H, H-11′’’), 0.98 (m, 2H), 1.25 (bs, 18H, H-3′’’-H-10′’’), 1.50 (m, 7H), 2.44 (t, *J* = 7.5 Hz, 2H), 4.50 (d, *J* = 3.3 Hz, 1H, H-4), 7.43 (t, *J* = 7.3 Hz, 1H), 7.49 (t, *J* = 7.7 Hz, 2H), 7.57 (d, *J* = 7.6 Hz, 2H, H-2′ + H-6′); ^13^C-NMR (125 MHz, CDCl_3_) 14.1 (CH_3_), 22.6 (CH_2_), 22.7 (CH_2_), 26.2 (CH_2_), 26.4 (CH_2_), 26.5 (CH_2_), 28.2 (CH_2_), 28.4 (CH_2_), 29.4 (CH_2_), 29.5 (CH_2_), 29.6 (CH_2_), 29.7 (CH_2_ x 3), 30.4 (CH_2_), 31.9 (CH_2_), 35.2 (CH), 46.4 (CH), 101.9 (C), 107.2 (C), 115.8 (C), 125.6 (C), 127.0 (CH x 2), 129.0 (CH), 129.2 (CH x 2), 130.2 (C), 139.9 (C); 143.1 (C), 149.1 (C), 154.0 (C), 179.1 (C), 182.5 (C); EIMS *m*/*z* (%) 529 ([M^+^], 2); 446 (100); 307 (10); 291 (9); HREIMS 529.3300 (calcd for C_33_H_43_N_3_O_3_ [M^+^] 529.3304); IR v_max_ 3265 (O-H), 2923, 2852 (C-H aliphat), 1637 (C=O), 1587, 1572, 1527, 1491, 1439 (C=C, C=N), 1387 (N-N), 1296, 1271, 1243, 1206, 1150, 1121, 1074, 977, 941, 893 cm^−1^.

### 3.22. 4-Ethyl-6-Hydroxy-3-Phenyl-7-Undecyl-1H-Pyrazolo[3,4-b]Quinoline-5,8 (4H,9H)-Dione (***4u***)

Following the general procedure described above, in a 5 mL MW tube, 30 mg of embelin (0.1 mmol), 8.9 mg of propanaldehyde (0.15 mmol, 11.1 µL) and 24.4 mg of 3-amino-5-phenylpyrazole (0.15 mmol) were dissolved in 2 mL of DCE and treated with 1.8 mg of EDDA (10 mol %). The tube was sealed, and the reaction mixture was irradiated at 150 °C for 10 min. The product was purified by Sephadex LH-20 using hex/DCM/MeOH (2:2:1) as eluent mixture to yield 18.3 mg (38%) of **4u** as an amorphous blue solid. Mp: 213.4–214.4 °C; ^1^H-NMR (500 MHz, DMSO-d_6_) δ 0.46 (t, *J* = 7.1 Hz, 3H, -CH_2_CH_3_), 0.85 (t, *J* = 7.1 Hz, 3H, H-11′’’), 1.23 (bs, 17H), 1.39 (m, 2H), 1.67 (m, 1H), 2.30 (t, *J* = 7.7 Hz, 2H), 4.61 (t, *J* = 4.2 Hz, 1H, H-4), 7.40 (t, *J* = 7.4 Hz, 1H, H-4′), 7.52 (t, *J* = 7.9 Hz, 2H, H-3′ + H-5′), 7.65 (d, *J* = 7.4 Hz, 2H, H-2′ + H-6′); ^13^C-NMR (125 MHz, DMSO-d_6_) δ 8.7 (CH_3_), 13.9 (CH_3_), 21.9 (CH_2_), 22.0 (CH_2_), 27.1 (CH_2_), 27.6 (CH_2_), 28.6 (CH_2_), 28.8 (CH_2_), 28.9 (CH_2_ x 4), 30.2 (CH_2_), 31.2 (CH), 101.3 (C), 106.0 (C), 115.5 (C), 124.7 (C), 126.1 (CH x 2), 128.1 (CH), 128.9 (CH x 2), 137.6 (C), 141.8 (C), 147.5 (C), 155.6 (C), 179.0 (C), 182.06 (C); EIMS *m*/*z* (%) 475 ([M^+^], 3), 446 (100), 318 (8), 306 (7); HREIMS *m*/*z* 475.2836 (calcd for C_29_H_37_N_3_O_3_ [M^+^] 475.2836); IR v_max_ 3442 (N-H), 3318 (O-H), 2917, 2849 (C-H-aliphat), 1637, (C=O), 1562, 1526, 1483 (C=C, C=N), 1359 (N-N), 1330, 1270, 1228, 1204, 1146, 1120, 1024, 980, 909, 871, 832 cm^−1^.

### 3.23. 4-Heptyl-6-Hydroxy-3-Phenyl-7-Undecyl-1H-Pyrazolo[3,4-b]Quinoline-5,8 (4H,9H)-Dione (***4v***)

Following the general procedure described above, in a 5 mL MW tube, 30 mg of embelin (0.1 mmol), 17.5 mg of heptanaldehyde (0.15 mmol, 21.4 µL) and 24.4 mg of 3-amino-5-phenylpyrazole (0.15 mmol) were dissolved in 2 mL of DCE and treated with 1.8 mg of EDDA (10 mol %). The tube was sealed, and the reaction mixture was irradiated at 150 °C for 10 min. The product was purified by Sephadex LH-20 using hex/DCM/MeOH (2:2:1) as eluent to yield 42.3 mg (78%) of **4v** as an amorphous blue solid. Mp: 172.5–173.2 °C; ^1^H-NMR (500 MHz, CDCl_3_) δ 0.73 (t, *J* = 7.2 Hz, 3H, (CH2)CH3), 0.87 (t, *J* = 6.9 Hz, 3H, H-11′’’), 0.97 (m, 7H), 1.25 (bs, 16H), 1.50 (m, 2H), 1.63 (m, 2H), 2.44 (t, *J* = 7.6 Hz, 2H), 4.64 (t, *J* = 4.2 Hz, 1H, H-4), 7.43 (t, *J* = 7.1 Hz, 1H, H-4′), 7.50 (t, *J* = 7.4 Hz, 2H, H-3′ + H-5′), 7.58 (d, *J* = 7.4 Hz, 2H, H-2′ + H-6′); ^13^C-NMR (125 MHz, CDCl_3_) δ 14.0 (CH_3_), 14.1 (CH_3_), 22.5 (CH_2_), 22.6 (CH_2_), 22.7 (CH_2_), 24.9 (CH_2_), 28.2 (CH_2_), 29.2 (CH_2_), 29.4 (CH_2_), 29.5 (CH_2_), 29.6 (CH_2_), 29.7 (CH_2_ x 2), 29.7 (CH_2_), 30.0 (CH_2_), 31.6 (CH_2_), 31.9 (CH_2_), 35.5 (CH), 103.5 (C), 107.3 (C), 115.8 (C), 126.6 (CH x 2), 129.0 (CH), 129.3 (CH x 2), 129.5 (CH), 139.4 (C), 142.5 (C), 147.9 (C), 154.1 (C), 178.8 (C), 181.41 (C); EIMS *m*/*z* (%) 531 ([M^+^], 3), 474 (5), 446 (100), 307 (7); HREIMS 531.3461 (calcd for C_33_H_45_N_3_O_3_ [M^+^] 531.3461); IR 3440 (N-H), 3298 (O-H), 2922, 2853 (C-H aliph), 1639 (C=O), 1526, 1483, 1438 (C=N, C=C), 1378 (N-N), 1268, 1232, 1205, 1147, 1120, 1072, 1014, 977, 870, 823 cm^−1^. 

### 3.24. 4-(Tert-Butyl)-6-Hydroxy-3-Phenyl-7-Undecyl-1H-Pyrazolo[3,4-b]Quinoline-5,8 (4H,9H)-Dione (***4w***)

Following the general procedure described above, in a 5 mL MW tube, 30 mg of embelin (0.1 mmol), 13.2 mg of pyvalaldehyde (0.15 mmol, 18.5 µL) and 24.4 mg of 3-amino-5-phenylpyrazole (0.15 mmol) were dissolved in 2 mL of DCE and treated with 1.8 mg of EDDA (10 mol %). The tube was sealed, and the reaction mixture was irradiated at 150 °C for 10 min. The product was purified by Sephadex LH-20 using hex/DCM/MeOH (2:2:1) as eluent to yield 24.3 mg (47%) of **4w** as an amorphous blue solid. Mp: 160.1–160.9 °C; ^1^H-NMR (500 MHz, CDCl_3_) δ 0.87 (t, *J* = 7.1 Hz, 3H, H-11′’’), 0.93 (s, 9H, -C(CH_3_)_3_), 1.25 (bs, 16H, H-4′’’-H-10′’’), 1.49 (m, 2H, H-3′’’), 2.45 (t, *J* = 7.8 Hz, 2H, H-1′’’), 5.41 (s, 1H, H-4), 6.20 (bs, 1H, OH), 7.32 (t, *J* = 7.3 Hz, 1H, H-4′), 7.40 (t, *J* = 7.7 Hz, 2H, H-3′ + H-5′), 7.80 (d, *J* = 7.3 Hz, 2H, H-2′ + H-6′); ^13^C-NMR (125 MHz, CDCl_3_) δ 14.1 (CH_3_), 22.6 (CH_2_), 22.7 (CH_2_), 27.1 (CH_3_ x 3), 27.6 (CH_2_), 28.1 (CH_2_), 29.3 (CH_2_), 29.4 (CH_2_), 29.6 (CH_2_ x 2), 29.7 (CH_2_), 31.9 (CH_2_), 40.7 (CH), 61.9 (C), 87.7 (C), 104.3 (C), 116.6 (C), 125.6 (CH x 2), 128.0 (CH), 128.6 (CH x 2), 133.2 (C), 138.0 (C), 139.6 (C) 150.9 (C), 154.0 (C), 178.9 (C), 180.9 (C); EIMS *m*/*z* (%) 446 ([M^+^-C_4_H_9_], 100), 418 (4), 307 (15), 276 (5); HRMS: 446.2423 (calcd for C_27_H_32_N_3_O_3_ [M^+^-C_4_H_9_] 446.2444); IR v_max_ 3310 (N-H), 3223 (O-H), 2955, 2916, 2850 (C-H aliph), 1635 (C=O), 1556, 1519, 1497, 1464, 1428 (C=C, C=N), 1359 (N-N), 1305, 1264, 1223, 1184, 1119, 1073, 1026, 996, 956, 916, 882, 841 cm^−1^.

### 3.25. 6-Hydroxy-7-Octyl-3-Phenyl-4-(4-(Trifluoromethyl)Phenyl)-1H-Pyrazolo[3,4-b]Quinoline-5,8 (4H,9H)-Dione (***9***)

Following the general procedure described above, in a 5 mL MW tube, 30 mg of 2,5-dihydroxy-3-octylcyclohexa-2,5-diene-1,4-dione (0.12 mmol), 31.1 mg of 4-(trifluoromethyl)-benzaldehyde (0.18 mmol, 24.4 µL) and 28.4 mg of 3-amino-5-phenylpyrazole (0.18 mmol) were dissolved in 2 mL of DCE and treated with 2.1 mg of EDDA (10 mol % m). The tube was sealed, and the reaction mixture was irradiated at 150 °C for 10 min. The product was purified by filtration and washed with n-hex to yield 28.5 mg (43%) of **9** as an amorphous blue solid. Mp: 243.7–244.4 °C; ^1^H-NMR (500 MHz, CDCl_3_) 0.85 (t, *J* = 7.1 Hz, 3H, H-8′’’), 1.25 (m, 10H, H-3′’’-H-7′’’), 1.45 (m, 2H, H-2′’’), 2.41 (t, *J* = 7.8 Hz, 2H, H-1′’’), 5.58 (s, 1H, H-4), 7.30 (m, 2H, H-3′’ + H-5′’), 7.39 (m, 4H), 7.37 (d, *J* = 8.1 Hz, 2H, H-2′’ + H-6′’); ^13^C-NMR (125 MHz, CDCl_3_) δ 14.1 (CH_3_), 22.6 (CH_2_), 22.7 (CH_2_), 28.1 (CH_2_), 29.2 (CH_2_), 29.4 (CH_2_), 29.7 (CH_2_), 31.9 (CH_2_), 36.0 (CH), 103.3 (C), 107.2 (C), 116.1 (C), 116.3 (C), 123.2 (C), 125.2 (CH x 2, *J* = 2.8 Hz), 126.7 (CH x 2), 128.3 (C), 128.4 (CH x 2), 128.2 (C, *J* = 31.2 Hz), 129.1 (CH x 2), 129.3 (CH), 140.2(C), 141.0(C), 147.3(C), 149.0(C), 154.1(C), 178.6 (C), 182.3 (C); EIMS *m*/*z* (%) 549 ([M^+^], 90), 450 (17), 404 (100), 306 (7); HREIMS 549.2214 (calcd for C_31_H_30_N_3_O_3_F_3_ [M^+^] 549.2239); IR v_max_ 3433 (N-H), 3251 (O-H), 2927, 2854 (C-H aliph), 1643 (C=O), 1570, 1504 (C=N, C=C), 1354 (N-N), 1323, 1274, 1161, 1118, 1064, 1018, 833, 690 cm^−1^.

### 3.26. 7-Hexyl-6-Hydroxy-3-Phenyl-4-(4-(Trifluoromethyl)Phenyl)-1H-Pyrazolo[3,4-b]Quinoline-5,8 (4H,9H)-Dione (***10***)

Following the general procedure described above, in a 5 mL MW tube, 30 mg of 3-hexyl-2,5-dihydroxycyclohexa-2,5-diene-1,4-dione (0.134 mmol), 35 mg of 4-(trifluoromethyl)-benzaldehyde (0.20 mmol, 27.4 µL) and 32 mg of 3-amino-5-phenylpyrazole (0.20 mmol) were dissolved in 2 mL DCE and treated with 2.4 mg of EDDA (10 mol %). The tube was sealed, and the reaction mixture was irradiated at 150 °C for 10 min. The product was purified by filtration and washed with n-hex to yield 35.6 mg (51%) of **10** as an amorphous blue solid. Mp: 239.9–240.7 °C; ^1^H-NMR (500 MHz, CDCl_3_) δ 0.86 (t, *J* = 7.2 Hz, 3H, H-6′’’), 1.29 (m, 6H, H-3′’’-H-5′’’), 1.46 (m, 2H, H-2′’’), 2.41 (t, *J* = 7.5 Hz, 2H, H-1′’’), 5.58 (s, 1H, H-4), 7.30 (m, 2H), 7.39 (m, 4H), 7.38 (m, 5H), 7.44 (d, *J* = 8.3 Hz, 2H); ^13^C-NMR (125 MHz, CDCl_3_) δ 14.1 (CH_3_), 22.6 (CH_2_ x 2), 28.0 (CH_2_), 29.3 (CH_2_), 31.6 (CH_2_), 36.0 (CH), 103.3 (C), 107.2 (C), 116.3 (C), 123.9 (C, *J* = 271.6 Hz), 125.3 (CH x 2, *J* = 3.7 Hz), 125.9 (C), 126.7 (CH x 2), 128.3 (C), 128.4 (CH x 2), 128.8 (C, *J* = 31.2 Hz), 129.1 (CH x 2), 129.3 (CH), 140.2 (C), 141.0 (C), 147.2 (C), 148.9 (C), 154.0 (C), 178.6 (C), 182.3 (C); EIMS *m*/*z* (%) 521 ([M^+^], 1); 423 (29); 359 (100); 303 (62); 301 (69); 289 (47); HREIMS 493.1601 (calcd for C_27_H_22_N_3_O_3_F_3_ [M^+^] 493.1613); IR v_max_ 3435 (N-H), 3260 (O-H), 2928, 2858 (C-H aliph), 1643 (C=O), 1569, 1504 (C=C, C=N), 1385 (N-N), 1323, 1269, 1165, 1122, 1065, 979, 833, 694 cm^−1^.

### 3.27. 7-Butyl-6-Hydroxy-3-Phenyl-4-(4-(Trifluoromethyl)Phenyl)-1H-Pyrazolo[3,4-b]Quinoline-5,8 (4H,9H)-Dione (***11***)

Following the general procedure described above, in a 5 mL MW tube, 30 mg of 3-butyl-2,5-dihydroxycyclohexa-2,5-diene-1,4-dione (0.153 mmol), 40 mg of 4-(trifluromethyl)-benzaldehyde (0.23 mmol, 31.3 µL) and 36.5 mg of 3-amino-5-phenylpyrazole (0.23 mmol) were dissolved in 2 mL DCE and treated with 2.8 mg of EDDA (10 mol %). The tube was sealed, and the reaction mixture was irradiated at 150 °C for 10 min. The product was purified by filtration and washed with n-hex to yield 49.7 mg (67%) of compound **11** as an amorphous violet solid. Mp: 257.2–258.6 °C; ^1^H-NMR (500 MHz, DMSO-d_6_) δ 0.87 (t, *J* = 7.2 Hz, 3H, H-4′’’), 1.27 (m, 2H, H-3′’’), 1.34 (m, 2H, H-2′’’), 2.27 (t, *J* = 7.5 Hz, 2H, H-1′’’), 5.63 (s, 1H, H-4), 5.76 (s, 1H, OH), 7.31 (m, 1H), 7.39 (m, 4H), 7.49 (d, *J* = 8.4 Hz, 2H, H-2′’ + H-6′’), 7.53 (d, *J* = 7.4 Hz, 2H, H-3′’ + H-5′’); ^13^C-NMR (125 MHz, CDCl_3_) δ 14.3 (CH_3_), 22.2 (CH_2_), 22.7 (CH_2_), 30.4 (CH_2_), 36.4 (CH), 107.5 (C), 116.2 (C), 123.8 (C), 125.2 (CH x 2, *J* = 3.2 Hz), 125.6 (C), 126.7 (CH x 2), 127.1 (C, *J* = 32.3 Hz), 127.4 (C), 128.7 (CH), 129.1 (CH x 2), 129.2 (CH x 2), 179.1 (C), 181.7 (C); EIMS *m*/*z* (%) 493 ([M^+^], 31), 450 (12), 348 (100), 306 (9); HREIMS 493.1601 (calcd for C_27_H_22_N_3_O_3_F_3_ [M^+^] 493.1613); IR v_max_ 3441 (N-H), 3356 (O-H), 2967, 2932, 2870 (C-H aliph), 1636 (C=O), 1566, 1527, 1493 (C=C, C=N), 1327 (N-N), 1296, 1204, 1165, 1111, 1068, 980, 930, 833, 690, 660 cm^−1^.

### 3.28. 7-Ethyl-6-Hydroxy-3-Phenyl-4-(4-(Trifluoromethyl)Phenyl)-1H-Pyrazolo[3,4-b]Quinoline-5,8 (4H,9H)-Dione (***12***)

Following the general procedure described above, in a 5 mL MW tube, 30 mg of 3-ethyl-2,5-dihydroxycyclohexa-2,5-diene-1,4-dione (0.18 mmol), 46.6 mg of 4-(trifluromethyl)-benzaldehyde (0.27 mmol, 36.5 µL) and 42.6 mg of 3-amino-5-phenylpyrazole (0.27 mmol) were dissolved in 2 mL DCE and treated with 3.2 mg of EDDA (10 mol %). The tube was sealed, and the reaction mixture was irradiated at 150 °C for 10 min. The product was purified by filtration and washed with n-hex to yield 27.9 mg (34%) of compound **12** as an amorphous blue solid. Mp: 175.2–177.0 °C; ^1^H-NMR (500 MHz, DMSO-d_6_) δ 0.95 (t, *J* = 7.2 Hz, 3H, H-2′’’), 2.28 (q, *J* = 7.2 Hz, 2H, H-1′’’), 5.60 (s, 1H, H-4), 5.76 (s, 1H, OH), 7.31 (m, 1H), 7.39 (m, 4H), 7.49 (d, *J* = 8.2 Hz, 2H, H-2′’ + H-6′’), 7.53 (d, *J* = 7.6 Hz, 2H, H-3′’ + H-5′’); ^13^C-NMR (125 MHz, CDCl_3_) δ 12.6 (CH_3_), 15.3 (CH_2_), 35.9 (CH), 101.9 (C), 107.1 (C), 117.3 (C), 124.2 (C, *J* = 271.9 Hz), 124.7 (CH, *J* = 3.4 Hz), 126.2 (CH x 2), 126.6 (C, *J* = 31.7 Hz), 127.4 (C), 128.2 (CH), 128.6 (CH x 2), 128.7 (CH x 2), 128.8 (C), 138.5 (C), 140.0 (C), 146.5 (C), 150.4 (C), 155.1 (C), 178.5 (C), 181.5 (C); EIMS *m*/*z* (%) 465 ([M^+^], 34), 321 (21), 320 (100), 292 (5); HREIMS 465.1291 (calcd for C_25_H_18_N_3_O_3_F_3_ [M^+^] 465.1300); IR v_max_ 3433 (N-H), 3275 (O-H), 2974, 2932 (C=C, C=N), 1643 (C=O), 1589, 1531, 1504 (C=C, C=N), 1346 (N-N), 1318, 1273, 1161, 1111, 1065, 1022, 976, 841, 690 cm^−1^.

### 3.29. 6-Methoxy-3-Phenyl-4-(4-(Trifluoromethyl)Phenyl)-7-Undecyl-4,9-Dihydro-1H-Pyrazolo[3,4-b]Quinoline-5,8-Dione (***13***)

To 15 mg of **4g** (0.025 mmol) dissolved in 7.5 mL of a mixture diethyl ether/MeOH (2:1) an excess of trimethylsilyldiazomethane (Me_3_SiCHN_2_, 2 equiv) was added. The reaction mixture was stirred at room temperature until disappearance of the starting material (24 h). The solvent was removed under reduced pressure and the product **13** was quantitatively obtained without further purifications (15.1 mg, 100%). Mp: 241.8–242.5 °C. ^1^H-NMR (500 MHz, DMSO-d_6_) δ 0.84 (t, *J* = 7.1 Hz, 3H, H-11′’’), 1.22 (bs, 16H, H-3′’’-H-10′’’), 1.34 (m, 2H, H-2′’’), 2.30 (t, *J* = 7.7 Hz, 2H, H-1′’’), 3.89 (s, 3H, -OCH_3_), 5.62 (s, 1H, H-4), 7.34 (m, 3H), 7.40 (t, *J* = 7.7 Hz, 2H, H-2′’-H-6′’), 7.49 (d, *J* = 8.1 Hz, 2H), 7.53 (d, *J* = 7.5 Hz, 2H, H-3′’-H-5′’); ^13^C-NMR (125 MHz, DMSO-d_6_) δ 13.9 (CH_3_), 22.0 (CH_2_), 22.3 (CH_2_), 28.0 (CH_2_), 28.6 (CH_2_), 28.7 (CH_2_), 28.8 (CH_2_), 28.9 (CH_2_ x 3), 31.2 (CH_2_), 35.7 (CH), 61.3 (CH_3_), 101.9 (C), 109.2 (C), 113.6 (C), 124.1 (C, *J*_C-F_ = 273.4 Hz), 124.9 (CH x 2, *J*_C-F_ = 3.5 Hz), 126.2 (CH x 2), 126.6 (C, *J*_C-F_ = 31.4 Hz), 127.3 (C), 128.2 (CH), 128.3 (CH x 2), 128.8 (CH x 2), 129.3 (C), 138.9 (C), 146.9 (C), 150.5 (C), 156.9 (C), 179.3 (C), 182.7 (C); EIMS *m*/*z* (%) 605 ([M^+^], 56), 474 (20), 460 (100), 432 (14); HREIMS 605.2889 (calcd for C_35_H_38_N_3_O_3_F_3_ [M^+^] 605.2865).

### 3.30. 3-(4-Chlorophenyl)-6-Hydroxy-4-(4-(Trifluoromethyl)Phenyl)-7-Undecyl-4,9-Dihydro-1H-Pyrazolo[3,4-b]Quinoline-5,8-Dione (***14a***)

Following the general procedure, in a 5 mL MW tube, 30 mg of embelin (0.1 mmol), 21.3 μL of 4-(trifluoromethyl)benzaldehyde (0.15 mmol) and 27.5 mg of 3-(4-chlorophenyl)-1*H*-pyrazol-5-amine (0.15 mmol) were dissolved in 2 mL of DCE and treated with 1.8 mg of EDDA (10 mol %). The tube was sealed, and the reaction mixture was irradiated at 150 °C for 10 min. The product was purified by filtration and washed with n-hex to yield 51.6 mg (83%) of **14a** as an amorphous violet solid. Mp: 231.6–233.0 °C; ^1^H-NMR (500 MHz, DMSO-d_6_) δ 0.84 (t, *J* = 6.8 Hz, 3H, H-11′’’), 1.21 (bs, 16H, H-3′’’-H-10′’’), 1.33 (m, 2H, H-2′’’), 2.25 (t, *J* = 7.4 Hz, 2H, H-1′’’), 5.61 (s, 1H, H-4), 7.36 (d, *J* = 8.0 Hz, 2H, H-2′’ + H-6′’), 7.45 (d, *J* = 7.1 Hz, 2H, H-2′ + H-6′), 7.48 (d, *J* = 8.0 Hz, 2H, H-3′’ + H-5′’), 7.56 (d, *J* = 8.5 Hz, 2H, H-3′ + H-5′); ^13^C-NMR (125 MHz, DMSO-d_6_) δ 14.4 (CH_3_), 22.5 (CH_2_), 22.6 (CH_2_), 28.2 (CH_2_), 29.2 (CH_2_), 29.4 (CH_2_ x 2), 29.5 (CH_2_ x 2), 29.6 (CH_2_), 31.7 (CH_2_), 36.3 (CH), 102.8 (C), 107.5 (C), 115.9 (C), 123.8 (C), 125.3 (CH x 2, *J*_C-F_ = 3.4 Hz), 125.6 (C, *J*_C-F_ = 273.4 Hz), 127.1 (C, *J*_C-F_ = 31.8 Hz), 128.5 (CH x 2), 129.1 (CH x 2), 129.2 (C); 129.3 (CH x 2), 133.3 (C), 137.8 (C), 140.9 (C), 147.0 (C), 150.9 (C), 179.5 (C), 180.7 (C); EIMS *m*/*z* (%) 624 ([M^+^], 100), 606 (15), 590 (18), 478 (11); HREIMS 624.2239 (calcd for C_34_H_34_N_3_O_3_F_3_^35^Cl [M^+^] 624.2241), 626.2224 (calcd for C_34_H_34_N_3_O_3_F_3_^37^Cl [M^+^] 626.2211).

### 3.31. 3-(4-Bromophenyl)-6-Hydroxy-4-(4-(Trifluoromethyl)Phenyl)-7-Undecyl-4,9-Dihydro-1H–Pyrazolo[3,4-b]Quinoline-5,8-Dione (***14b***)

Following the general procedure, in a 5 mL MW tube, 30 mg of embelin (0.1 mmol), 21.3 μL of 4-(trifluoromethyl)benzaldehyde (0.15 mmol) and 36.4 mg of 3-(4-bromophenyl)-1*H*-pyrazol-5-amine (0.15 mmol) were dissolved in 2 mL of DCE and treated with 1.8 mg of EDDA (10 mol %). The tube was sealed, and the reaction mixture was irradiated at 150 °C for 10 min. The product was purified by filtration and washed with n-hex to yield 58 mg (86%) of **14b** as an amorphous violet solid. Mp: 227.6–229.0 °C. ^1^H-NMR (500 MHz, DMSO-d_6_) δ 0.84 (t, *J* = 7.0 Hz, 3H, H-11′’’), 1.21 (bs, 16H, H-3′’’-H-10′’’), 1.34 (m, 2H, H-2′’), 2.26 (t, *J* = 7.7 Hz, 2H, H-1′’), 5.63 (s, 1H, H-4), 7.37 (d, *J* = 7.9 Hz, 2H), 7.48 (m, 4H), 7.59 (d, *J* = 8.6 Hz, 2H); ^13^C-NMR (125 MHz, DMSO-d_6_) δ 14.4 (CH_3_), 22.4 (CH_2_), 22.5 (CH_2_), 28.1 (CH_2_), 29.2 (CH_2_), 29.3 (CH_2_), 29.4 (CH_2_ x 2), 29.5 (CH_2_ x 2), 31.7 (CH_2_), 36.3 (CH), 102.9 (C), 107.6 (C), 116.5 (C), 122.8 (C, *J*_C-F_ = 271.6 Hz), 125.2 (CH x 2, *J*_C-F_ = 3.6 Hz), 125.5 (C), 126.7 (C), 127.2 (C, *J*_C-F_ = 30.5 Hz), 128.7 (CH x 2), 129.1 (CH x 2), 132.2 (CH x 2), 137.7 (C), 140.5 (C), 147.2 (C), 150.8 (C), 156.2 (C), 179.0 (C), 182.0 (C).

### 3.32. 3-(4-Fluorophenyl)-6-Hydroxy-4-(4-(Trifluoromethyl)Phenyl)-7-Undecyl-4,9-Dihydro-1H-Pyrazolo[3,4-b]Quinoline-5,8-Dione (***14c***)

Following the general procedure, in a 5 mL MW tube, 30 mg of embelin (0.1 mmol), 21.3 μL of 4-(trifluoromethyl)benzaldehyde (0.15 mmol) and 27.1 mg of 3-(4-fluorophenyl)-1*H*-pyrazol-5-amine (0.15 mmol) were dissolved in 2 mL of DCE and treated with 1.8 mg of EDDA (10 mol %). The tube was sealed, and the reaction mixture was irradiated at 150 °C for 10 min. The product was purified by filtration and washed with n-Hex to yield 52.1 mg (85%) of **14c** as an amorphous violet solid. Mp: 228.2–229.8 °C; ^1^H-NMR (500 MHz, DMSO-d_6_) δ 0.83 (t, *J* = 7.2 Hz, 3H, H-11′’’), 1.21 (bs, 16H, H-3′’’-H-10′’’), 1.34 (m, 2H, H-2′’’), 2.26 (t, *J* = 7.8 Hz, 2H, H-1′’’), 5.62 (s, 1H, H-4), 7.23 (t, *J* = 8.4 Hz, 2H, H-3′’ + H-5′’), 7.35 (d, *J* = 7.8 Hz, 2H, H-3′ + H-5′), 7.48 (d, *J* = 7.8 Hz, 2H, H-2′ + H-6′), 7.57 (m, 2H, H-2′’ + H-6′’); ^13^C-NMR (125 MHz, DMSO-d_6_) δ 14.3 (CH_3_), 22.4 (CH_2_), 22.5 (CH_2_), 28.1 (CH_2_), 29.1 (CH_2_), 29.2 (CH_2_), 29.3 (CH_2_), 29.4 (CH_2_), 29.5 (CH_2_ x 2), 31.7 (CH_2_), 36.3 (CH), 102.5 (C), 107.5 (C), 116.2 (CH x 2, *J*_C-F_ = 21.1 Hz), 116.5 (C), 125.3 (CH x 2, *J*_C-F_ = 2.2 Hz), 127.3 (C), 129.0 (CH x 4), 129.1 (C), 140.5 (C), 142.5 (C), 143.8 (C), 150.9 (C), 156.1 (C), 161.5 (C), 163.1 (C), 178.9 (C), 182.1 (C); EIMS *m*/*z* (%) 609 ([M^+^], 93), 668 (56), 464 (100), 323 (39). HREIMS 609.2632 (calcd for C_34_H_35_N_3_O_3_F_4_ [M^+^] 609.2615).

### 3.33. 3-(3-Fluorophenyl)-6-Hydroxy-4-(4-(Trifluoromethyl)Phenyl)-7-Undecyl-4,9-Dihydro-1H-Pyrazolo[3,4-b]Quinoline-5,8-Dione (***14d***)

Following the general procedure, in a 5 mL MW tube, 30 mg of embelin (0.1 mmol), 21.3 μL of 4-(trifluoromethyl)benzaldehyde (0.15 mmol) and 27.1 mg of 3-(3-fluorophenyl)-1*H*-pyrazol-5-amine (0.15 mmol) were dissolved in 2 mL of DCE and treated with 1.8 mg of EDDA (10 mol %). The tube was sealed and the reaction mixture was irradiated at 150 °C for 10 min. The product was purified by filtration and washed with n-hex to yield 46.6 mg (76%) of **14d** as an amorphous violet solid. Mp: 213.8–215.0 °C. ^1^H-NMR (500 MHz, DMSO-d_6_) δ 0.83 (t, *J* = 7.1 Hz, 3H, H-11′’’), 1.21 (bs, 16H, H-3′’’-H-10′’’), 1.31 (m, 2H, H-2′’’), 2.23 (t, *J* = 7.7 Hz, 2H, H-1′’’), 5.61 (s, 1H, H-4), 7.14 (td, *J* = 2.3, 8.6 Hz, 1H), 7.33 (dt, *J* = 2.2, 10.7 Hz, 1H), 7.36 (m, 3H), 7.43 (m, 1H), 7.50 (d, *J* = 8.4 Hz, 2H, H-3′’ + H-5′’); ^13^C-NMR (125 MHz, DMSO-d_6_) δ 13.8 (CH_3_), 21.0 (CH_2_), 22.0 (CH_2_), 27.8 (CH_2_), 28.6 (CH_2_), 28.8 (CH_2_), 28.9 (CH_2_ x 2), 29.0 (CH_2_ x 2), 31.2 (CH_2_), 35.8 (CH), 102.4 (C), 106.9 (C), 112.8 (CH, *J*_C-F_ = 22.6 Hz), 114.9 (CH, *J*_C-F_ = 21.5 Hz), 118.8 (C, *J*_C-F_ = 14.8 Hz), 115.1 (C), 122.4 (CH x 2, *J*_C-F_ = 2.6 Hz), 124.1 (C, *J*_C-F_ = 272.3 Hz), 124.8 (CH, *J*_C-F_ = 4.4 Hz), 126.6 (C, *J*_C-F_ = 31.3 Hz), 128.6 (CH x 2), 129.0 (C), 130.8 (CH, *J*_C-F_ = 8.5 Hz), 140.5 (C), 150.4 (C), 161.1 (C), 163.0 (C), 171.9 (C), 179.3 (C), 179.7 (C); EIMS *m*/*z* (%) 609 ([M^+^], 100), 468 (38), 464 (96), 324 (19); HREIMS 609.2596 (calcd for C_34_H_35_N_3_O_3_F_4_ [M^+^] 609.2615).

### 3.34. 6-Hydroxy-3-(4-Methoxyphenyl)-4-(4-(Trifluoromethyl)Phenyl)-7-Undecyl-4,9-Dihydro-1H-Pyrazolo[3,4-b]Quinoline-5,8-Dione (***14e***)

Following the general procedure, in a 5 mL MW tube, 30 mg of embelin (0.1 mmol), 21.3 μL of 4-(trifluoromethyl)benzaldehyde (0.15 mmol) and 28.9 mg of 3-(4-methoxyphenyl)-1*H*-pyrazol-5-amine (0.15 mmol) were dissolved in 2 mL of DCE and treated with 1.8 mg of EDDA (10 mol %). The tube was sealed and the reaction mixture was irradiated at 150 °C for 10 min. The product was purified by filtration and washed with n-hex to yield 58.6 mg (94%) of **14e** as an amorphous violet solid. Mp: 206.6–207.5 °C; ^1^H-NMR (500 MHz, DMSO-d_6_) δ 0.84 (t, *J* = 7.5 Hz, 3H, H-11′’’), 1.21 (bs, 16H, H-3′’’-H-10′’’), 1.33 (m, 2H, H-2′’’), 2.25 (t, *J* = 7.6 Hz, 2H, H-1′’’), 3.75 (s, 3H, OCH_3_), 5.57 (s, 1H, H-4), 6.95 (d, *J* = 8.5 Hz, 2H, H-3′ + H-5′), 7.36 (d, *J* = 8.0 Hz, 2H, H-2′’ + H-6′’), 7.46 (d, *J* = 8.9 Hz, 2H, H-3′’ + H-5′’), 7.50 (d, *J* = 8.5 Hz, 2H, H-2′ + H-6′); ^13^C-NMR (125 MHz, DMSO-d_6_) δ 14.4 (CH_3_), 22.5 (CH_2_ x 2), 28.2 (CH_2_), 29.2 (CH_2_), 29.3 (CH_2_), 29.4 (CH_2_ x 2), 29.5 (CH_2_ x 2), 31.7 (CH_2_), 36.3 (CH), 55.6 (CH_3_), 101.7 (C), 107.5 (C), 114.7 (CH x 2), 116.0 (C), 121.8 (C), 124.7 (C, *J*_C-F_ = 272.9 Hz), 125.3 (CH x 2, *J*_C-F_ = 3.2 Hz), 126.5 (C), 127.1 (C, *J*_C-F_ = 31.5 Hz), 128.1 (CH x 2), 129.0 (CH x 2), 139.1 (C); 140.8 (C), 147.0 (C), 151.1 (C), 159.6 (C), 179.2 (C), 181.3 (C); EIMS *m*/*z* (%) 621 ([M^+^], 65), 480 (21), 476 (100), 345 (30); HREIMS 621.2825 (calcd for C_35_H_38_N_3_O_4_F_3_ [M^+^] 621.2814).

### 3.35. 3-(4-(Dimethylamino)Phenyl)-6-Hydroxy-4-(4-(Trifluoromethyl)Phenyl)-7-Undecyl-4,9-Dihydro-1H-Pyrazolo[3,4-b]Quinoline-5,8-Dione (***14f***)

Following the general procedure, in a 5 mL MW tube, 30 mg of embelin (0.1 mmol), 21.3 μL of 4-(trifluoromethyl)benzaldehyde (0.15 mmol) and 30.9 mg of 3-(4-(dimethylamino)phenyl)-1*H*-pyrazol-5-amine (0.15 mmol) were dissolved in 2 mL of DCE and treated with 1.8 mg of EDDA (10 mol %). The tube was sealed, and the reaction mixture was irradiated at 150 °C for 10 min. The product was purified by filtration and washed with n-hex to yield 56.9 mg (90%) of **14f** as an amorphous violet solid. Mp: 242.4–244.0 °C; ^1^H-NMR (500 MHz, DMSO-d_6_) δ 0.84 (t, *J* = 7.5 Hz, 3H, H-11′’’), 1.21 (bs, 16H, H-3′’’-H-10′’’), 1.33 (m, 2H, H-2′’’), 2.25 (t, *J* = 7.6 Hz, 2H, H-1′’’), 3.75 (s, 6H, -N(CH_3_)_2_), 5.57 (s, 1H, H-4), 6.95 (d, *J* = 8.5 Hz, 2H, H-3′ + H-5′), 7.36 (d, *J* = 8.0 Hz, 2H, H-2′’ + H-6′’), 7.46 (d, *J* = 8.9 Hz, 2H, H-3′’ + H-5′’), 7.50 (d, *J* = 8.5 Hz, 2H, H-2′ + H-6′); ^13^C-NMR (125 MHz, DMSO-d_6_) δ 13.9 (CH_3_), 21.9 (CH_2_), 22.0 (CH_2_), 27.6 (CH_2_), 28.6 (CH_2_), 28.8 (CH_2_), 28.9 (CH_2_ x 2), 29.0 (CH_2_ x 2), 31.2 (CH_2_), 35.9 (CH), 40.2 (CH_3_ x 2), 100.2 (C), 107.1 (C), 118.8 (CH x 2), 115.8 (C), 116.3 (C), 124.2 (C, *J*_C-F_ = 274.9 Hz), 124.8 (CH x 2, *J*_C-F_ = 3.7 Hz), 126.6 (C, *J*_C-F_ = 30.7 Hz), 126.9 (CH x 2), 128.6 (CH x 2), 138.9 (C), 140.0 (C), 146.6 (C), 149.9 (C), 150.7 (C), 156.1 (C), 178.3 (C), 181.5 (C); EIMS *m*/*z* (%) 634 ([M^+^], 83), 493 (16), 489 (100), 347 (10); HREIMS 634.3113 (calcd for C_36_H_41_N_4_O_3_F_3_ [M^+^] 634.3131).

### 3.36. 3-(Furan-2-yl)-6-Hydroxy-4-(4-(Trifluoromethyl)Phenyl)-7-Undecyl-4,9-Dihydro-1H-Pyrazolo[3,4-b]Quinoline-5,8-Dione (***14g***)

Following the general procedure, in a 5 mL MW tube, 30 mg of embelin (0.1 mmol), 21.3 μL of 4-(trifluoromethyl)benzaldehyde (0.15 mmol) and 22.8 mg of 3-(furan-2-yl)-1*H*-pyrazol-5-amine (0.15 mmol) were dissolved in 2 mL of DCE and treated with 1.8 mg of EDDA (10 mol %). The tube was sealed, and the reaction mixture was irradiated at 150 °C for 10 min. The product was purified by filtration and washed with n-hex to yield 48.8 mg (82%) of **14g** as an amorphous violet solid. Mp: 165.0–166.8 °C. ^1^H-NMR (500 MHz, DMSO-d_6_) δ 0.83 (t, *J* = 6.2 Hz, 3H, H-11′’’), 1.21 (bs, 16H, H-3′’’-H-10′’’), 1.33 (m, 2H, H-2′’’), 2.26 (t, *J* = 7.3 Hz, 2H, H-1′’’), 5.49 (s, 1H, H-4), 6.53 (bs, 1H, H-4′), 6.63 (bs, 1H, H-5′), 7.46 (d, *J* = 7.3 Hz, 2H, H-2′’ + H-6′’), 7.54 (d, *J* = 7.6 Hz, 2H, H-3′’ + H-5′’), 7.74 (s, 1H, H-3′); ^13^C-NMR (125 MHz, DMSO-d_6_) δ 13.9 (CH_3_), 22.0 (CH_2_ x 2), 27.6 (CH_2_), 28.6 (CH_2_), 28.8 (CH_2_), 28.9 (CH_2_ x 2), 29.0 (CH_2_ x 2), 31.2 (CH_2_), 35.7 (CH), 101.5 (C), 107.1 (C), 107.6 (CH), 111.7 (CH), 115.7 (C), 124.1 (C, *J*_C-F_ = 272.6 Hz), 124.7 (CH x 2, *J*_C-F_ = 3.6 Hz), 126.6 (C, *J*_C-F_ = 31.5 Hz), 128.7 (CH x 2), 140.3 (C), 143.1 (CH), 143.8 (C), 146.2 (C), 150.0 (C), 150.8 (C); 156.9 (C), 178.9 (C), 180.6 (C); EIMS *m*/*z* (%) 581 ([M^+^], 89), 439 (44), 435 (100), 295 (23); HREIMS 581.2491 (calcd for C_32_H_34_N_3_O_4_F_3_ [M^+^] 581.2501).

### 3.37. 6-Hydroxy-3-Methyl-4-(4-(Trifluoromethyl-6-Hydroxy-3-Methyl-4-(4-(Trifluoromethyl) Phenyl)-7-Undecyl-4,9-Dihydro-1H-Pyrazolo[3,4-b]Quinoline-5,8-Dione (***14h***)

Following the general procedure, in a 5 mL MW tube, 30 mg of embelin (0.1 mmol), 21.3 μL of 4-(trifluoromethyl)benzaldehyde (0.15 mmol) and 14.9 mg of 3-methyl-1*H*-pyrazol-5-amine (0.15 mmol) were dissolved in 2 mL of DCE and treated with 1.8 mg of EDDA (10 mol %). The tube was sealed, and the reaction mixture was irradiated at 150 °C for 10 min. The product was purified by filtration and washed with n-hex to yield 41.4 mg (78%) of **14h** as an amorphous violet solid. Mp: 249.8–250.9 °C. ^1^H-NMR (500 MHz, DMSO-d_6_) δ 0.83 (t, *J* = 6.6 Hz, 3H, H-11′’’), 1.20 (bs, 16H, H-3′’’-H-10′’’), 1.33 (m, 2H, H-2′’’), 1.88 (s, 3H, CH_3_), 2.25 (t, *J* = 6.8 Hz, 2H, H-1′’’), 5.24 (s, 1H, H-4), 7.43 (d, *J* = 7.6 Hz, 2H, H-2′’ + H-6′’), 7.58 (d, *J* = 7.6 Hz, 2H, H-3′’ + H-5′’); ^13^C-NMR (125 MHz, DMSO-d_6_) δ 9.4 (CH_3_), 13.9 (CH_3_), 21.9 (CH_2_), 22.0 (CH_2_), 27.6 (CH_2_), 28.7 (CH_2_), 28.8 (CH_2_), 28.9 (CH_2_ x 2), 29.0 (CH_2_ x 2), 31.2 (CH_2_), 35.6 (CH), 102.7 (C), 106.5 (C), 115.8 (C), 124.2 (C, *J*_C-F_ = 272.9 Hz), 124.9 (CH x 2, *J*_C-F_ = 3.3 Hz), 126.5 (C, *J*_C-F_ = 31.8 Hz), 128.3 (CH x 2), 135.9 (C), 140.8 (C), 144.9 (C), 151.3 (C), 155.9 (C); 178.6 (C), 181.6 (C); EIMS *m*/*z* (%) 529 ([M^+^], 100), 388 (47), 384 (86), 244 (22); HREIMS 529.2531 (calcd for C_29_H_34_N_3_O_3_F_3_ [M^+^] 529.2552).

### 3.38. 6-Hydroxy-3-Phenyl-4-(4-(Trifluoromethyl)Phenyl)-7-Undecyl-1H-Pyrazolo[3,4-b]Quinoline-5,8-Dione (***15***)

To 15 mg of compound **4g** in 3 mL of DCM 9.6 mg of DDQ (1 equiv) was added at room temperature. The reaction mixture was stirred until the disappearance of the starting material, then it was washed with a solution of saturated NaHCO_3_ and extracted with DCM. The organic layers were dried over anhydrous MgSO_4_ and filtered. The solvent was removed under reduced pressure to yield 15.4 mg (82%) of compound **15** as an amorphous orange oil. ^1^H-NMR (500 MHz, CDCl_3_) δ 0.86 (t, *J* = 7.1 Hz, 3H, H-11′’’), 1.23 (bs, 16H, H-3′’’-H-10′’’), 1.39 (m, 2H, H-2′’’), 2.73 (t, *J* = 7.8 Hz, 2H, H-1′’’), 6.97 (s, *J* = 7.1 Hz, 2H), 7.05 (t, *J* = 7.8 Hz, 2H), 7.19 (m, 3H), 7.42 (d, *J* = 8.1 Hz, 2H), 7.52 (s, 1H); ^13^C-NMR (125 MHz, CDCl_3_) δ 14.1 (CH_3_), 22.7 (CH_2_), 23.6 (CH_2_), 28.1 (CH_2_), 29.3 (CH_2_), 29.4 (CH_2_), 29.6 (CH_2_ x 2), 29.7 (CH_2_), 29.8 (CH_2_), 31.9 (CH_2_), 115.4 (C), 117.0 (C), 123.7 (C, *J*_C-F_ = 272.3 Hz), 124.6 (CH x 2, *J*_C-F_ = 3.6 Hz), 125.5 (C), 127.7 (CH x 2), 128.1 (CH), 128.7 (CH x 2), 129.1 (CH x 2), 130.7 (C, *J*_C-F_ = 32.6 Hz), 131.7 (C), 138.5 (C), 148.1 (C), 149.2 (C), 149.3 (C), 152.6 (C), 154.6 (C), 180.3 (C), 183.4 (C); EIMS *m*/*z* (%) 589 ([M^+^], 87), 561 (32), 461 (54), 449 (88); HREIMS 589.2537 (calcd for C_34_H_34_N_3_O_3_F_3_ [M^+^] 589.2552).

### 3.39. Cells

Cell lines were purchased from the American Type Culture Collection (ATCC). The cell lines were growth at 37 °C under 5% CO_2_ under humidified atmosphere. The human hematologic cell lines K562 (derived from patients during the blast crisis phase of chronic myelogenous leukemia), HEL (erythroleukemia), HL60 (acute myeloid leukemia), and the human breast cancer cells BT-549 (triple negative breast cancer) and MCF7 (ER+) were grown in RPMI-1640 medium. The triple-negative breast cancer cells MDA-MB-231 and HS-578T were grown in DMEM medium. The HER+ breast cancer cells SKBR3 were maintained in McCoy′s 5A medium. The primate non-malignant kidney Vero cells were grown in DMEM low glucose medium. Cell culture media were supplemented with 10% FBS, L-glutamine (2 mM) and PEST (50 units/mL penicillin, 50 μg/mL streptomycin).

### 3.40. Cell Viability Assay

The effects of compounds on cell viability were examined in hematological and breast cancer cells and in primate non-tumor kidney Vero cells seeded at exponential growth (5000–10,000 cells per well) in 96-well plates (BD Falcon, France). Cells were treated with vehicle (0.05% DMSO) or test compounds (0.01 to 10 µM) for 48 h. Then, mitochondrial metabolization of the MTT was used as indicator of cell viability [30]. Briefly, the tetrazolium salt 3-(4,5-methyltiazol-2yl-)-2,5diphenyl-tetrazolium bromide (MTT) (Applichen, Germany) was added to cells and incubated for 2–4 h at 37 °C, cells were lysed in 10% SDS and optical density was measured at 595 nm with the iMark Microplate Reader (BioRad).

### 3.41. ADME Property Predictions of Dihydro-1H-Pyrazolo[1,3-b] Pyridine Embelin Derivatives

The physicochemical parameters and ADME descriptors were predicted using QikProp program version 6.3 (Schrödinger, New York, NY, USA, 2020) [31] in fast mode and based on the method of Jorgensen [32,33]. Preparation of compounds and the 2D-to-3D conversion was performed using LigPrep tool, a module of the Small-Molecule Drug Discovery Suite in the Schrödinger software package, followed by MacroModel v12.3 (Schrödinger, LLC, New York, NY, USA, 2020). A conformational search was implemented using Molecular Mechanics, followed by the minimization of the energy of each conformer. The global minimum energy conformer of each compound was used as input for the ADME studies.

### 3.42. Protein Preparation and Docking

The X-ray coordinates of human protein kinase CK2 alpha subunit in complex with the inhibitor CX-4945 (PDB 3PE1). The PDB structures were prepared for docking using the Protein Preparation Workflow (Schrodinger, New York, NY, USA, 2018) accessible from within the Maestro program (Maestro, version 11.6; Schrodinger, New York, NY, USA, 2018). The substrate and water molecules were removed beyond 5 Å, bond corrections were applied to the co-crystallized ligands and an exhaustive sampling of the orientations of groups was performed. Finally, the receptors were optimized in Maestro 11.6 by using OPLS3 force field before docking study. In the final stage, the optimization and minimization on the ligand–protein complexes were carried out with the OPLS3 force field and the default value for rmsd of 0.30 Å for non-hydrogen atoms were used. The receptor grids were generated using the prepared proteins, with the docking grids centered on the center of the bound ligand for each receptor. A receptor grid was generated using a 1.00 van der Waals (vdW) radius scaling factor and 0.25 partial charge cutoff. The binding sites were enclosed in a grid box of 20 Å^3^ with default parameters and without constrains. The three-dimensional structures of the ligands to be docked were generated and prepared using LigPrep, as implemented in Maestro 11.6 (LigPrep, Schrodinger, New York, NY, USA, 2018), to generate the most probable ionization states at pH 7 ± 1 (retain original ionization state). These conformations were used as the initial input structures for the docking. In this stage a series of treatments are applied to the structures. Finally, the geometries are optimized using OPLS3 force field. These conformations were used as the initial input structures for the docking. The ligands were docked using the extra precision mode (XP) [34] without using any constraints and a 0.80 van der Waals (vdW) radius scaling factor and 0.15 partial charge cutoff. The dockings were carried out with flexibility of the residues of the pocket near to the ligand. The generated ligand poses were evaluated with empirical scoring function, GlideScore a modified version of ChemScore [35], GlideScore implemented in Glide, was used to estimate binding affinity and rank ligands [36]. The XP Pose Rank was used to select the best-docked pose for each ligand. The best correlation with the human protein kinase CK2 alpha subunit was achieved when the PDB 3PE1 was used.

## 4. Conclusions

A new family of dihydro-1*H*-pyrazolo[1,3-*b*]pyridine embelin derivatives were efficiently synthesized from a modular approach that includes Knoevenagel condensation/Michael addition/ intramolecular cyclization and dehydratation. The influence on the antiproliferative activity of structural variations was investigated. Thus, for the eight tumoral cell lines tested (HEL, K-562, HL-60, SKBR3, MCF-7, MDA-MB- 231, BT-549, and HS-578T) the presence of the free hydroxyl group at the benzoquinone nucleus, and the dihydropyridine ring were key. The best results were obtained for a C-11 long chain. Regarding the nature of the substituents at the dihydropyran ring, phenyl substituents with halogens, or -NO_2_ or -CF_3_ groups at position 4 yielded good IC_50_ values, while the modifications carried out in the pyrazol moiety revealed that 4-F-Ph, 3-F-Ph, 4-OCH_3_-Ph and 2-furyl resulted be the best substituents for the antiproliferative activity. Furthermore, the QikProp module of Schrödinger software was used as a computational method for analyzing the pharmacokinetic descriptors of the compounds with the best antiproliferative activities (**4a**, **4c**, **4e**, **4g**, **14c**, **14d**, **14e** and **14g**). The findings from this study are paving the way for further investigations concerning to obtain more selective and active compounds.

## Figures and Tables

**Table 1:** Optimization of the synthesis of **4a**.

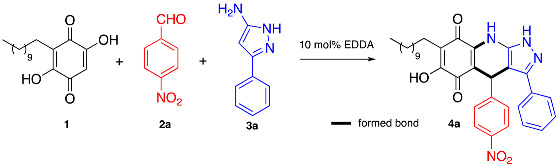			
Entry	1/2a/3a	Reaction Conditions	Yield (%)
1	1.0/1.0/1.0	EtOH, rt, 20 h	33
2	1.0/1.0/1.0	EtOH, reflux, 2 h	75
3	1.0/1.0/1.0	EtOH, MW, 130 °C, 5 min	56
4	1.0/1.0/1.0	EtOH, MW, 150 °C, 10 min	69
5	1.0/1.5/1.5	EtOH, MW, 150 °C, 15 min	76
6	1.0/1.0/1.0	DCE, MW, 150 °C, 10 min	57
7	1.0/1.5/1.5	DCE, MW, 150 °C, 10 min	94

**Table 2:** Synthesis of dihydro-1*H*-pyrazolo[1,3-*b*]pyridine embelin derivatives (**4a**–**4w**) ^a^.

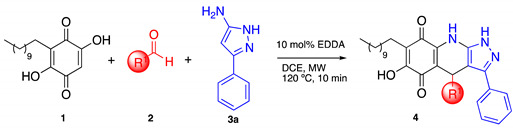			
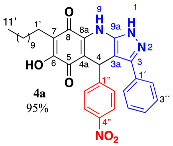	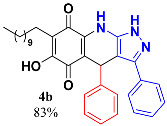	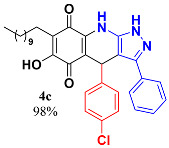	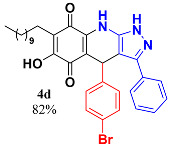
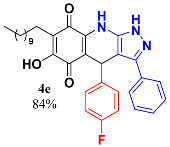	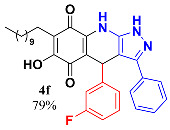	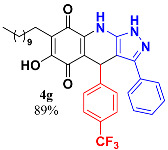	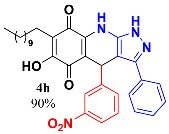
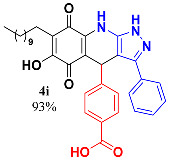	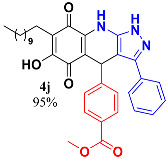	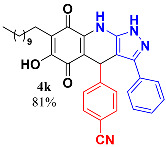	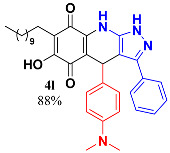
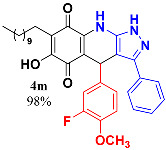	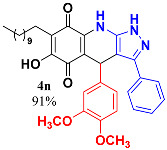	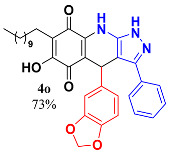	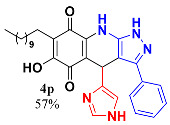
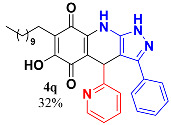	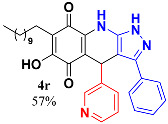	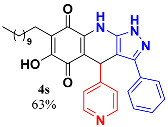	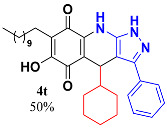
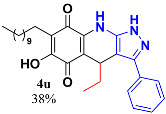	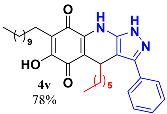	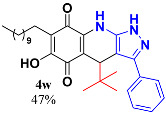	

^a^ Isolated yields.

**Table 3:** Cytotoxic activity of derivatives **4a**–**4w** in human tumor cell lines (leukemia and breast) and in primate non-tumor kidney Vero cells ^a^.

		Leukemia			Breast		Vero
Compounds	HL60	HEL	K-562	BT549	MCF7	SKBR3	
**4a**	0.70 ± 0.14	1.05 ± 0.35	1.25 ± 0.35	3.50 ± 1.27	3.25 ± 1.91	1.85 ± 0.21	2.03 ± 0.95
**4b**	1.95 ± 0.49	2.55 ± 0.07	ND	6.95 ± 0.49	>10	3.50 ± 0.28	1.88 ± 1.58
**4c**	0.90 ± 0.14	2.10 ± 0.42	2.05 ± 0.64	3.30 ± 0.14	>10	>10	20.71 ± 6.07
**4d**	1.80 ± 0.14	2.10 ± 0.28	2.50 ± 0.14	3.40 ± 0.14	>10	2.55 ± 0.78	>25
**4e**	1.00 ± 0.00	1.80 ± 0.28	3.30 ± 0.42	3.25 ± 0.92	>10	2.20 ± 0.28	2.77 ± 0.94
**4f**	>10	>10	>10	5.00 ± 0.14	>10	>10	>25
**4g**	1.75 ± 0.21	1.00 ± 0.42	2.55 ± 0.78	3.55 ± 0.64	>10	>10	>25
**4h**	>10	>10	>10	>10	>10	>10	>25
**4i**	>10	>10	>10	>10	>10	>10	n.d.
**4j**	>10	>10	6.69 ± 1.65	>10	>10	>10	>25
**4k**	>10	>10	0.92 ± 0.32	4.80 ± 0.85	>10	2.20 ± 0.57	>25
**4l**	>10	>10	>10	>10	>10	3.55 ± 0.78	>25
**4m**	2.85 ± 0.07	3.20 ± 0.00	5.50 ± 1.84	>10	>10	>10	2.03 ± 1.23
**4n**	3.15 ± 0.4	5.50 ± 0.99	>10	>10	>10	>10	1.97 ± 0.83
**4o**	1.85 ± 0.21	2.60 ± 0.42	3.40 ± 0.14	7.50 ± 0.57	>10	>10	2.97 ± 0.23
**4p**	>10	>10	>10	>10	>10	>10	>25
**4q**	>10	>10	>10	>10	>10	>10	>25
**4r**	>10	>10	6.61 ± 1.32	>10	>10	5.55 ± 1.06	>25
**4s**	>10	5.40 ± 0.81	5.75 ± 2.33	>10	>10	6.00 ± 1.98	>25
**4t**	>10	1.30 ± 0.28	4.15 ± 0.64	>10	>10	>10	>25
**4u**	6.50 ± 0.99	2.45 ± 0.07	3.60 ± 1.27	>10	4.75 ± 1.20	>10	12.14 ± 7.16
**4v**	>10	5.75 ± 0.4	>10	>10	>10	>10	24.89 ± 0.16
**4w**	>10	2.70 ± 0.42	3.30 ± 1.27	>10	>10	>10	9.87 ± 2.74

^a^ Expressed as IC_50_ values given in μM and determined as means ± SD (*n* = 3); n.d.: not determined.

**Table 4:** Preparation of derivatives **9**–**12**.

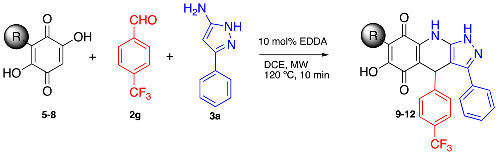			
Entry	Compounds	R^2^	Yields (%)
1	**4a**	-(CH_2_)_10_CH_3_	89
2	**9**	-(CH_2_)_7_CH_3_	43
3	**10**	-(CH_2_)_5_CH_3_	51
4	**11**	-(CH_2_)_3_CH_3_	67
5	**12**	-CH_2_CH_3_	34

**Table 5:** Cytotoxic activity of derivatives **9**–**12** in human tumor cell lines (leukemia and breast) and in primate non-tumor kidney Vero cells ^a^.

		Leukemia			Breast		Vero
Compounds	HL60	HEL	K562	BT549	MCF7	SKBR3	
**4g**	1.75 ± 0.21	1.00 ± 0.42	2.55 ± 0.78	3.55 ± 0.64	>10	>10	>25
**9**	3.99 ± 0.12	>10	>10	8.85 ± 0.4	9.10 ± 0.57	>10	>25
**10**	2.15 ± 0.36	2.13 ± 0.81	2.30 ± 0.28	9.60 ± 0.42	>10	1.50 ± 1.27	12.32 ± 0.11
**11**	2.70 ± 1.70	1.70 ± 0.14	4.87 ± 0.01	7.80 ± 1.13	>10	3.25 ± 0.07	8.42 ± 1.34
**12**	>10	>10	>10	>10	>10	>10	n.d.

^a^ Expressed as IC_50_ values given in μM and determined as means ± SD (*n* = 3).

**Table 6:** Synthesis of dihydro-1*H*-pyrazolo[3,4-*b*]pyridine embelin derivatives (**14a**–**14h**).

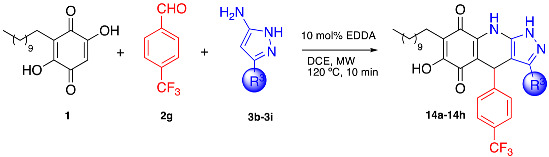		
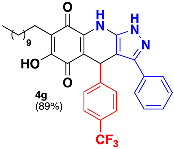	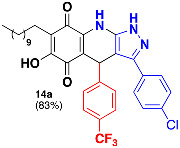	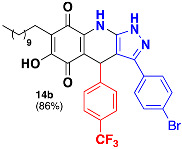
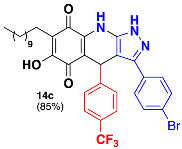	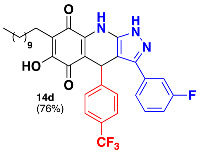	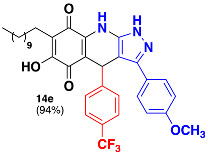
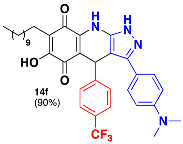	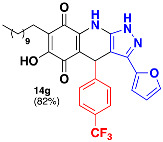	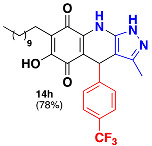

**Table 7:** Cytotoxic activity of derivatives **14a**–**14h** in human tumor cell lines (leukemia and breast) and in primate non-tumor kidney Vero cells ^a^.

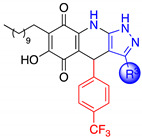							
	Leukemia			Breast			Vero
Compounds	HL60	HEL	K-562	BT549	MCF7	SKBR3	
**4g** (R^3^ = Ph)	1.75 ± 0.21	1.00 ± 0.42	2.55 ± 0.78	3.55 ± 0.64	>10	>10	>25
**14a** (R^3^ = 4-Cl-Ph)	4.20 ± 0.85	4.20 ± 1.27	3.65 ± 0.21	ND	1.52 ± 0.54	5.70 ± 0.99	10.81 ± 1.26
**14b** (R^3^ = 4-Br-Ph)	5.00 ± 0.14	5.45 ± 1.48	5.45 ± 0.78	9.55 ± 0.07	2.14 ± 0.08	7.25 ± 2.33	>25
**14c** (R^3^ = 4-CF_3_-Ph)	1.95 ± 0.07	2.75 ± 0.07	2.10 ± 0.57	4.60 ± 2.83	0.59 ± 0.00	2.30 ± 0.71	10.29 ± 2.21
**14d** (R^3^ = 3-F-Ph)	1.05 ± 0.64	2.15 ± 0.92	0.95 ± 0.4	>10	0.96 ± 0.19	2.01 ± 1.12	11.44 ± 1.25
**14e** (R^3^ = 4-OMe-Ph)	1.10 ± 0.14	1.95 ± 0.07	2.95 ± 0.4	4.4 ± 1.34	0.85 ± 0.03	2.40 ± 0.28	>25
**14f** (R^3^ = 4-(Me_2_N)-Ph	4.00 ± 0.99	5.60 ± 2.12	>10	ND	3.15 ± 2.15	8.00 ± 2.40	24.66 ± 0.49
**14g** (R^3^ = 2-furyl)	1.10 ± 0.04	2.25 ± 0.78	2.30 ± 0.57	6.90 ± 2.50	0.93 ± 0.74	2.30 ± 0.42	13.69 ± 8.56
**14h** (R^3^ = CH_3_)	3.90 ± 0.57	3.90 ± 0.28	4.4 ± 1.48	>10	1.83 ± 0.62	5.25 ± 0.64	3.34 ± 0.54

^a^ Expressed as IC_50_ values given in μM and determined as means ± SD (*n* = 3). ND: not determined.

**Table 8:** In silico ADME profile of selected dihydro-1H-pyrazolo[1,3-*b*]pyridine embelin derivatives and their range/recommended values ^a^.

Parameters	4a	4c	4e	4g	14c	14d	14e	14g	Range
QPlogBB	−3.032	−1.911	−1.950	−1.749	−0.969	−1.588	−1.984	−1.754	−3.0 to 1.2
QPPCaco	38.55	239.42	239.44	277.23	597.75	292.95	221.64	241.44	<25 poor, >500 great
QPPMDCK	14.66	260.05	190.41	535.73	2215.28	1014.70	416.00	463.99	<25 poor, >500 great
QPlogKhsa	1.238	1.408	1.335	1.531	1.362	1.559	1.533	1.334	−1.5 to 1.5
QPlogPo/w	5.322	6.461	6.204	6.951	6.842	7.153	6.948	6.404	−2.0 to 6.5
QPlogKp	−4.111	−2.634	−2.600	−2.568	−2.141	−2.680	−2.869	−2.833	−8.0 to −1.0
QPlogS	−7.845	−8.786	−8.412	−9.387	−7.715	−9.562	−9.62	−8.677	−6.5 to 0.5
#metab	4	3	3	3	3	3	4	4	1 to 8
%HOA	60.57	81.44	79.94	85.45	90.78	87.06	83.69	81.17	>80% high <25% poor
PSA	155.13	112.89	112.89	112.92	104.39	109.72	119.23	118.75	7.0 to 200.0
SASA	907.62	915.18	900.09	938.12	831.33	936.73	975.24	907.29	300.0 to 1000.0
Mol MW	568.67	558.12	541.66	591.67	609.66	609.66	621.70	581.63	130.0 to 725.0
#rotor	12	11	11	11	11	11	12	11	0 to 15
donorHB	3	3	3	3	3	3	3	3	0.0 to 6.0
accptHB	7.25	6.25	6.25	6.25	6.25	6.25	7.0	6.75	2.0 to 20.0
volume	1730.5	1712.0	1683.9	1758.7	1678.6	1768.12	1830.1	1717.0	500.0 to 2000.0
#rtvFG	0	0	0	0	0	0	0	0	0–2

^a^ Recommended values: QPlogBB (predicted brain/blood partition coefficient), QPPCaco (predicted human epithelial colorectal adenocarcinoma cell line permeability in nm/s), QPPMDCK (predicted Madin–Darby canine kidney permeability in nm/s), QPlogKhsa (predicted binding to human serum albumin), QPlogPo/w (predicted octanol/water partition coefficient), QPlogKp (skin permeability), QPlogS (predicted aqueous solubility), #metab (number of likely metabolic reactions), % HOA (predicted human oral absorption on 0 to 100%), PSA (van der Waals surface area of polar nitrogen and oxygen atoms and carbonyl atoms), SASA (total solvent accessible surface area), MW (molecular weight), #rotor (number of non-trivial, non-hindered rotatable bonds), donorHB (number of hydrogen-bond donor), accptHB (number of hydrogen-bond acceptor), #rtvFG number of reactive functional groups.

## Data Availability

Data is contained within the article or Supplementary Materials.

## Supplementary Materials

The following are available online at https://www.mdpi.com/article/10.3390/ph14101026/s1, ^1^HNMR and ^13^CNMR spectra of compounds **4a**–**4w**, **6**–**9** and **14a**–**14h**.
