# Supplementary material for: Modular Synthesis and Antiproliferative Activity of New Dihydro-1H-pyrazolo[1,3-b]pyridine Embelin Derivatives
Source: Pharmaceuticals (Basel). 2021 Oct 8;14(10):1026. doi: 10.3390/ph14101026 (PMC8541493; doi:10.3390/ph14101026)

SUPPLEMENTARY MATERIALS

Modular synthesis and antiproliferative activity of new dihydro-1*H* -pyrazolo[1,3-*b*]pyridine embelin derivatives

Pedro Martín-Acosta [1], Ángel Amesty [1], Miguel Guerra-Rodríguez [2], Borja Guerra [2], Leandro Fernández-Pérez [2,]* and Ana Estévez-Braun [1,]*

[1]*Instituto Universitario de Bio-Orgánica Antonio González, Departamento de Química Orgánica, Universidad de La Laguna, Avda. Astrofísico Francisco Sánchez Nº 2, 38206, La Laguna, Tenerife, Spain*

[2]*Instituto Universitario de Investigaciones Biomédicas y Sanitarias (IUIBS), Farmacología Molecular y Traslacional (BIOPharm), Universidad de Las Palmas de Gran Canaria (ULPGC), Las Palmas de Gran Canaria, Spain.*

^{1}HNMR and ^{13}CNMR spectra of compounds	**4a-4w**	S2-S23
^{1}HNMR and ^{13}CNMR spectra of compounds	**6-9**	S24-S27
^{1}HNMR and ^{13}CNMR spectra of compounds	**14a-14h**	S28-S35
HSQC and HMBC of compounds **4b**, **4n**, **4p**, **4s**, **4u**, **4w**		S36-S39

^{1}H-NMR ($CDCl_3$, 500 MHz) of compound 4a

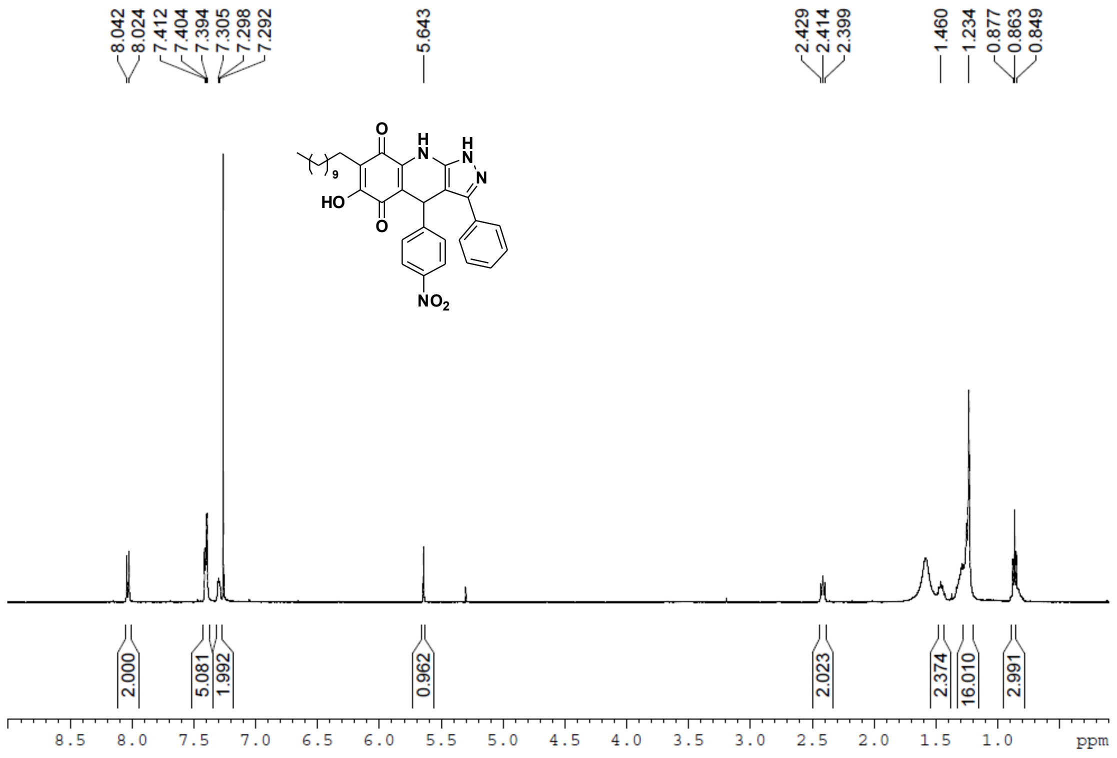

^{13}C-NMR ($CDCl_3$, 125 MHz) of compound 4a

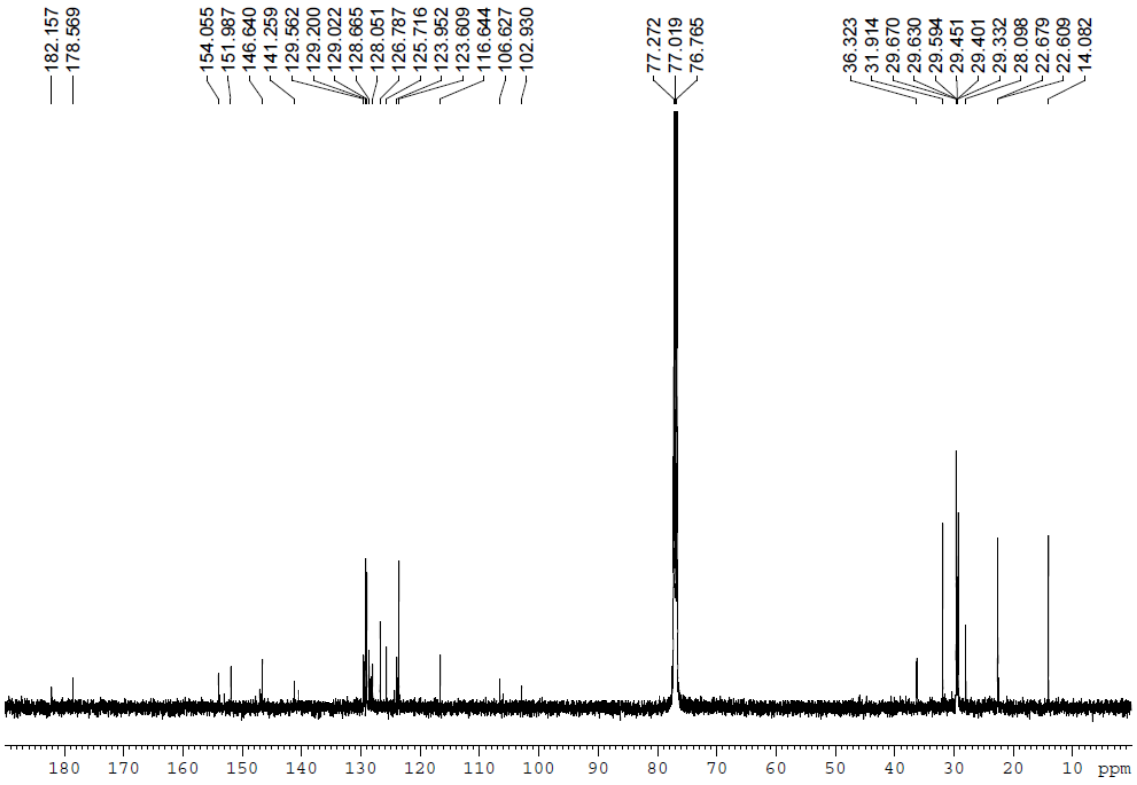

^{1}H-NMR ($CDCl_3$, 500 MHz) of compound 4b

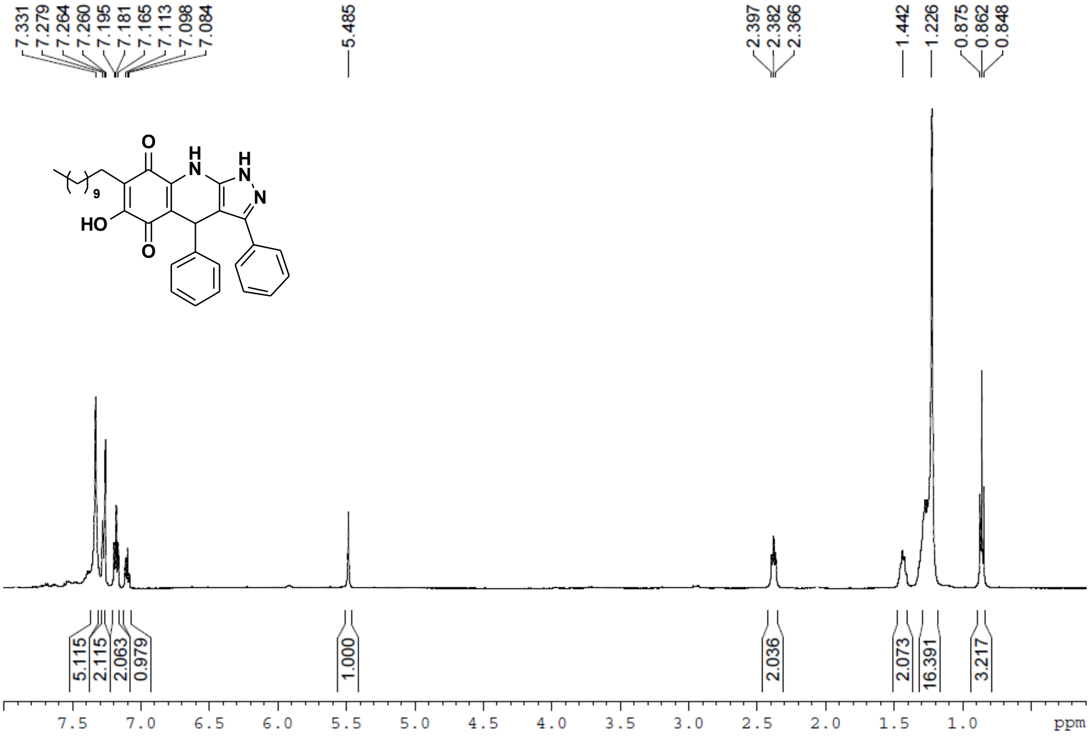

^{13}C-NMR ($CDCl_3$, 125 MHz) of compound 4b

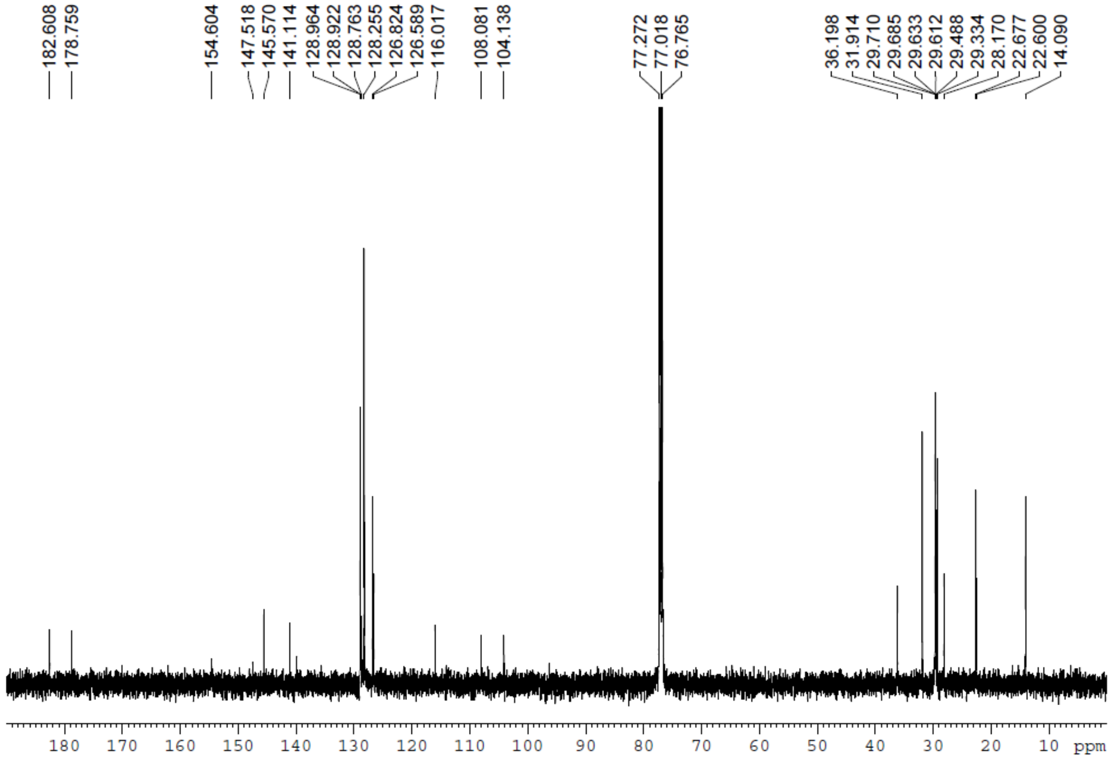

^{1}H-NMR ($CDCl_3$, 500 MHz) of compound 4c

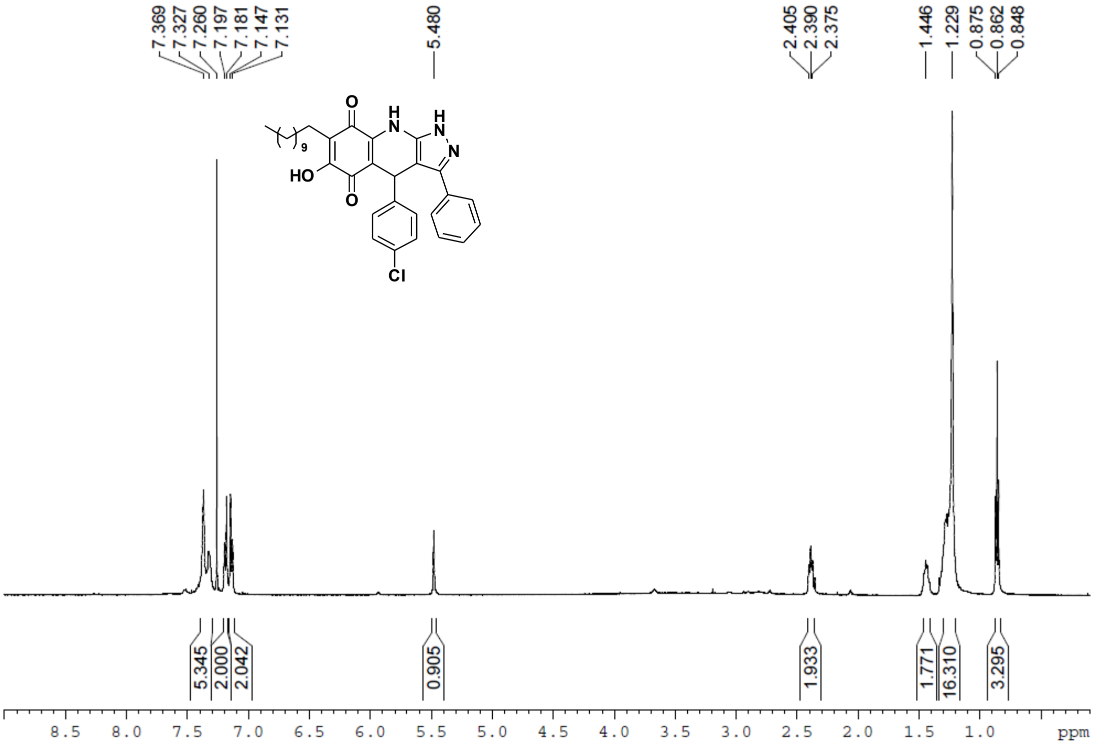

^{13}C-NMR ($CDCl_3$, 125 MHz) of compound 4c

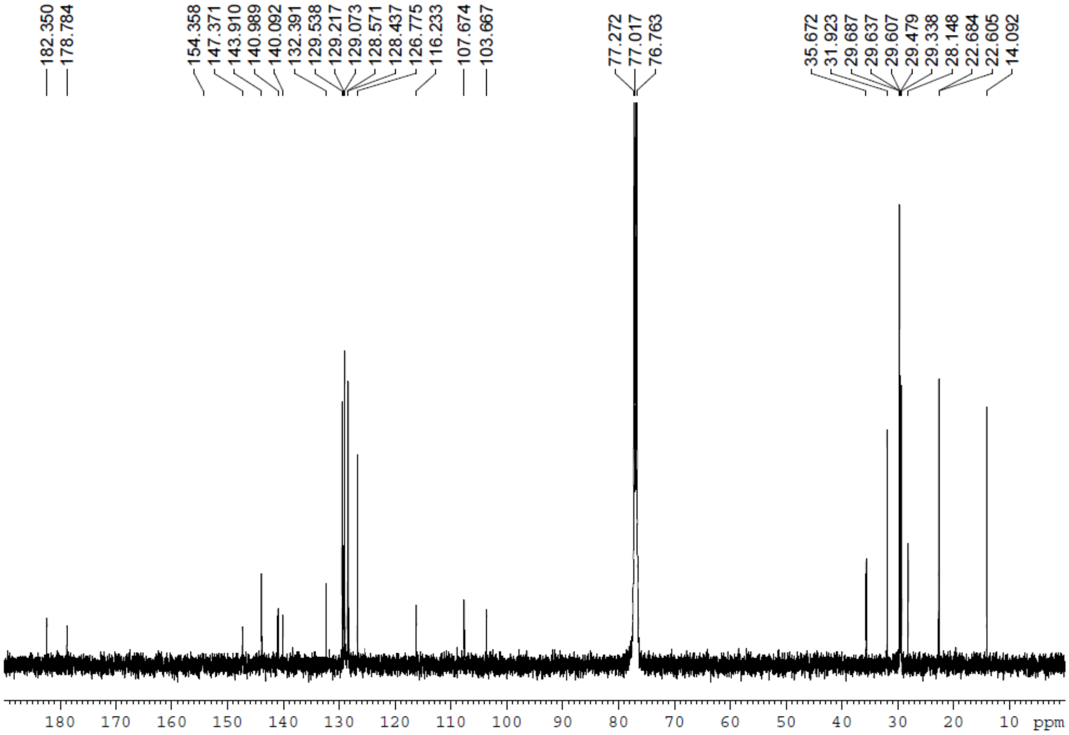

^{1}H-NMR ($CDCl_3$, 500 MHz) of compound 4d

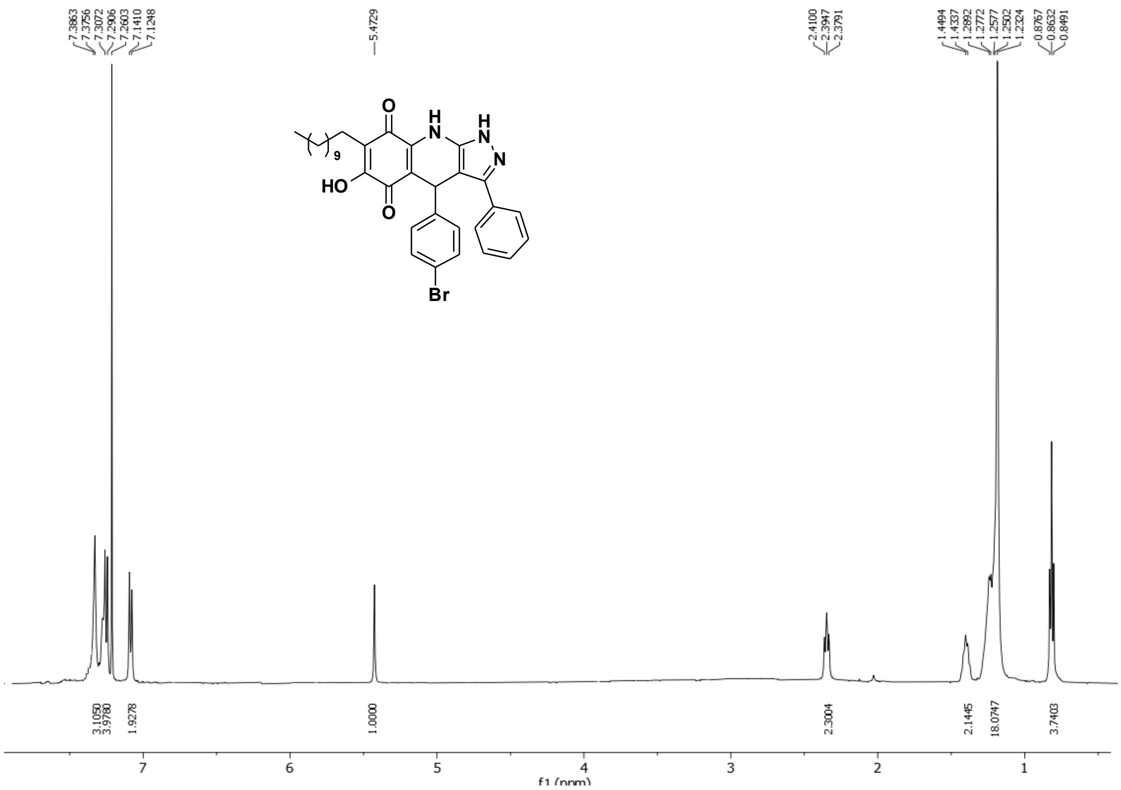

^{13}C-NMR ($CDCl_3$, 125 MHz) of compound 4d

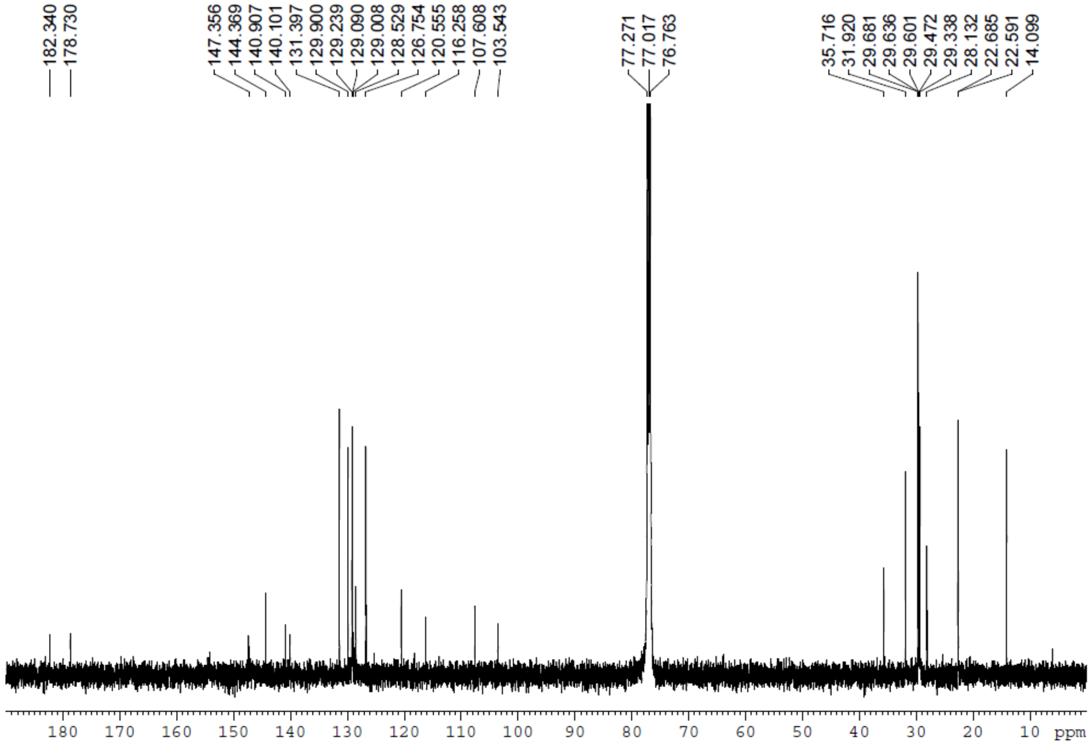

^{1}H-NMR ($CDCl_3$, 500 MHz) of compound 4e

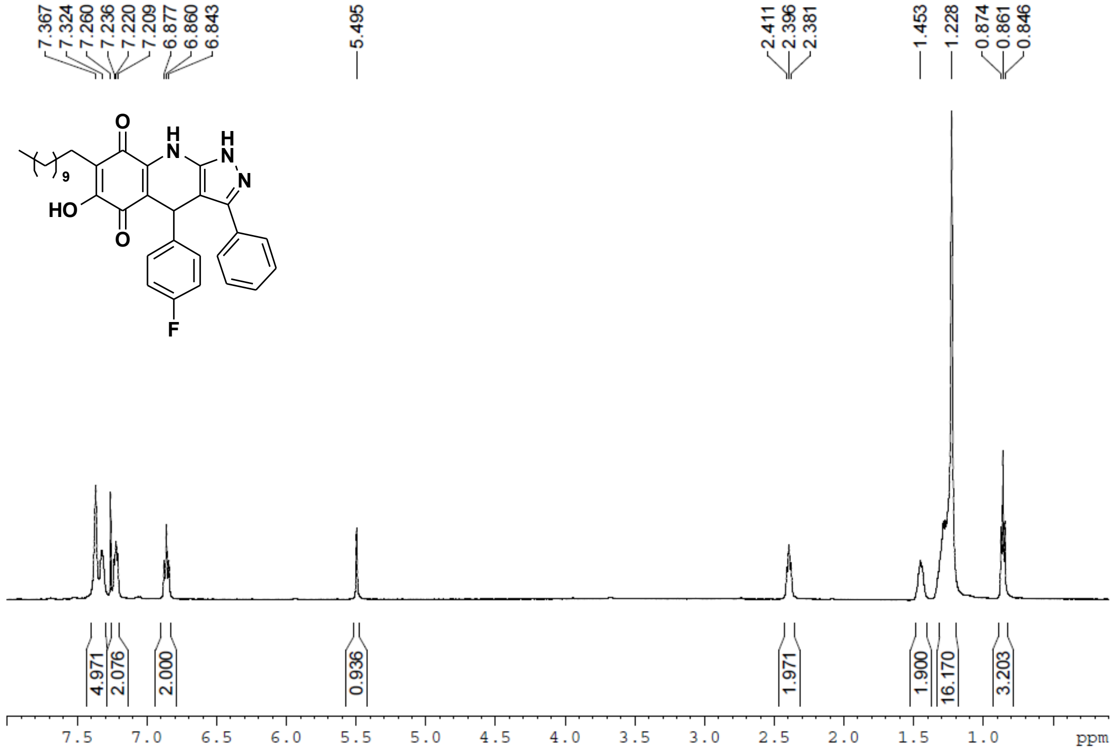

^{13}C-NMR ($CDCl_3$, 125 MHz) of compound 4e

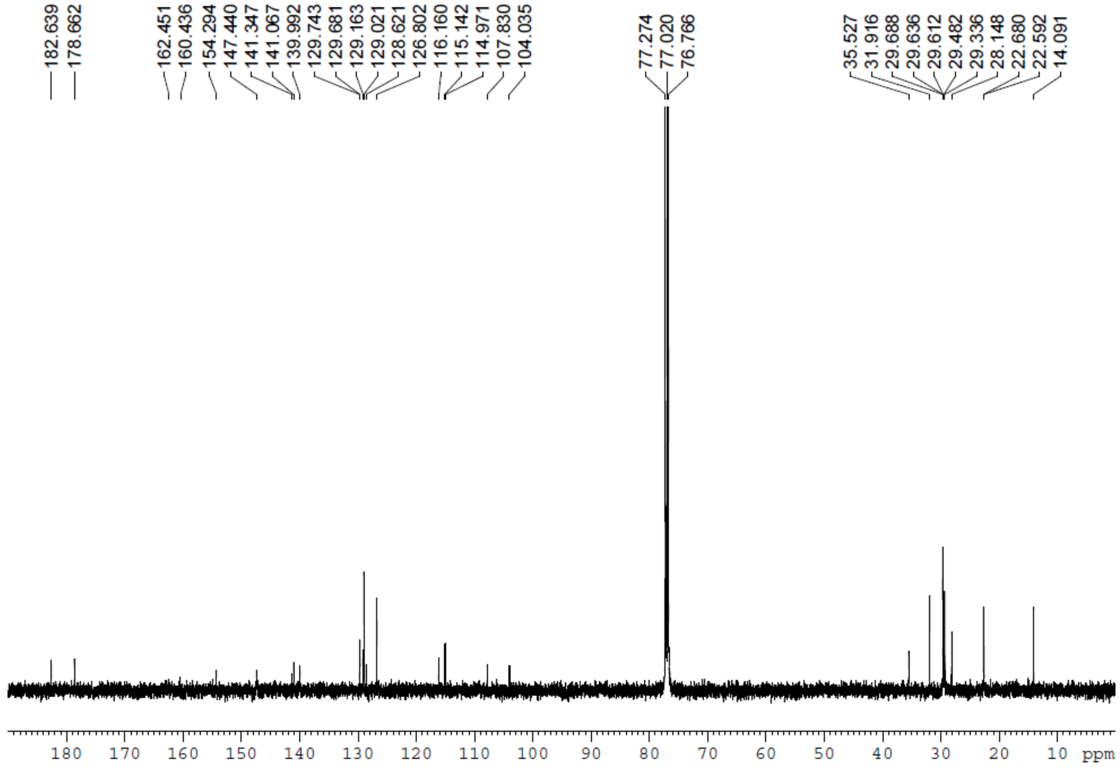

^{1}H-NMR ($CDCl_3$, 500 MHz) of compound 4f

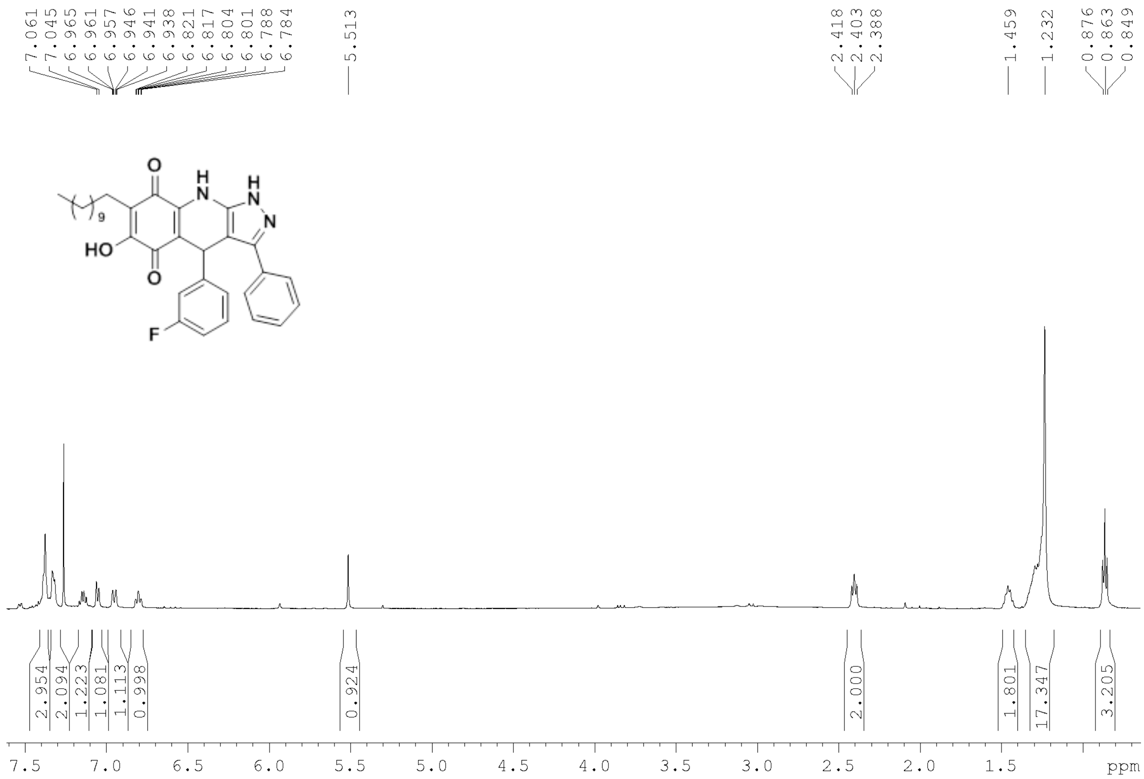

^{13}C-NMR ($CDCl_3$, 125 MHz) of compound 4f

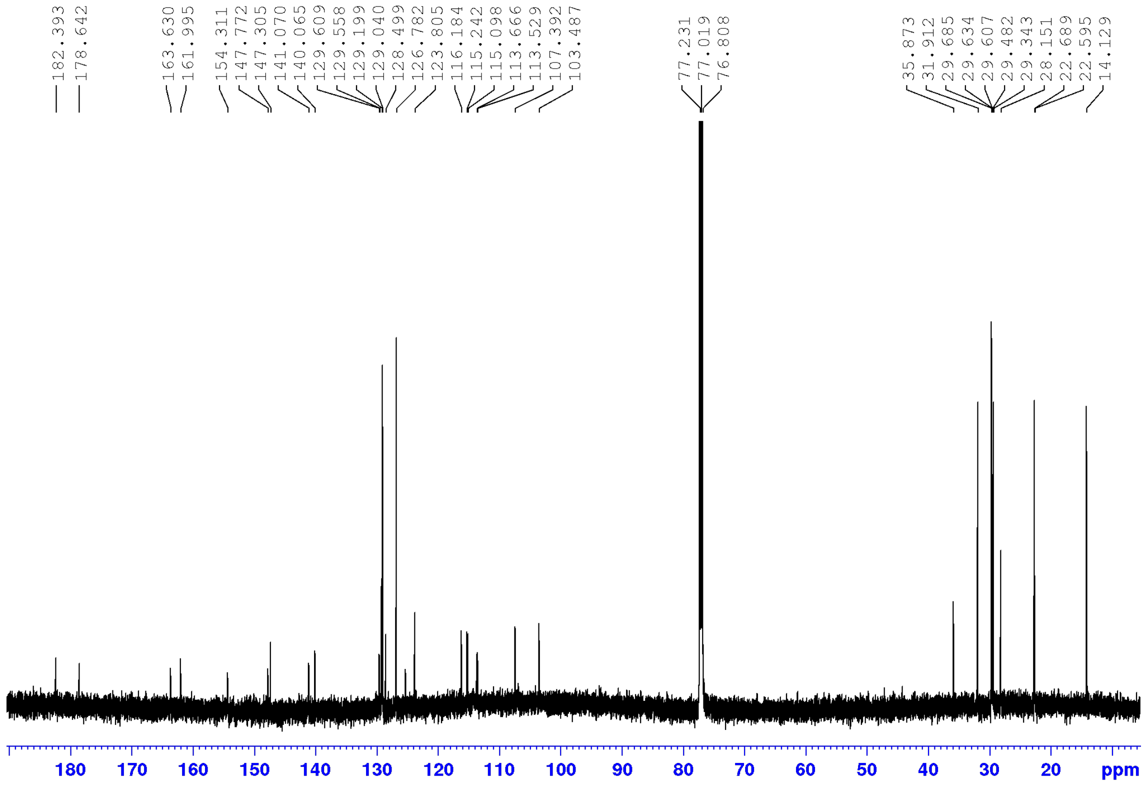

^{1}H-NMR ($CDCl_3$, 500 MHz) of compound 4g

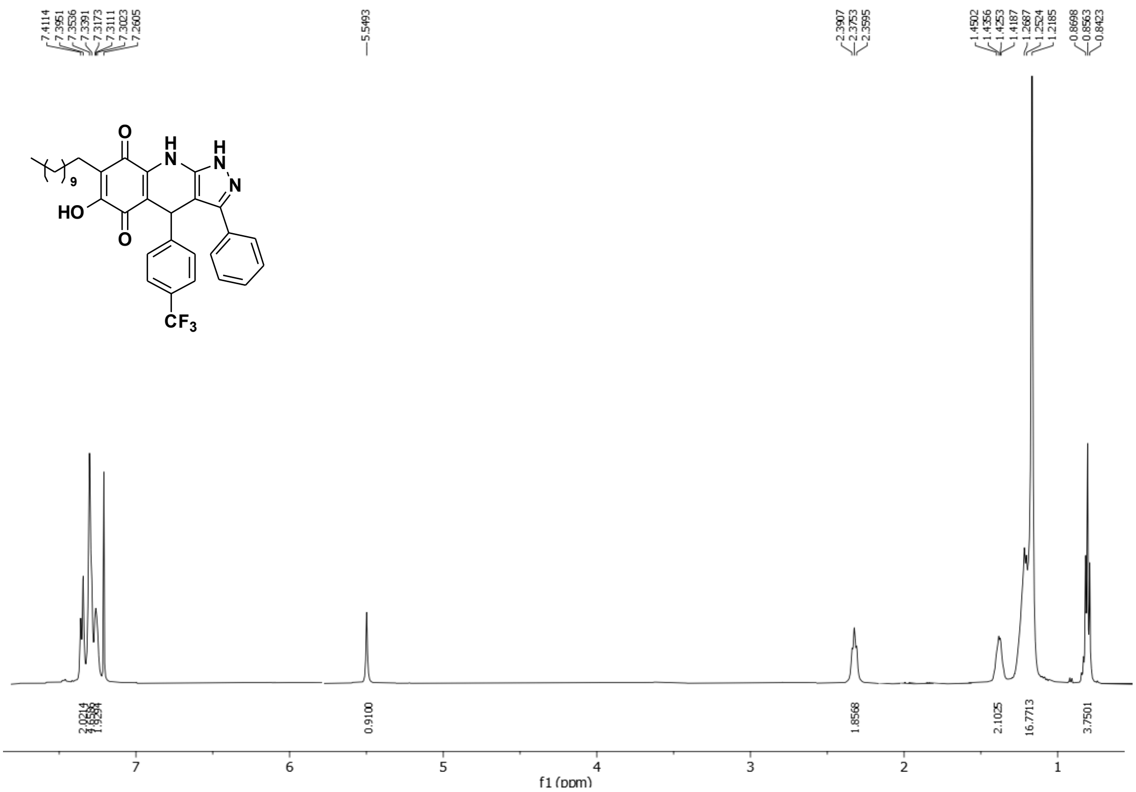

^{13}C-NMR ($CDCl_3$, 125 MHz) of compound 4g

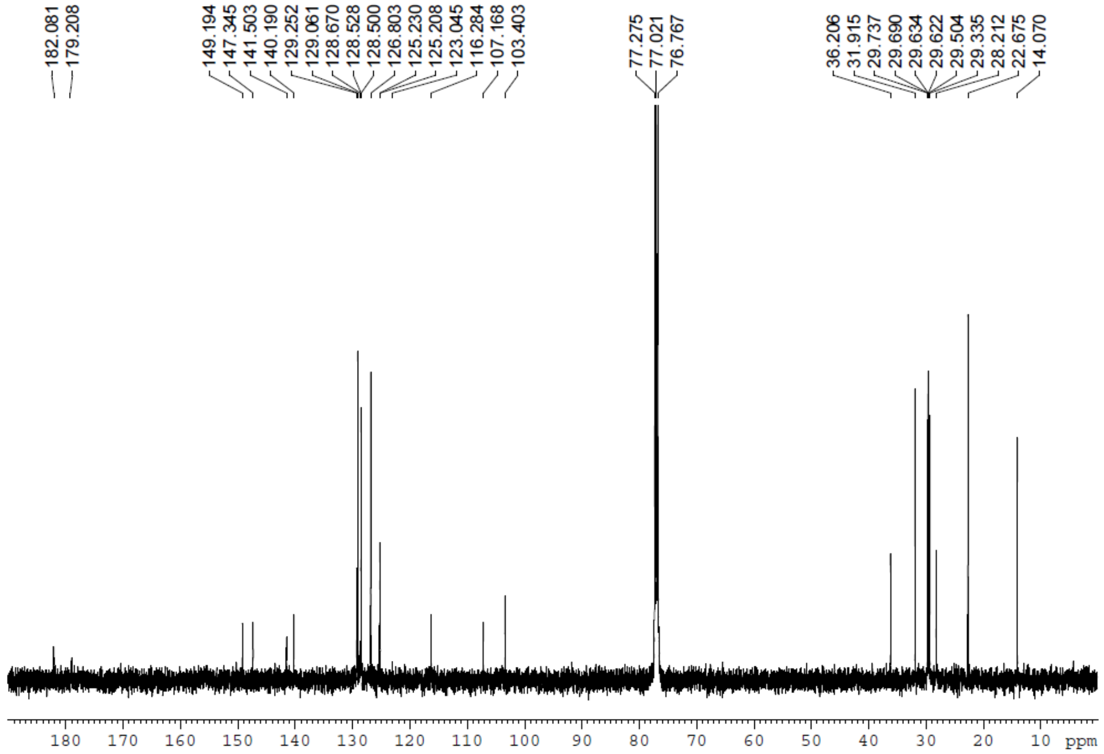

^{1}H-NMR ($CDCl_3$, 500 MHz) of compound 4h

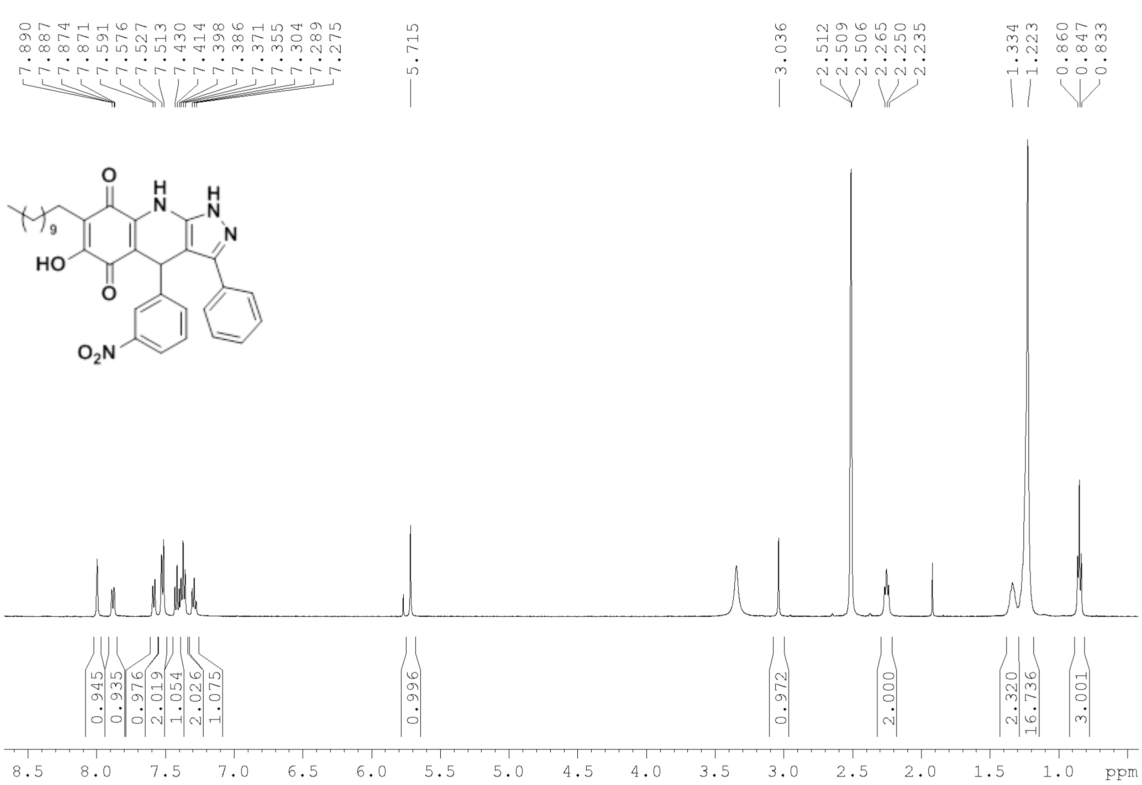

^{13}C-NMR ($CDCl_3$, 125 MHz) of compound 4h

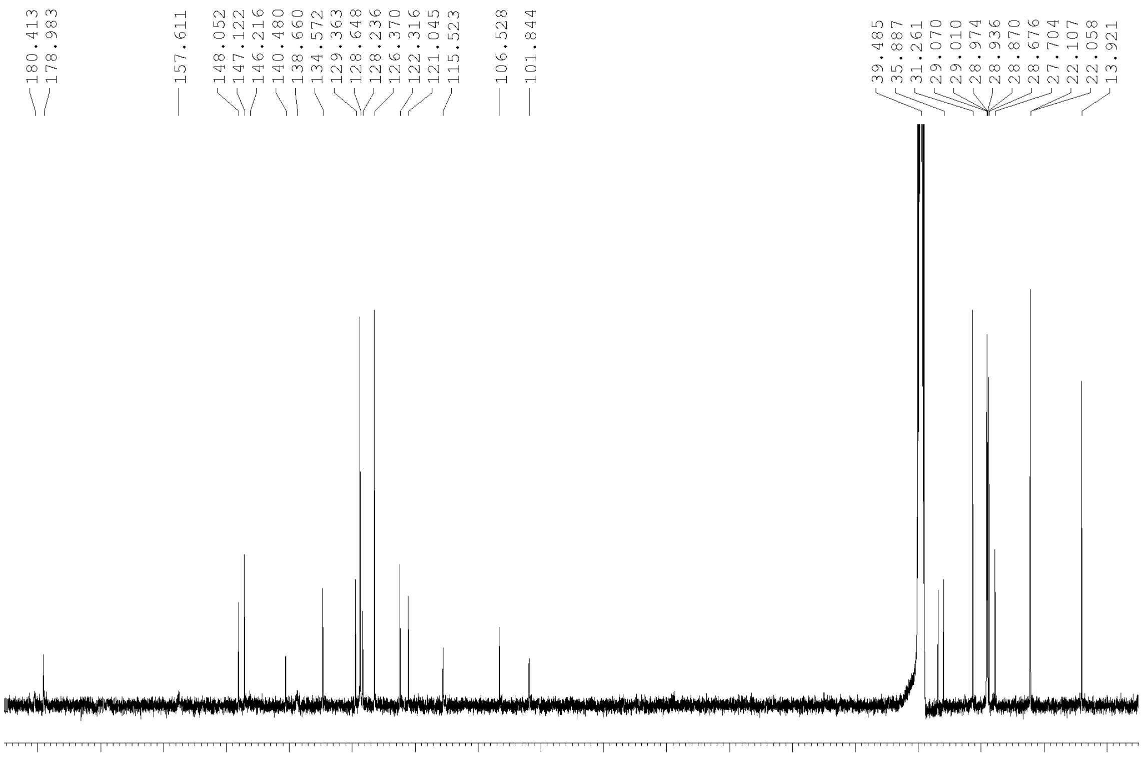

^{1}H-NMR (DMSO-d$_6$, 500 MHz) of compound 4i

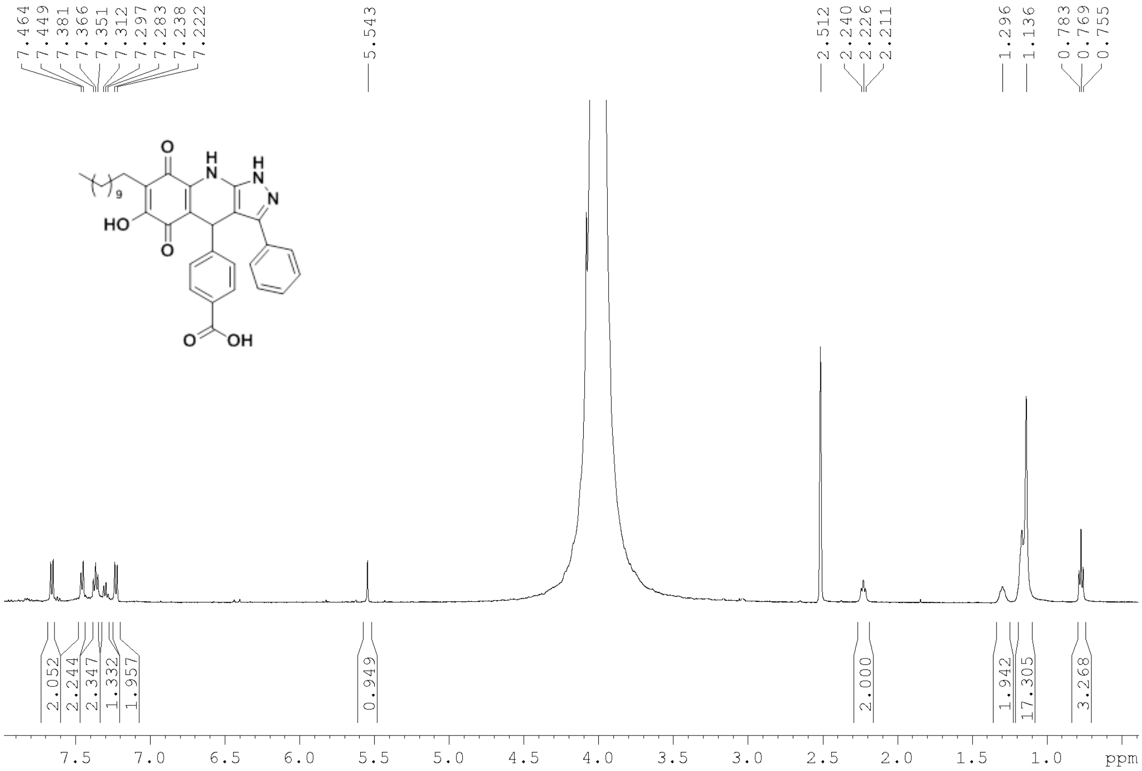

^{13}C-NMR (DMSO-d$_6$, 125 MHz) of compound 4i

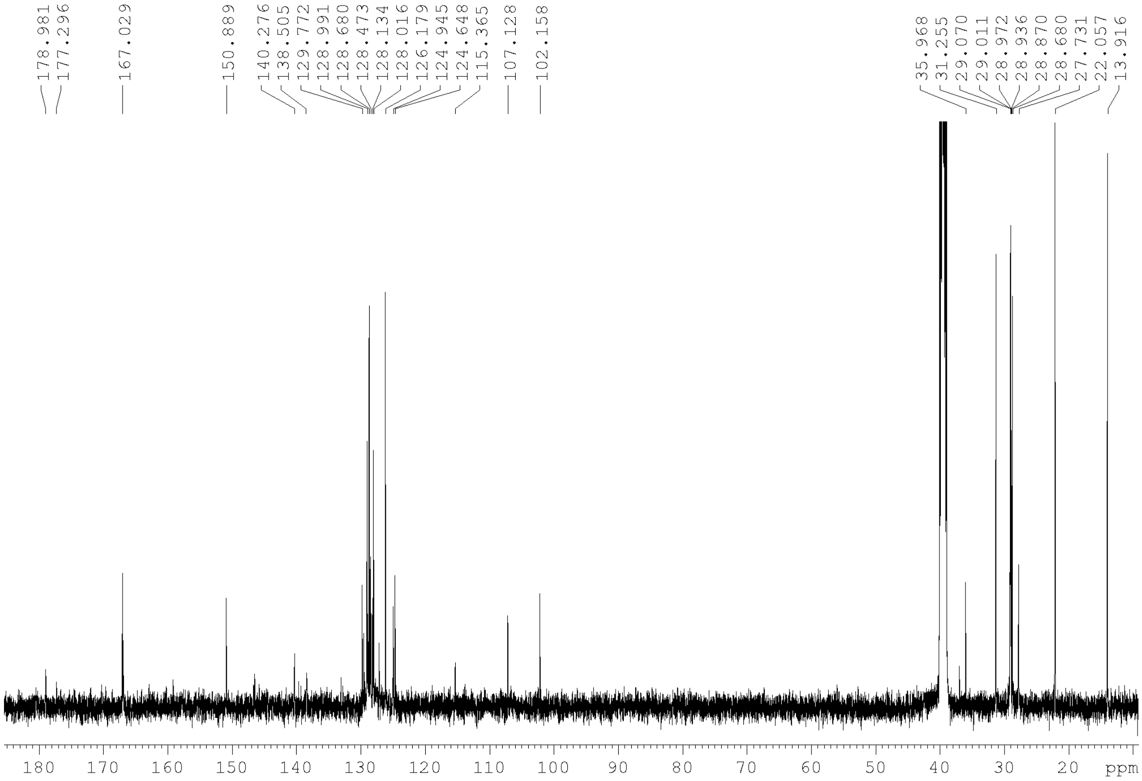

^{1}H-NMR ($CDCl_3$, 500 MHz) of compound 4j

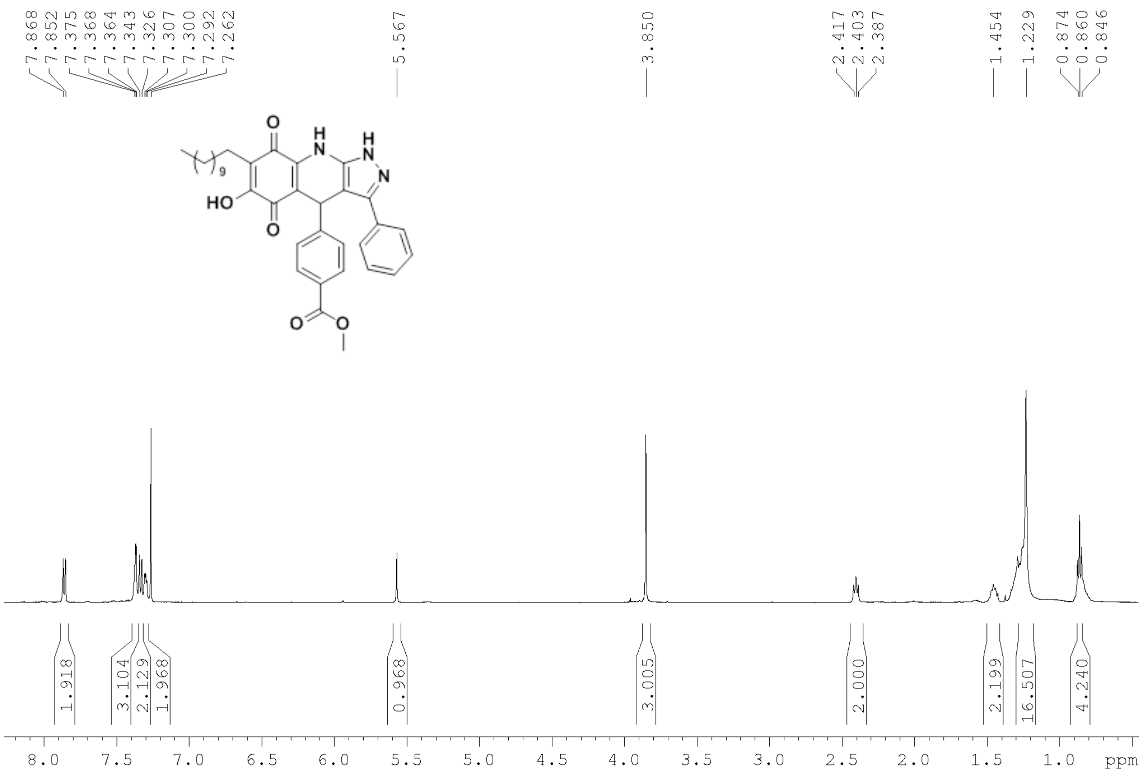

^{13}C-NMR ($CDCl_3$, 125 MHz) of compound 4j

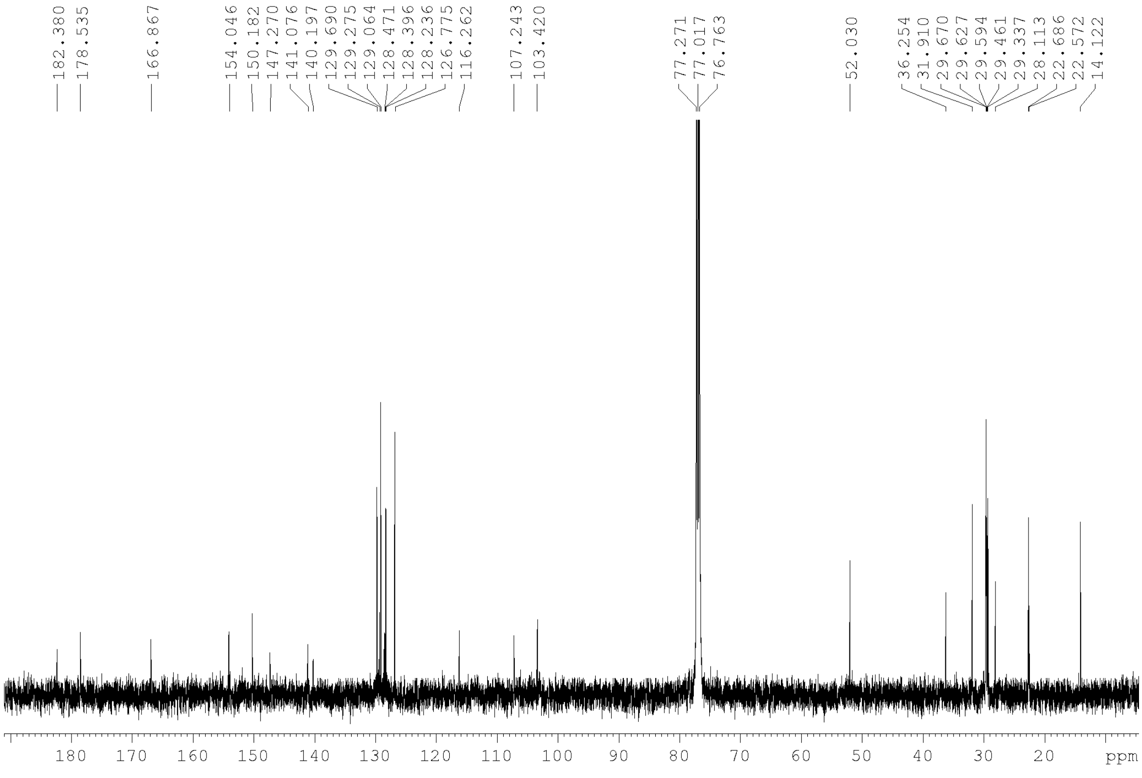

^{1}H-NMR ($CDCl_3$, 500 MHz) of compound 4k

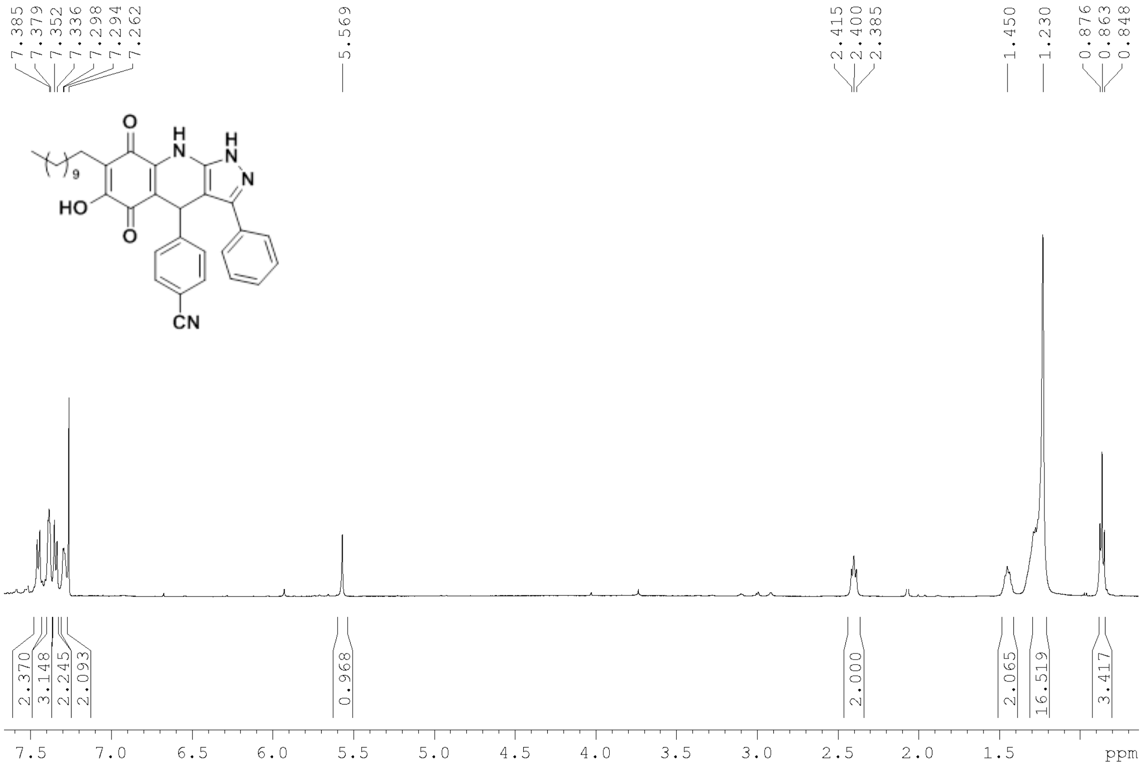

^{13}C-NMR ($CDCl_3$, 125 MHz) of compound 4k

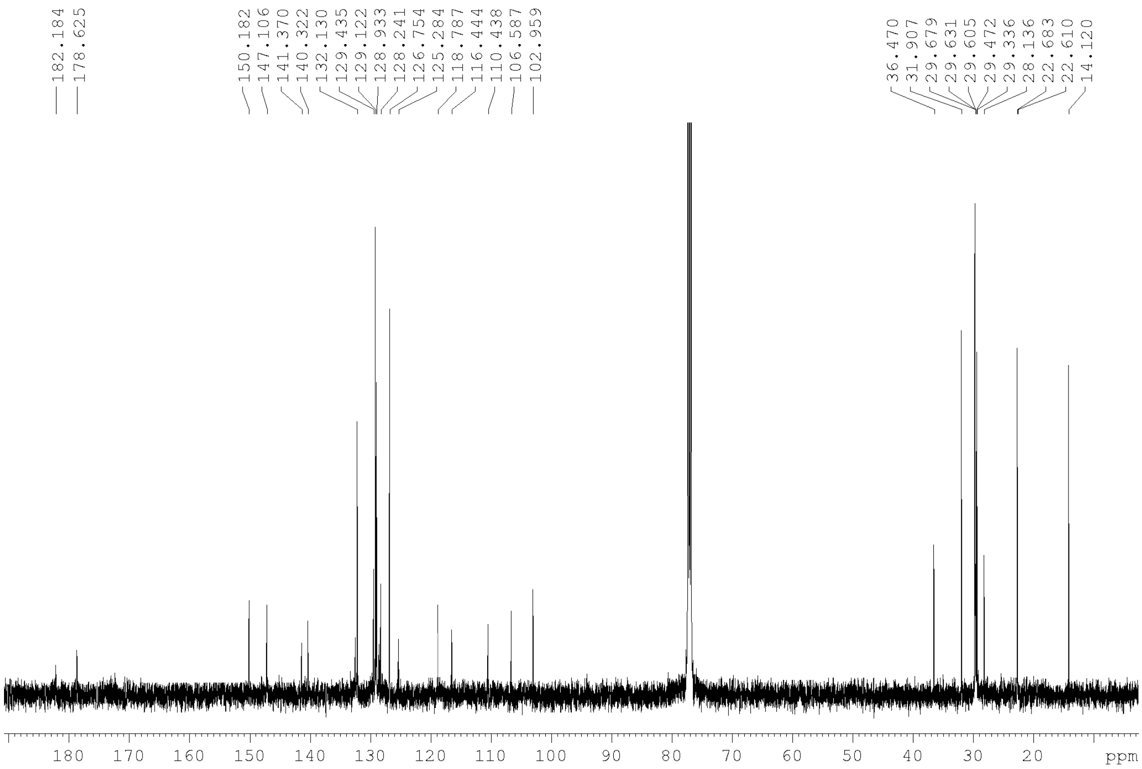

^{1}H-NMR ($CDCl_3$, 500 MHz) of compound 4l

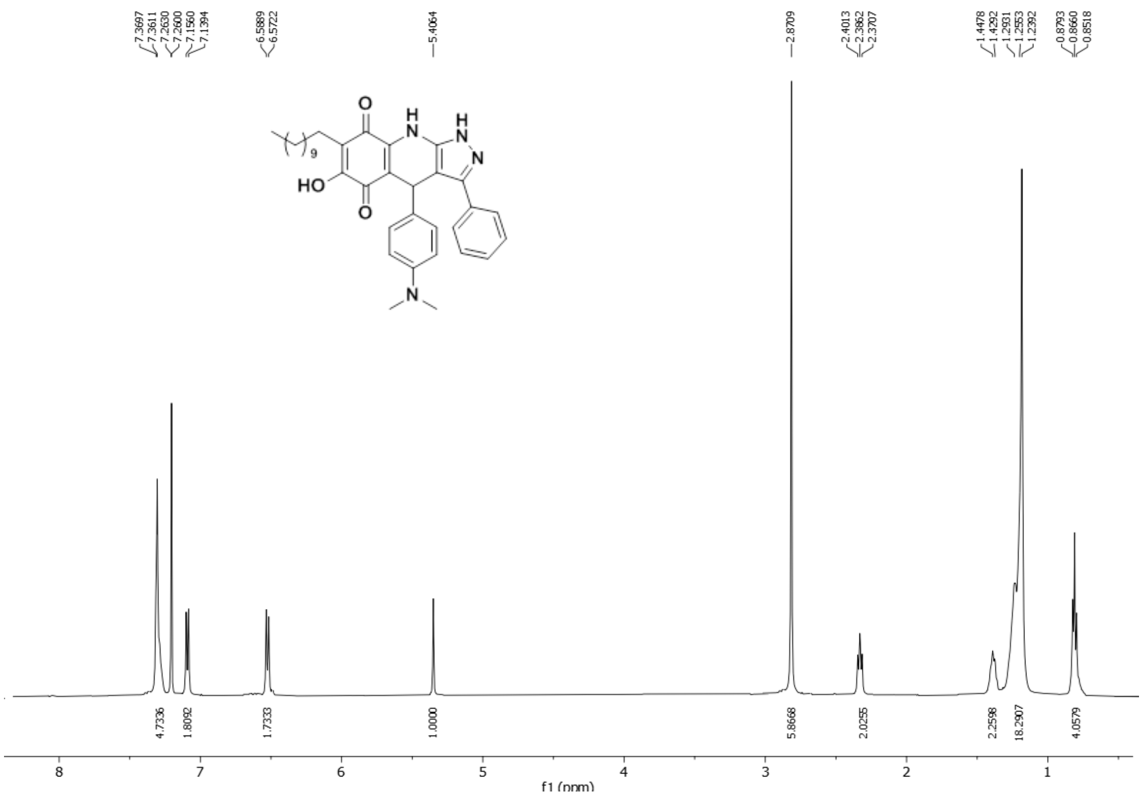

^{13}C-NMR ($CDCl_3$, 125 MHz) of compound 4l

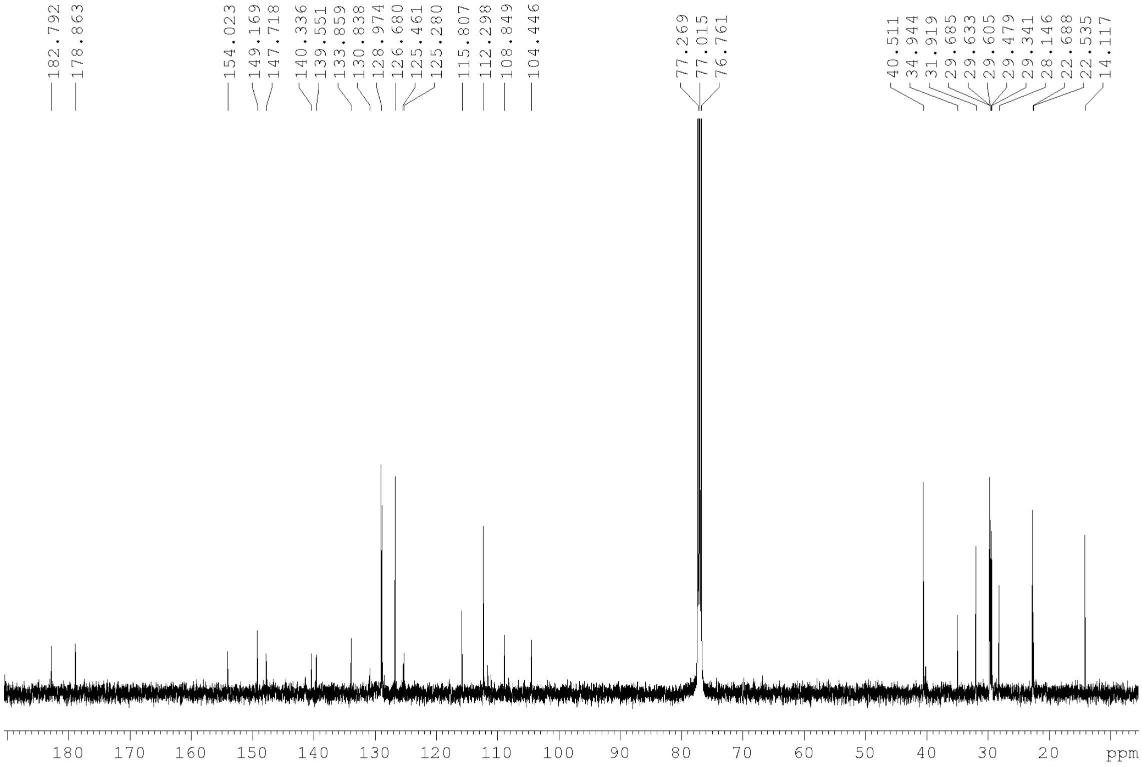

^{1}H-NMR (CDCl$_3$, 500 MHz) of compound 4m

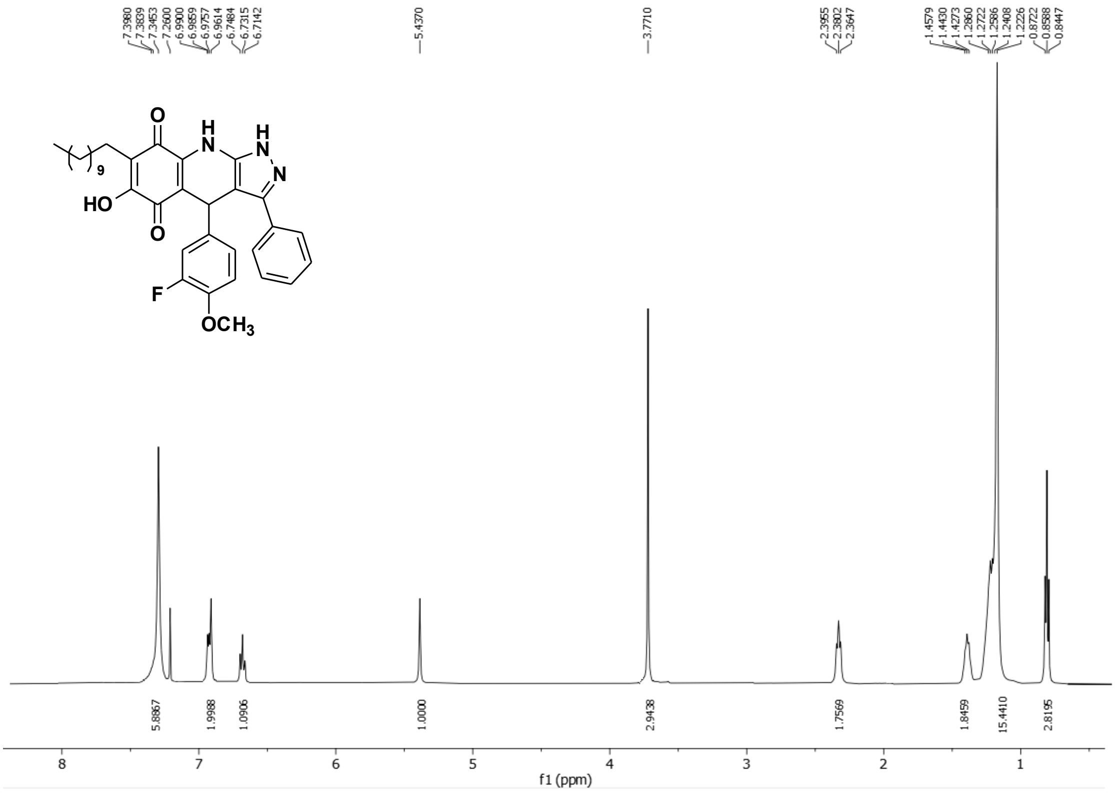

^{13}C-NMR (CDCl$_3$, 125 MHz) of compound 4m

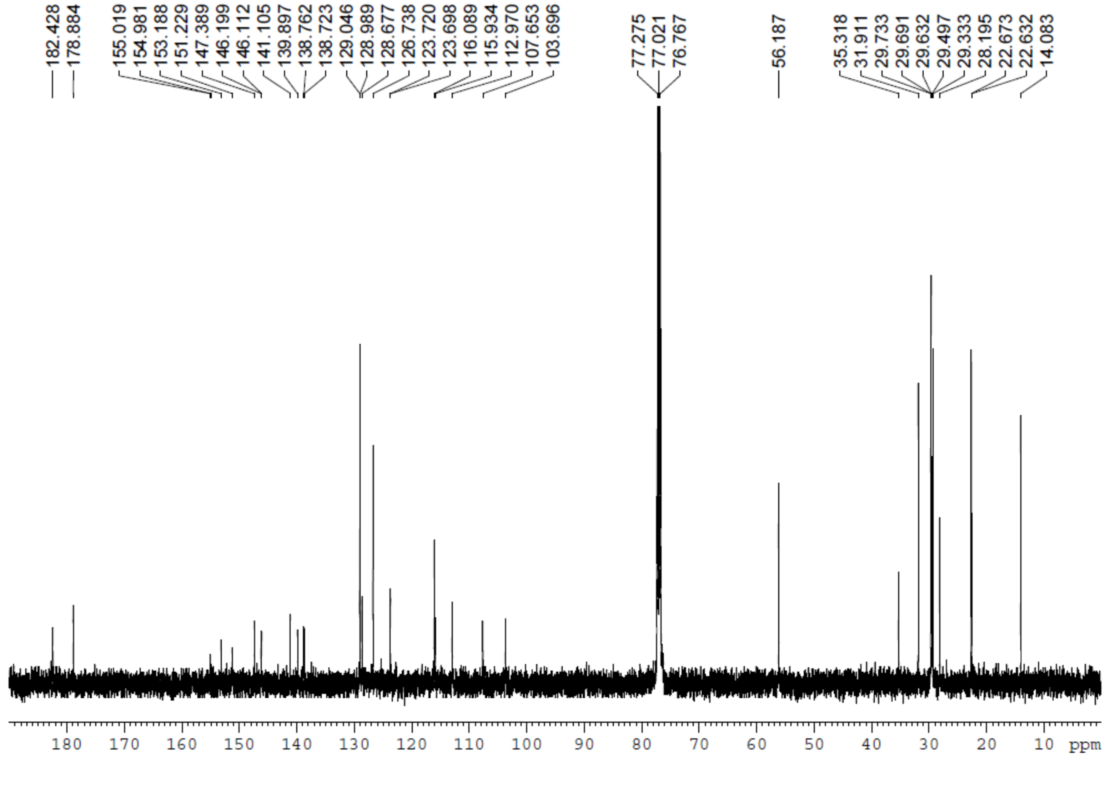

^{1}H-NMR ($CDCl_3$, 500 MHz) of compound 4n

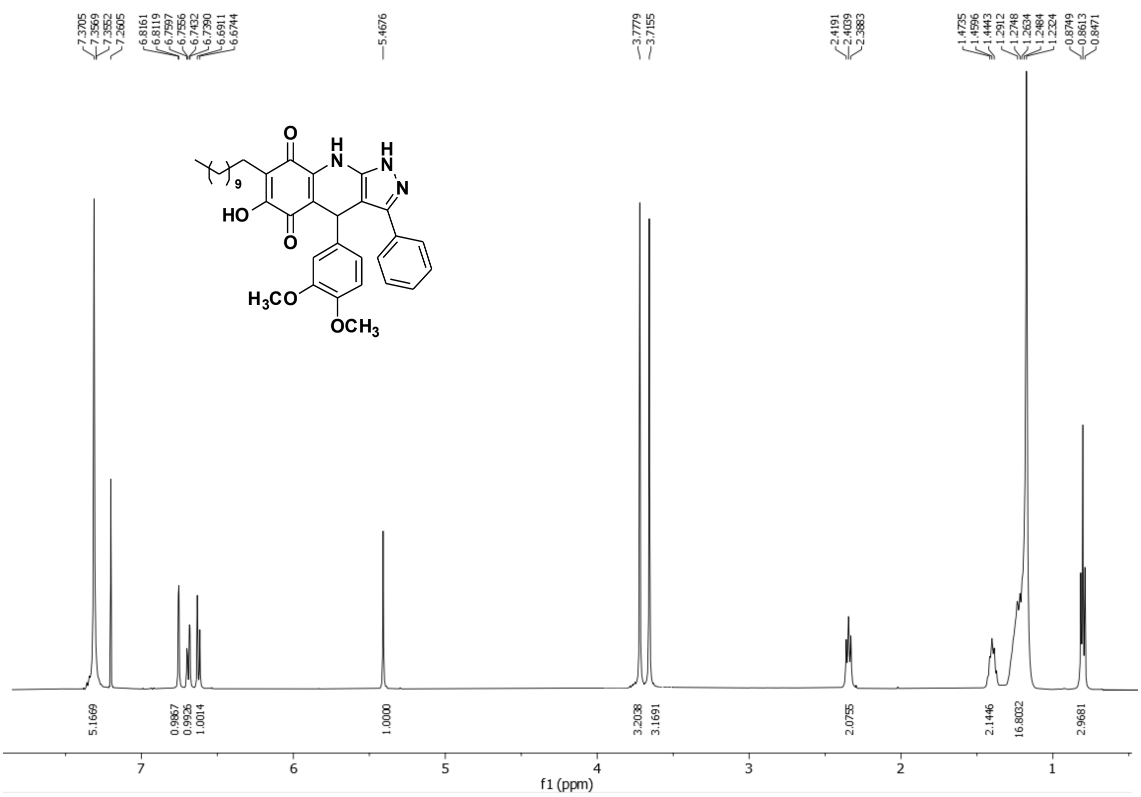

^{13}C-RMN ($CDCl_3$, 125 MHz) of compound 4n

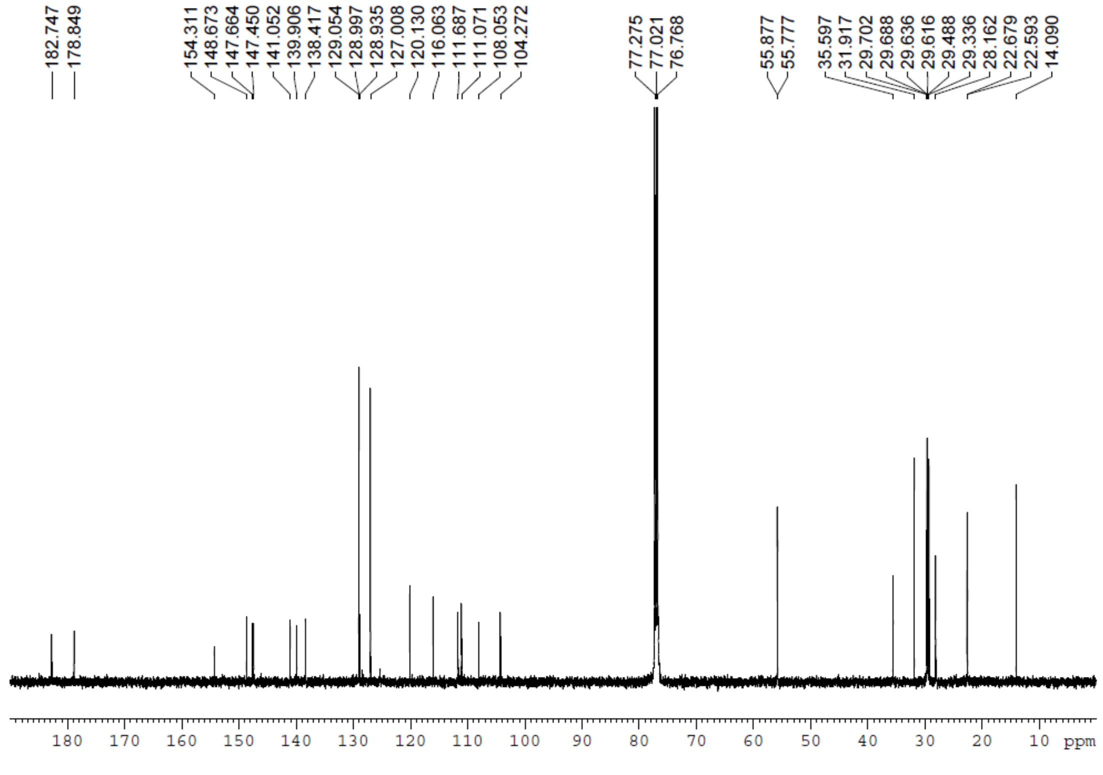

^{1}H-NMR ($CDCl_3$, 500 MHz) of compound 4o

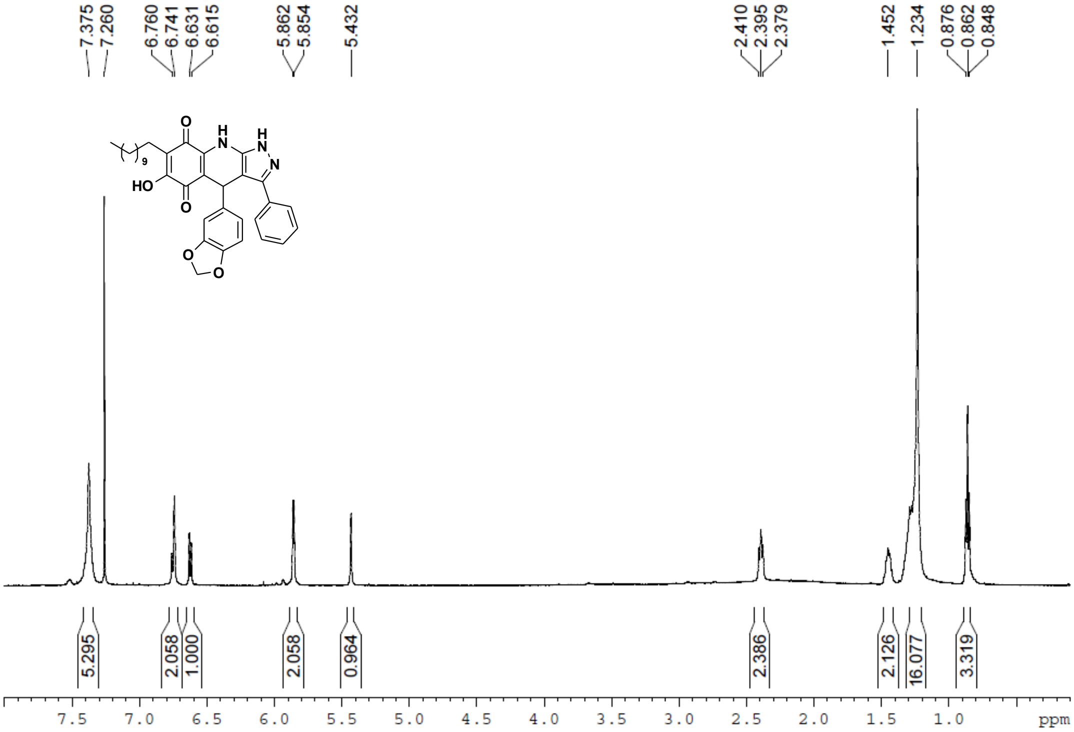

^{13}C-NMR ($CDCl_3$, 125 MHz) of compound 4o

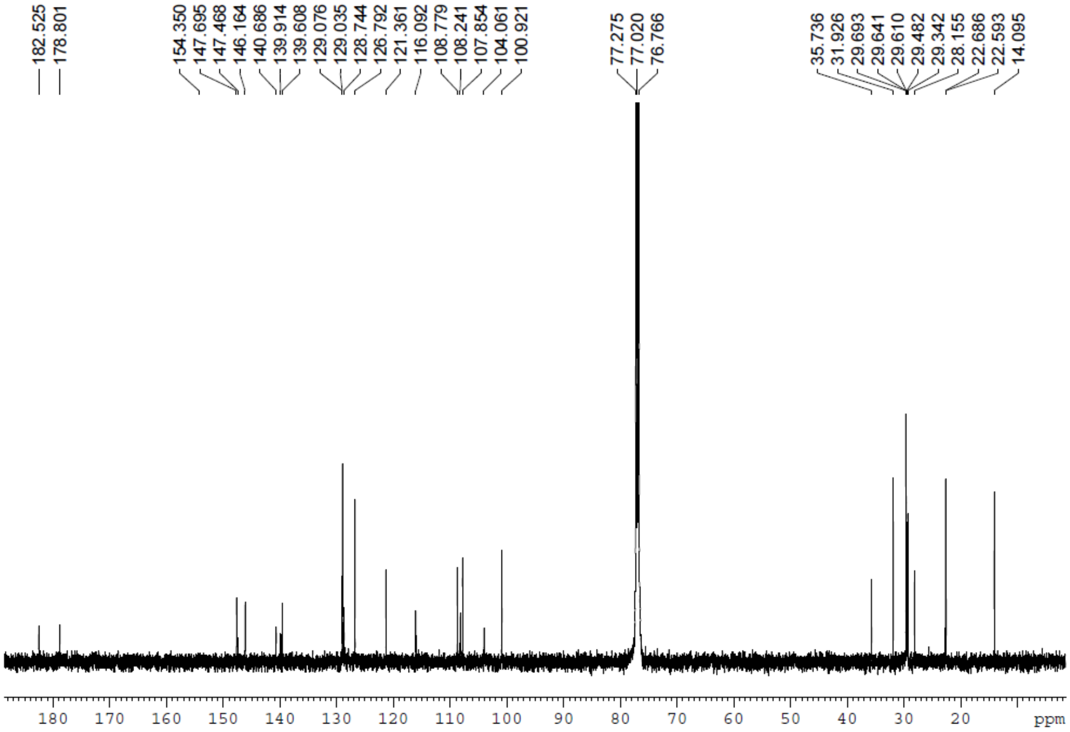

^{1}H-NMR (DMSO-d_6, 500 MHz) of compound 4p

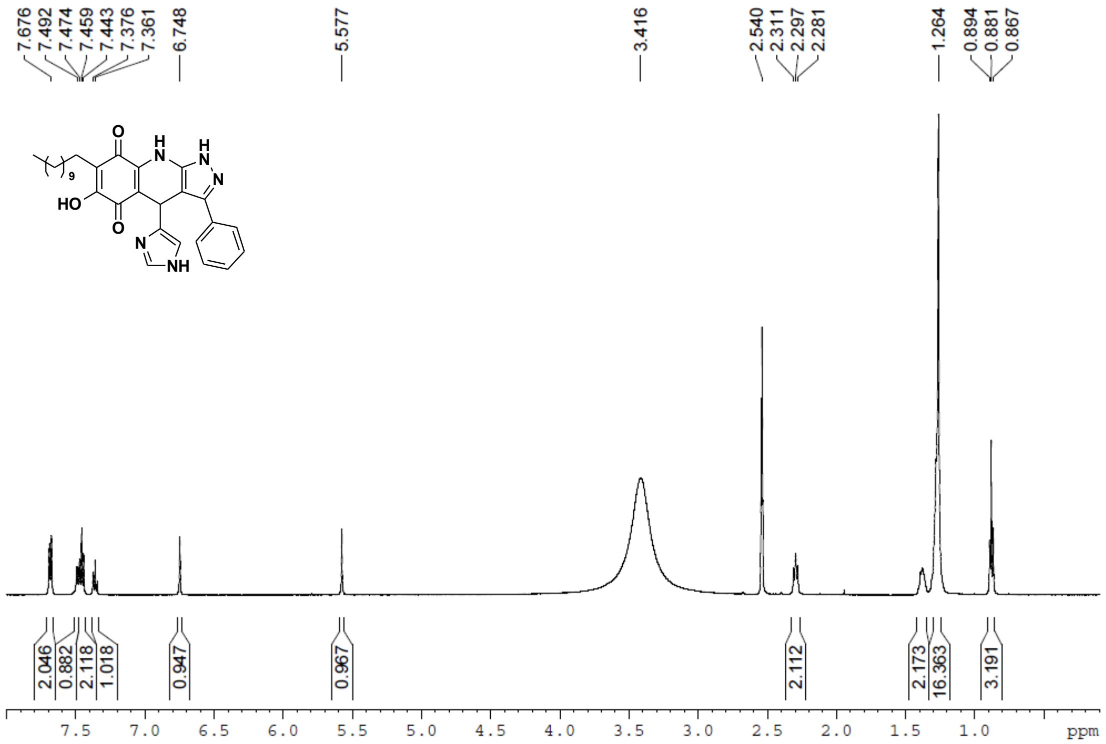

^{13}C-NMR (CDCl$_3$, 125 MHz) of compound 4p

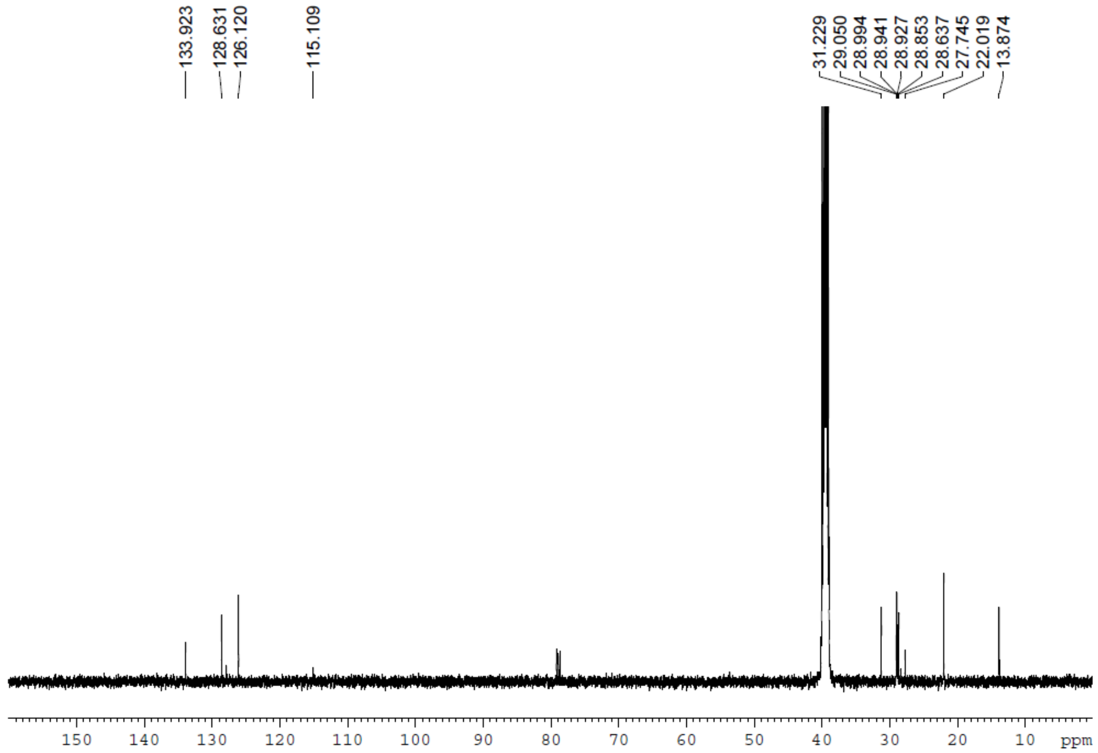

^{1}H-NMR ($CDCl_3$, 500 MHz) of compound 4r

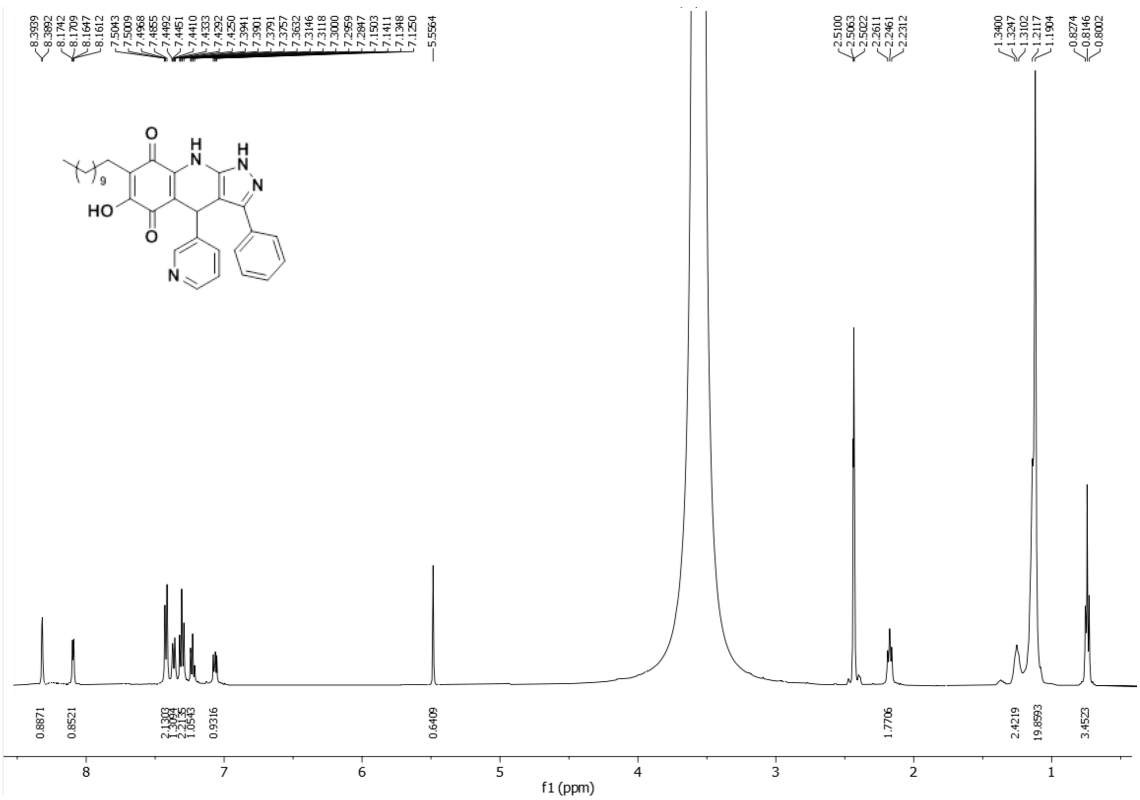

^{13}C-NMR ($CDCl_3$, 125 MHz) of compound 4r

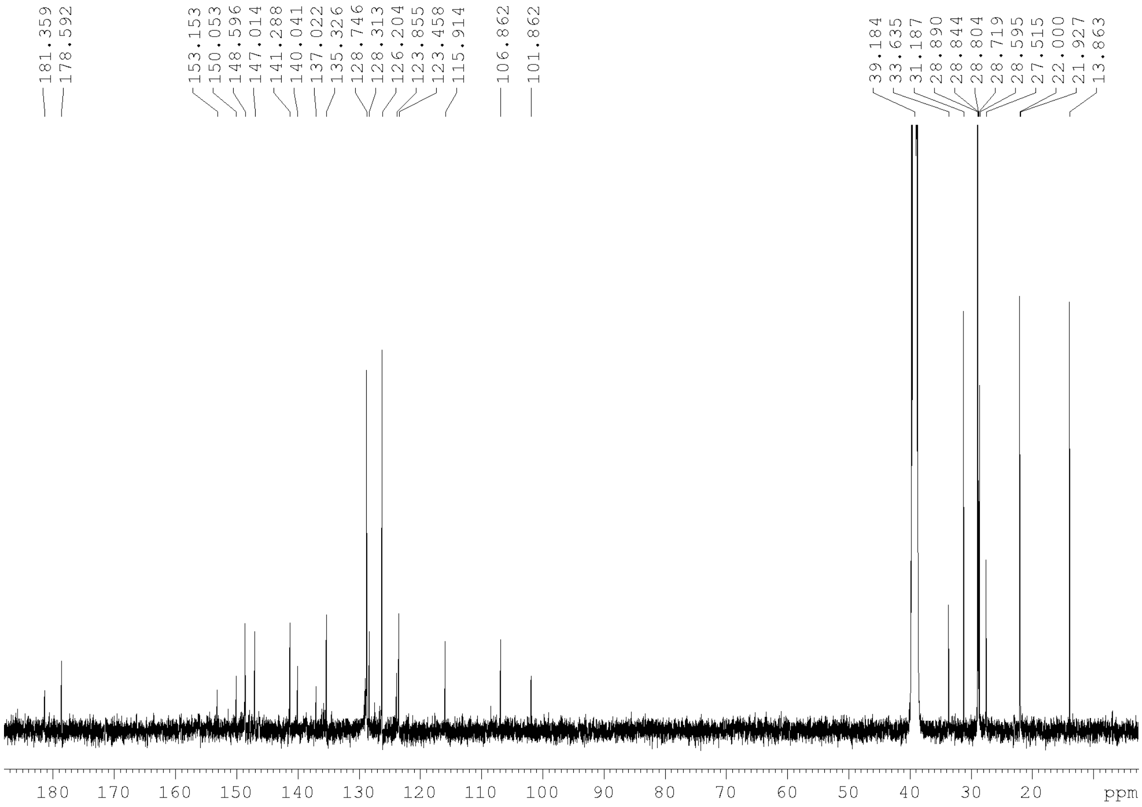

^{1}H-NMR (CDCl$_3$, 500 MHz) of compound 4s

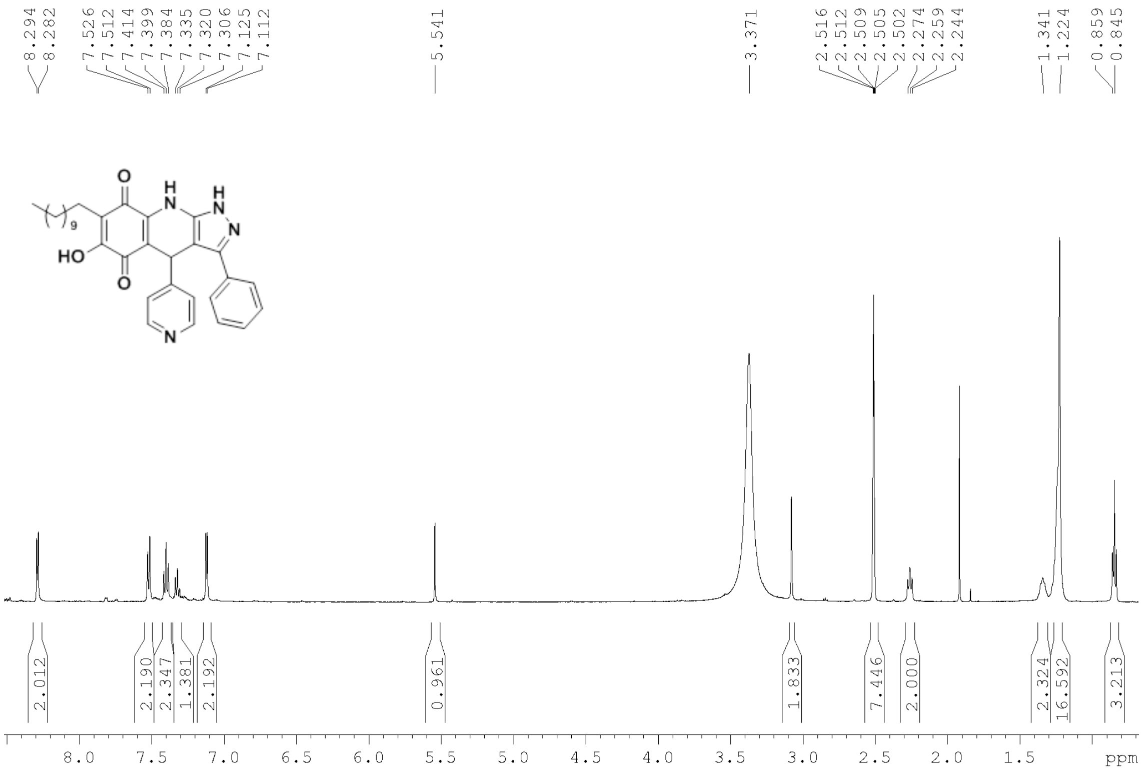

^{13}C-NMR (CDCl$_3$, 125 MHz) of compound 4s

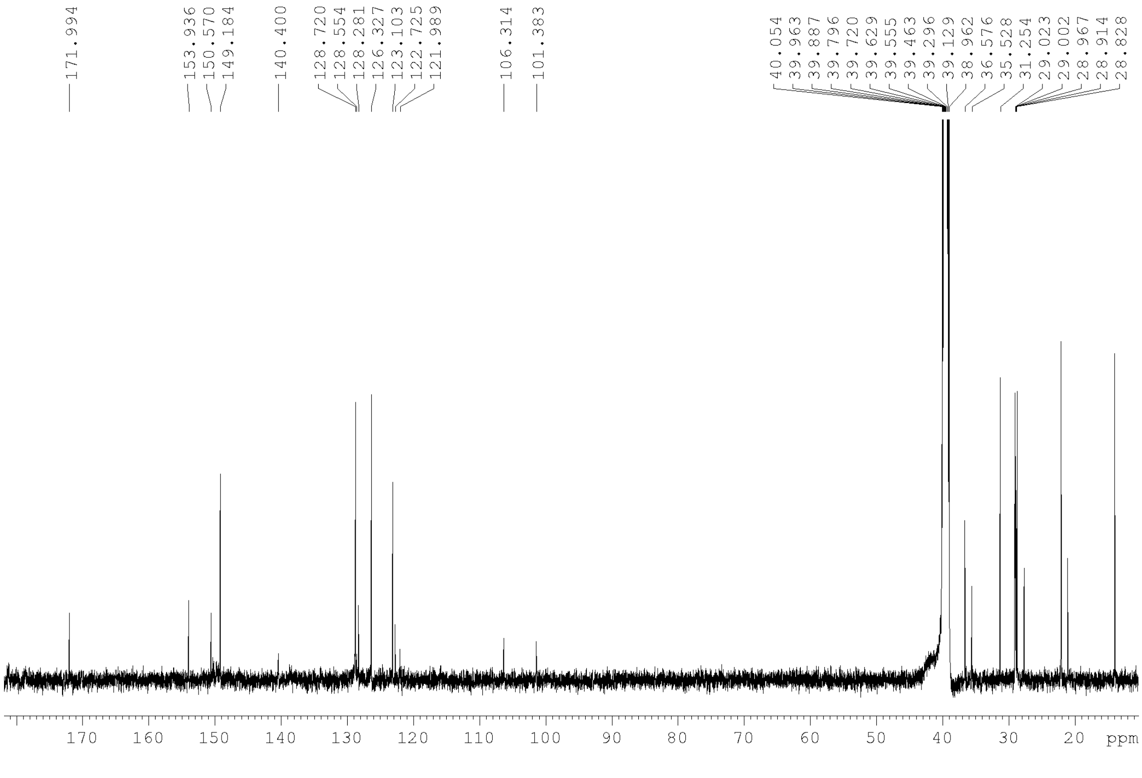

^{1}H-NMR ($CDCl_3$, 500 MHz) of compound 4t

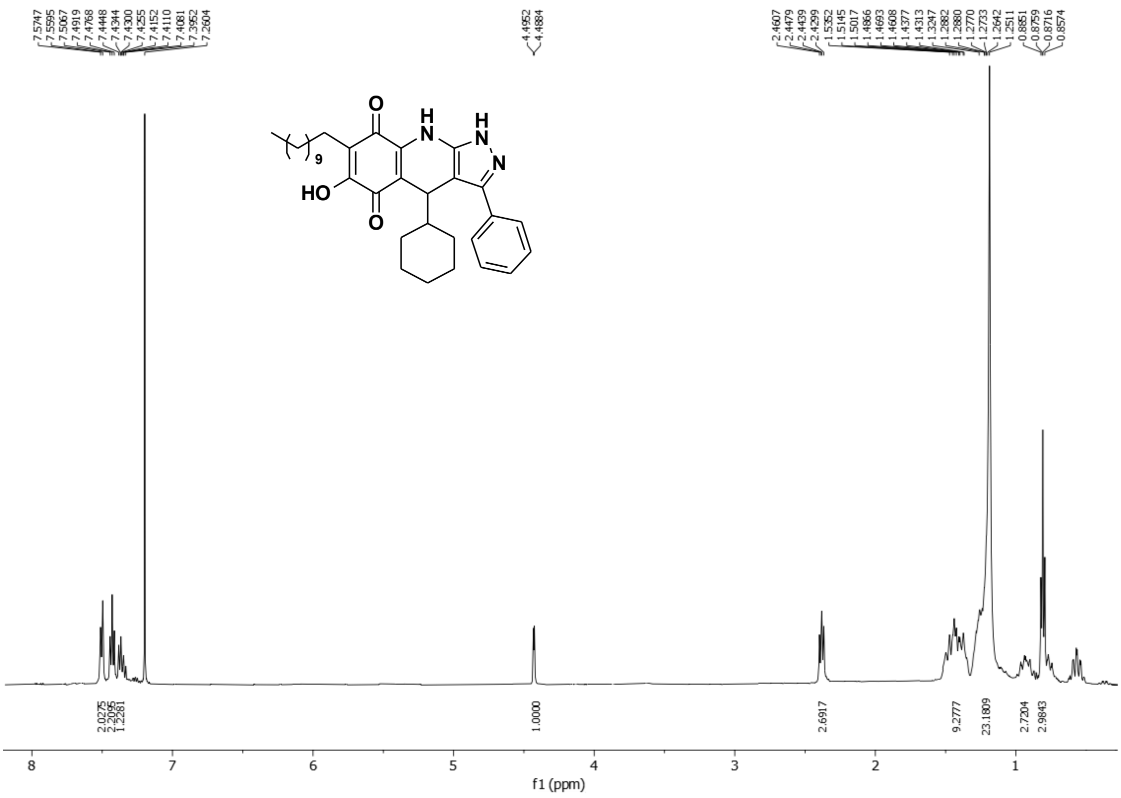

^{13}C-NMR ($CDCl_3$, 125 MHz) of compound 4t

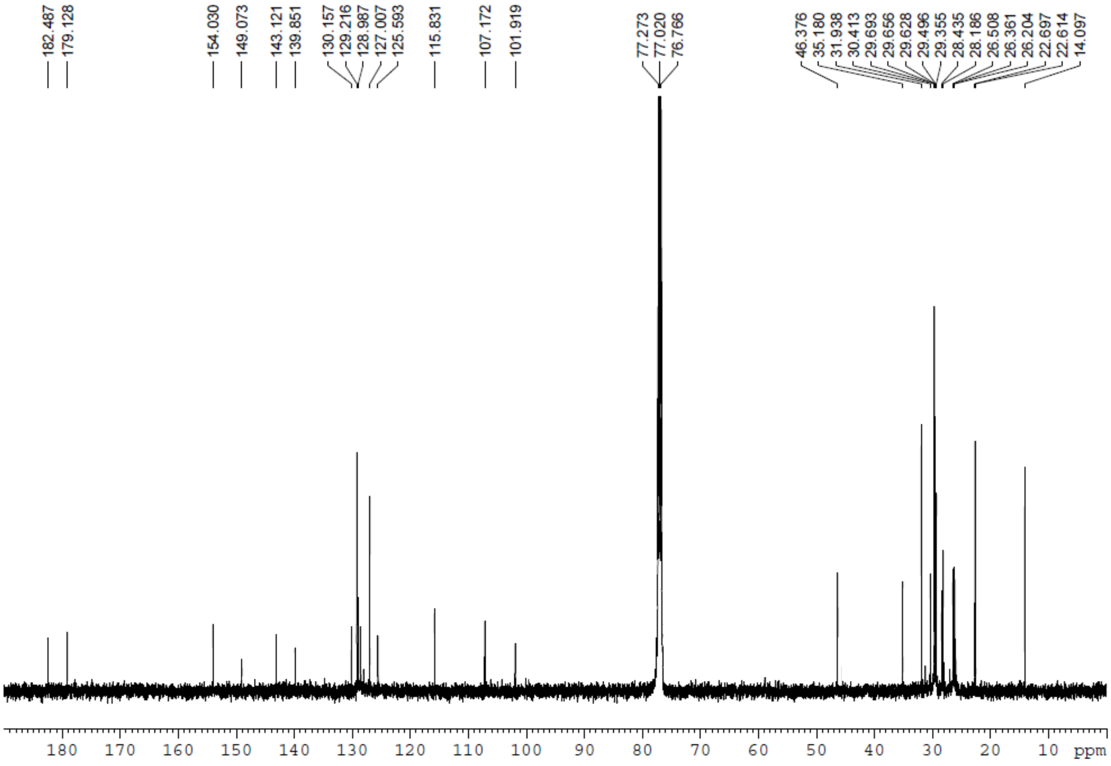

^{1}H-NMR (DMSO-d$_6$, 500 MHz) of compound 4u

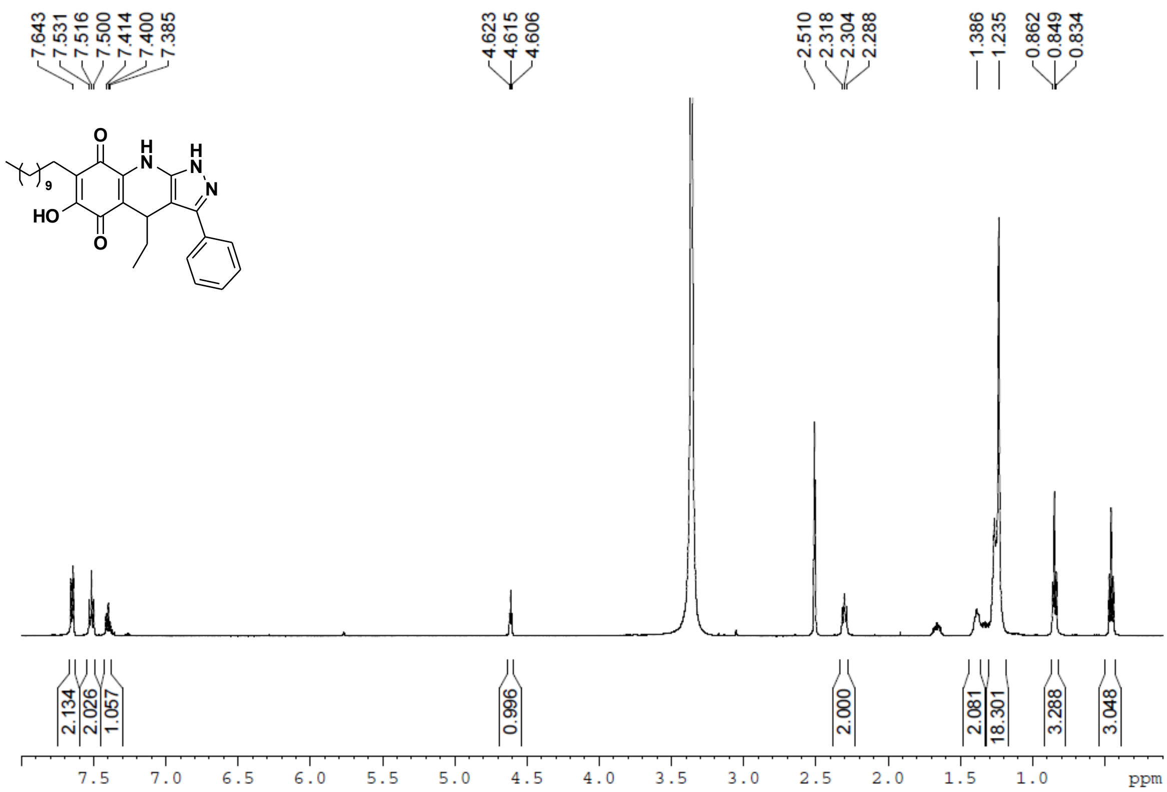

^{13}C-NMR (CDCl$_3$, 125 MHz) of compound 4u

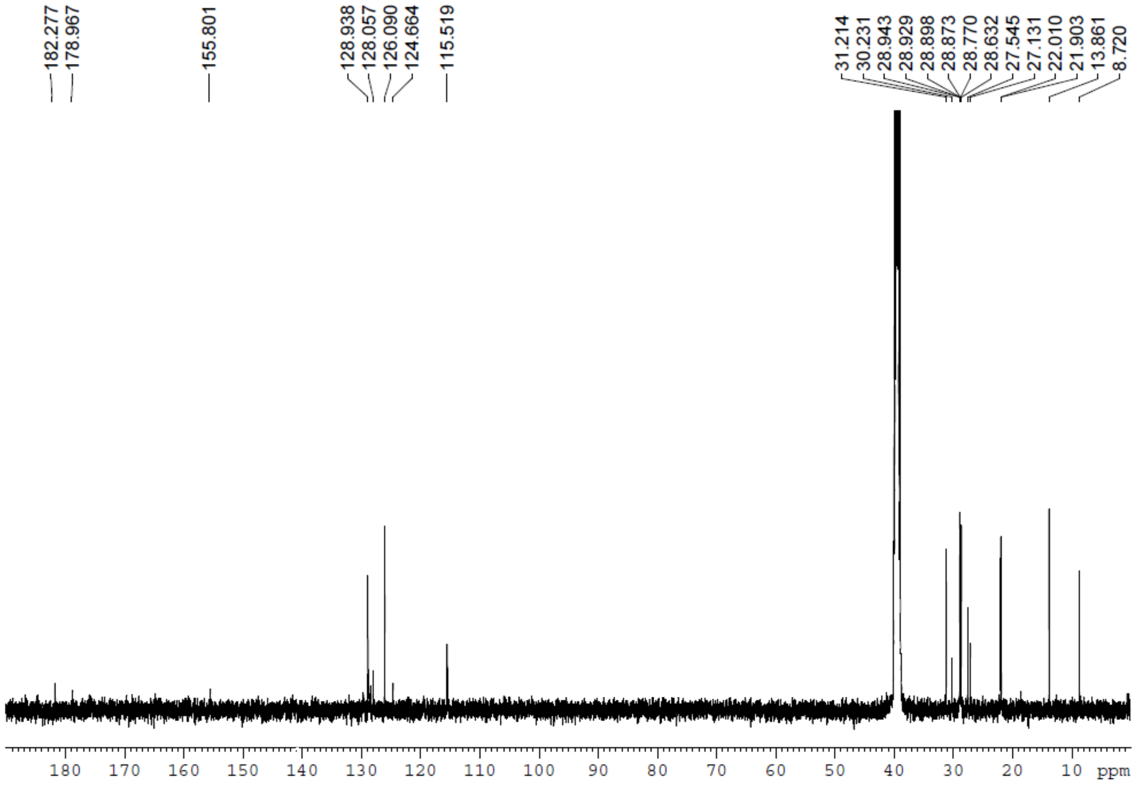

^{1}H-NMR ($CDCl_3$, 500 MHz) of compound 4v

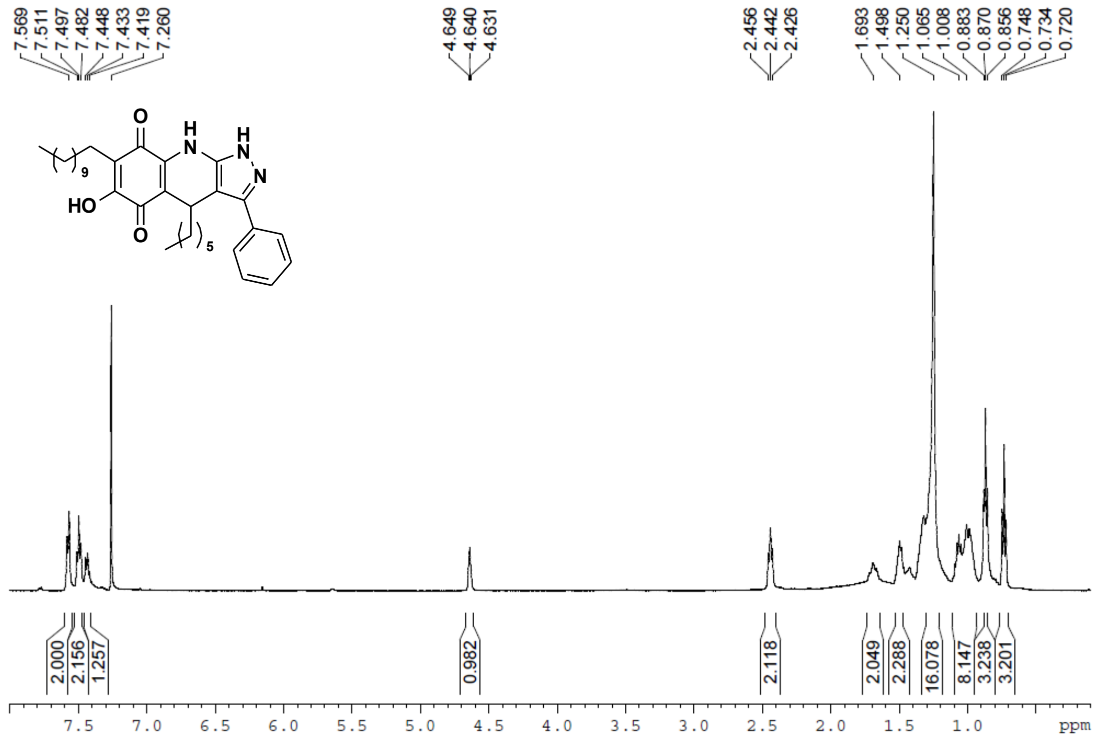

^{13}C-NMR ($CDCl_3$, 125 MHz) of compound 4v

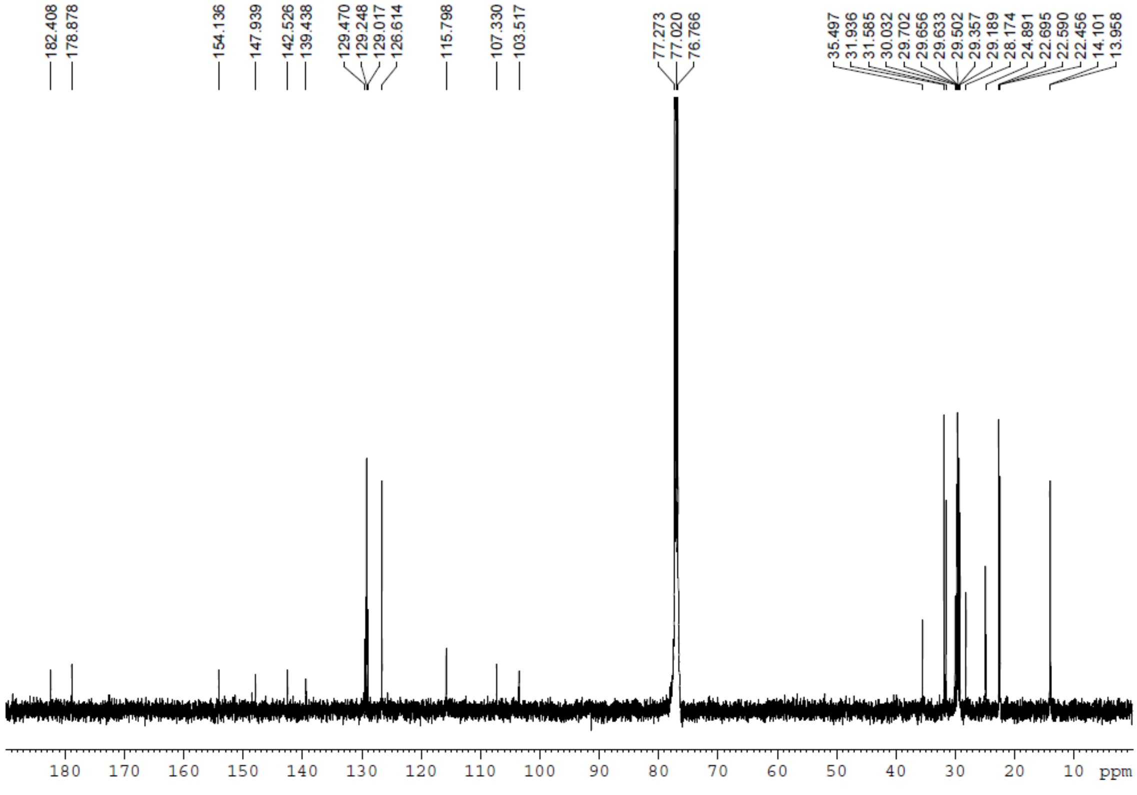

^{1}H-NMR ($CDCl_3$, 500 MHz) of compound 4w

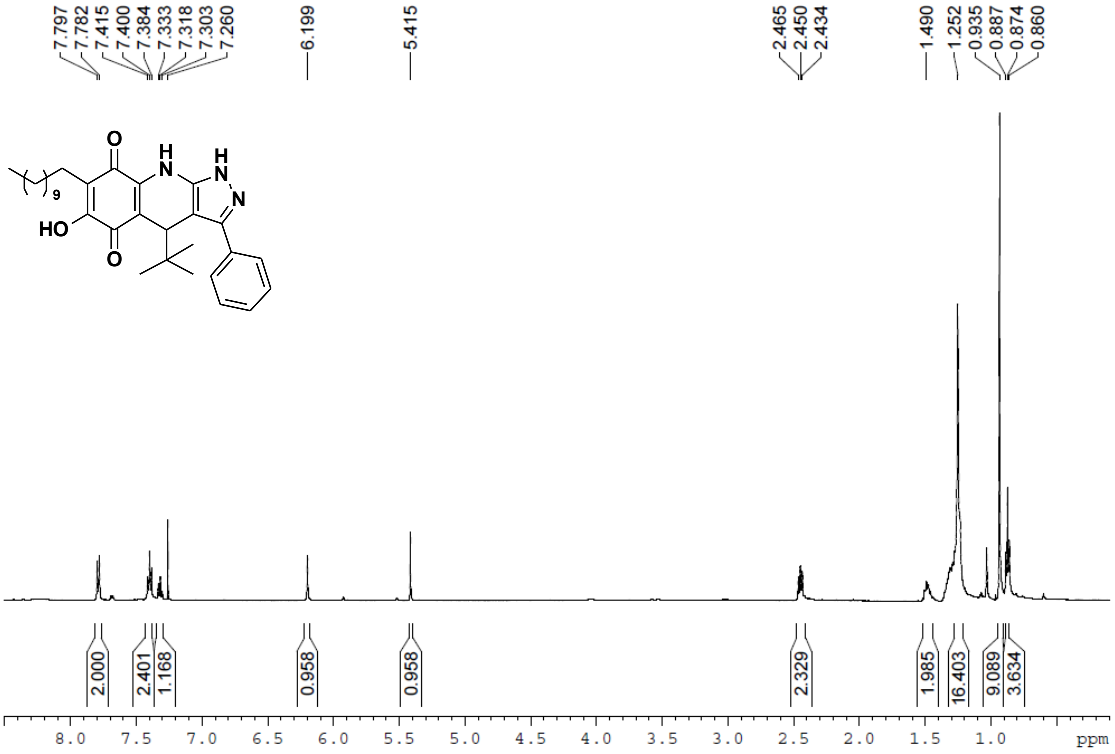

^{13}C-NMR ($CDCl_3$, 125 MHz) of compound 4w

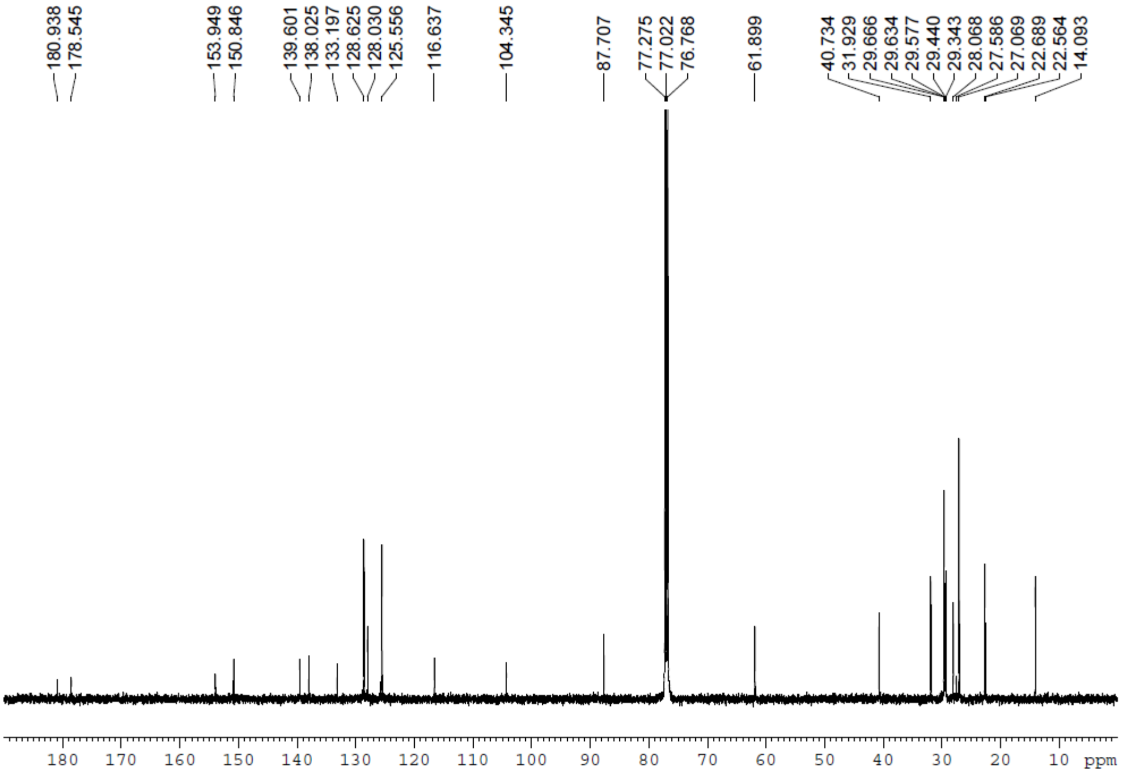

^{1}H-NMR ($CDCl_3$, 500 MHz) of compound 6

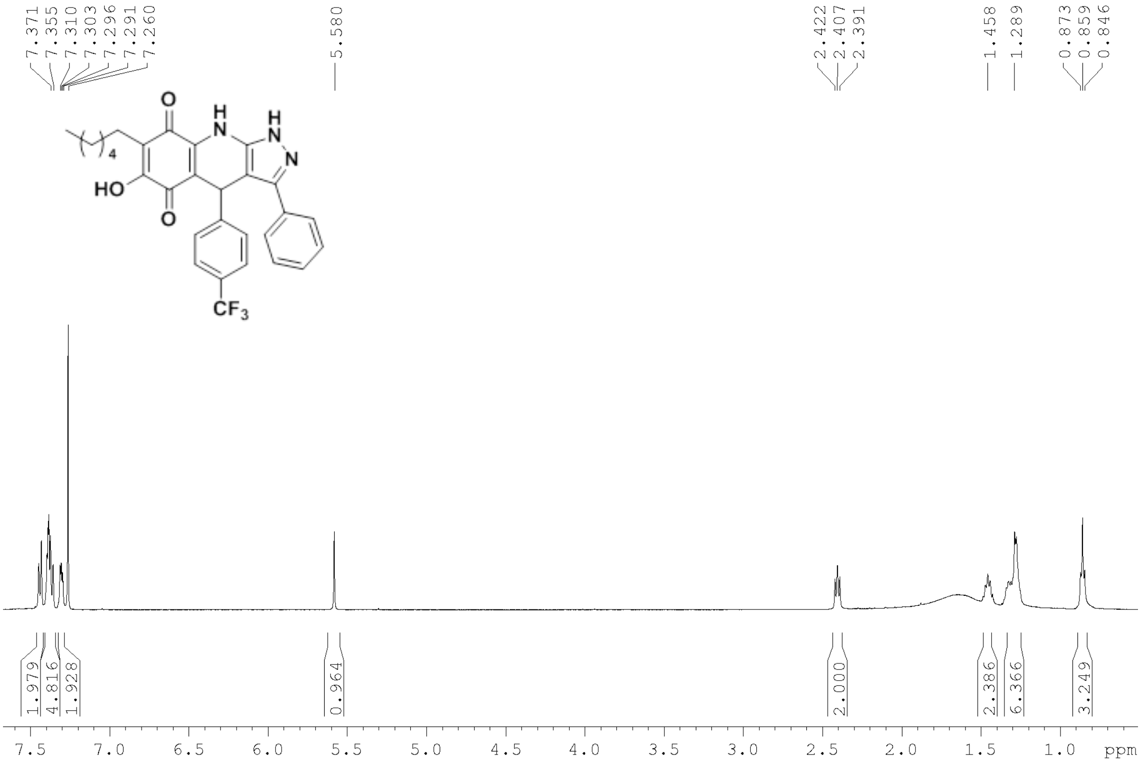

^{13}C-NMR ($CDCl_3$, 150 MHz) of compound 6

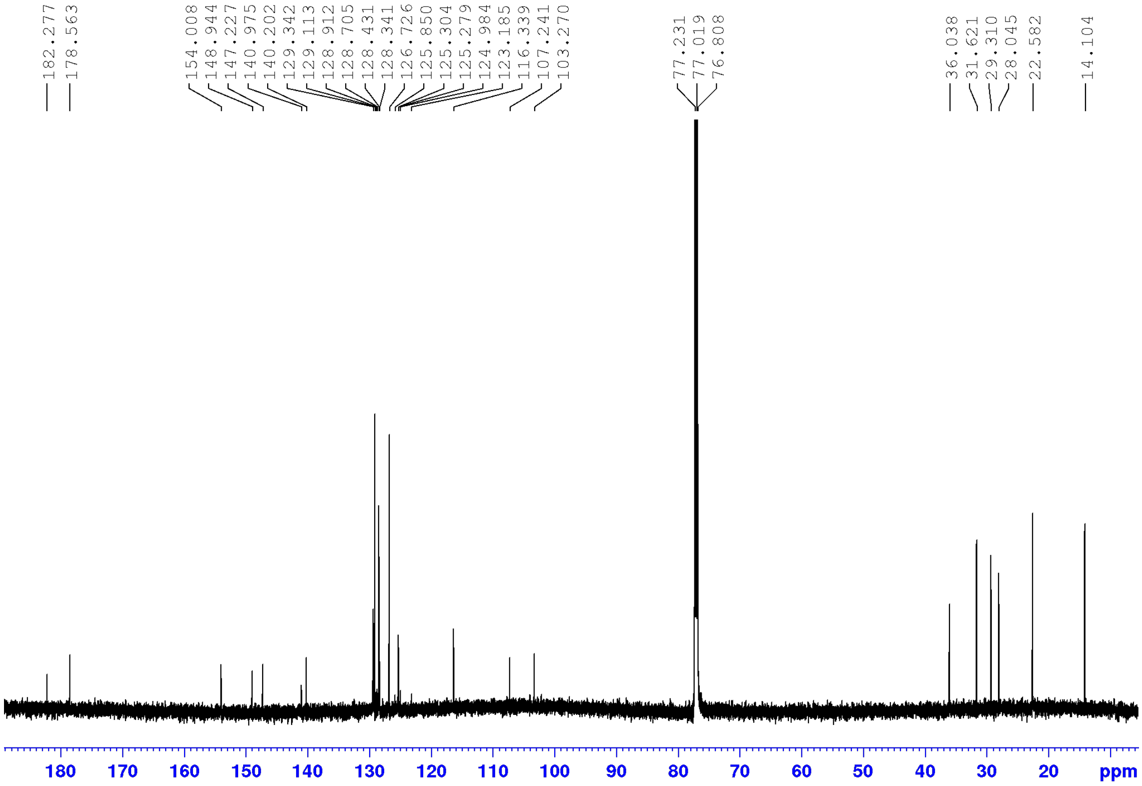

^{1}H-NMR (CDCl₃, 500 MHz) of compound 7

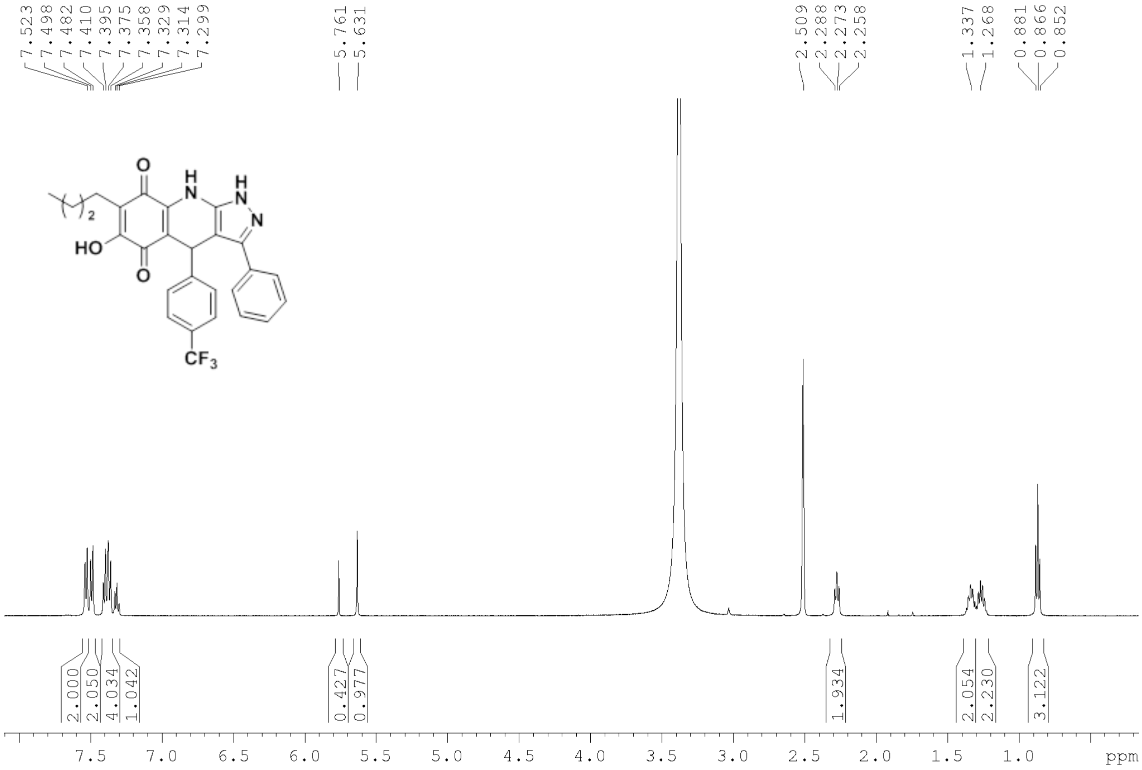

^{13}C-NMR (CDCl₃, 150 MHz) of compound 7

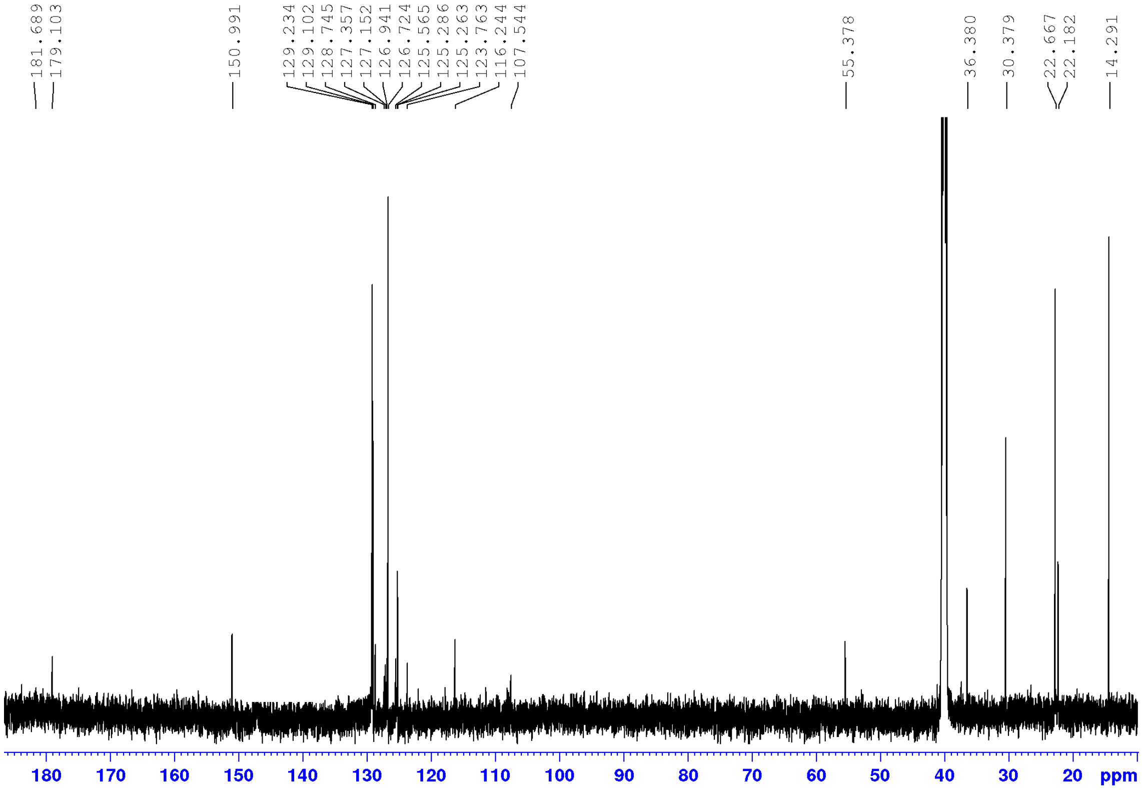

^{1}H-NMR ($CDCl_3$, 500 MHz) of compound 8

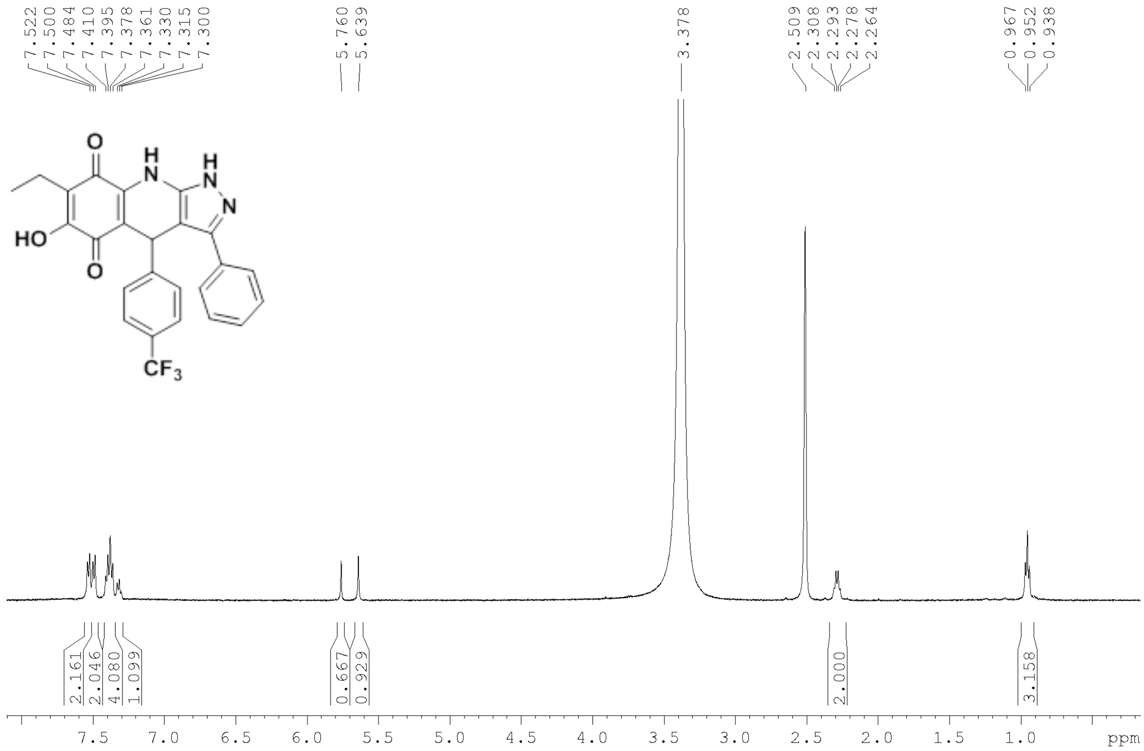

^{13}C-NMR ($CDCl_3$, 125 MHz) of compound 8

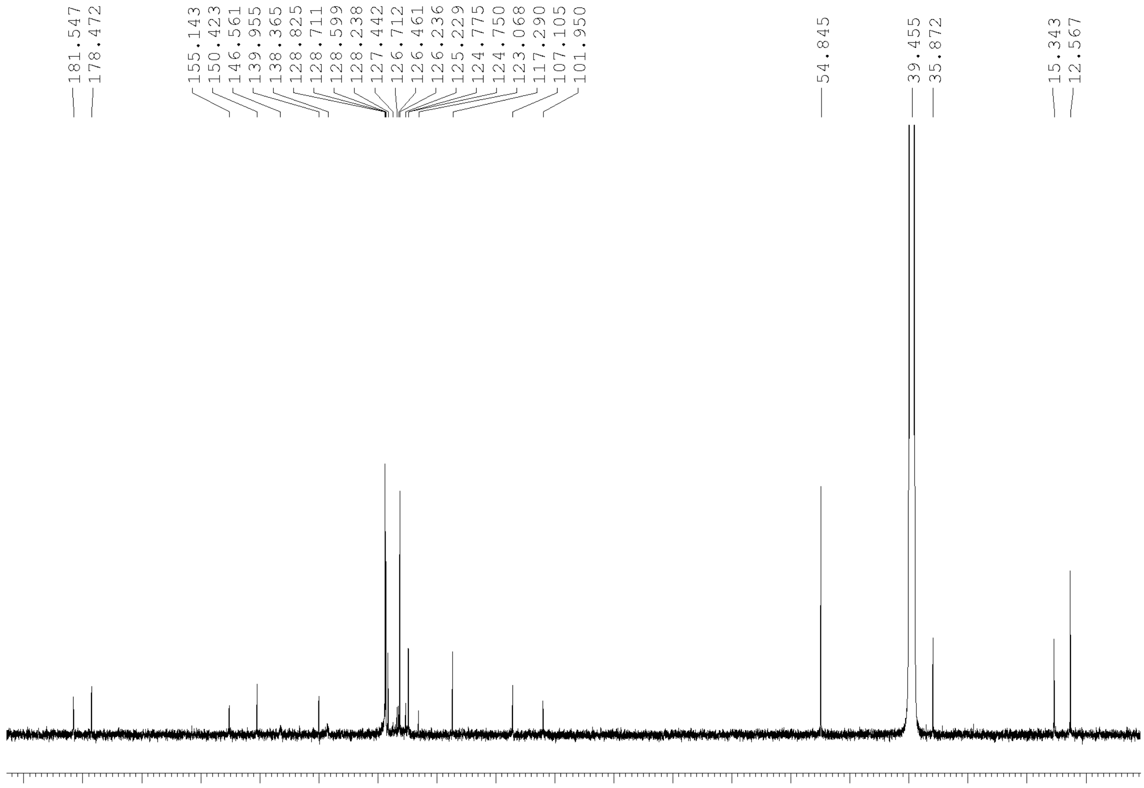

^{1}H-NMR ($CDCl_3$, 500 MHz) of compound 9

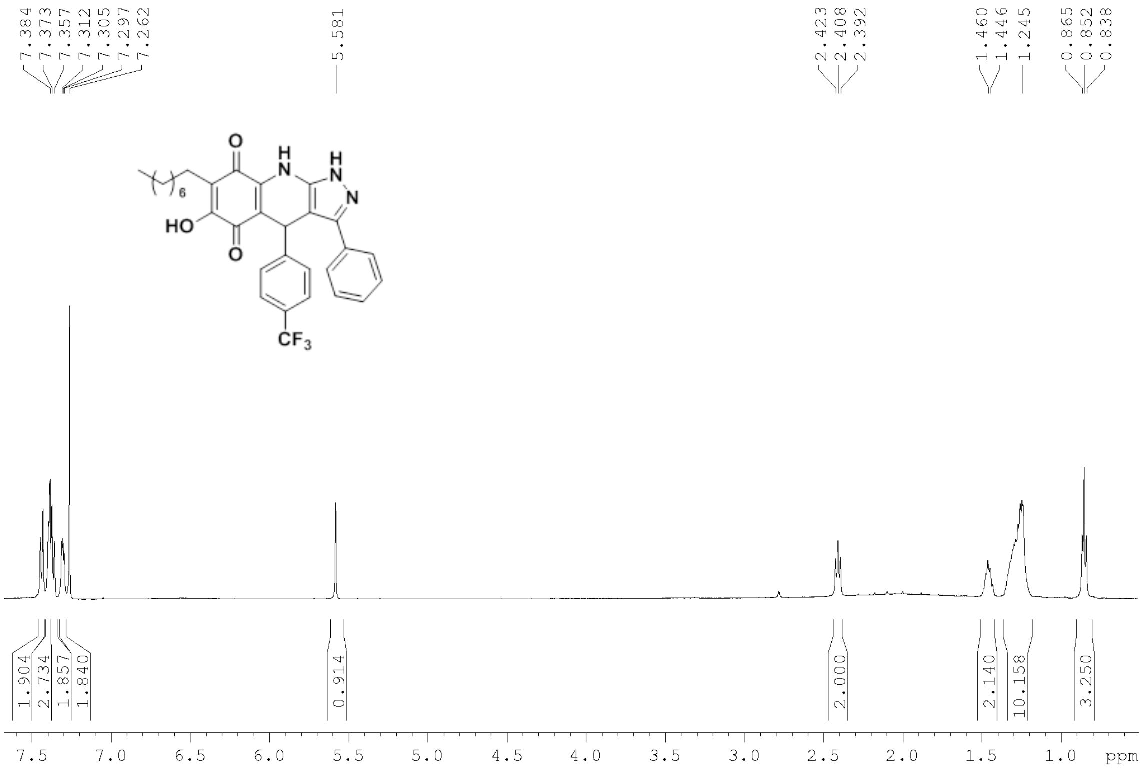

^{13}C-NMR ($CDCl_3$, 150 MHz) of compound 9

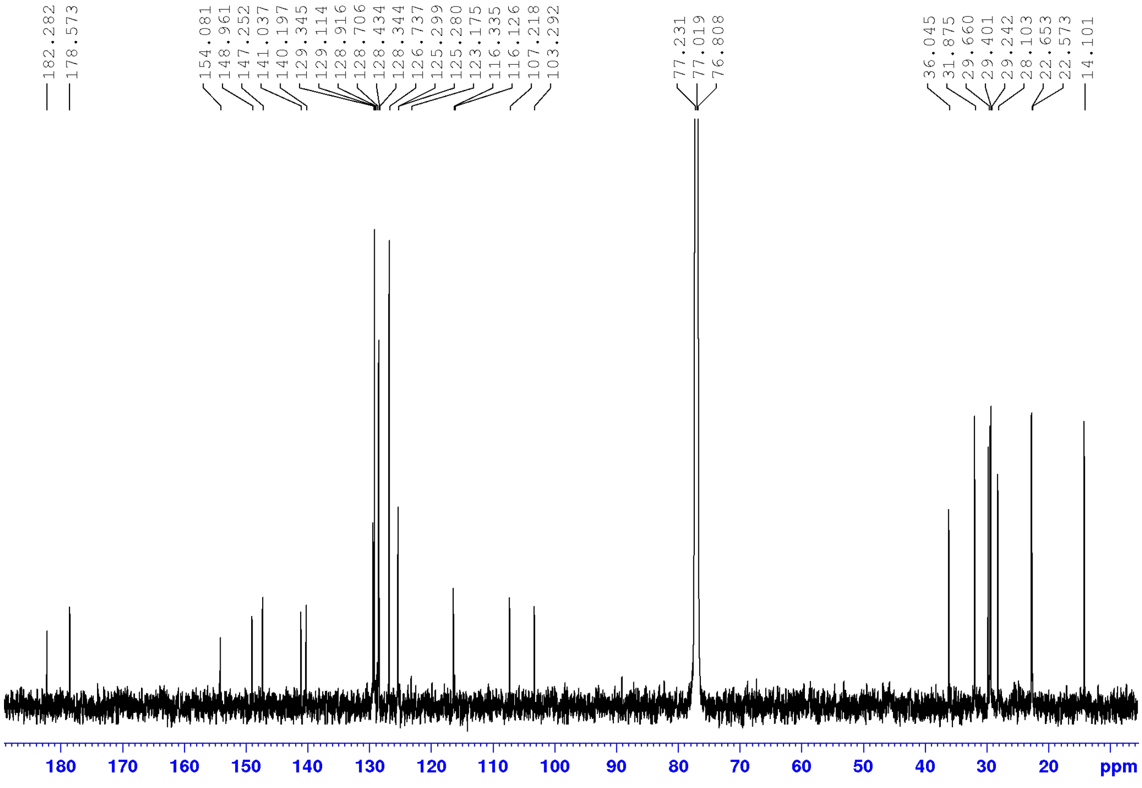

^{1}H-NMR (DMSO-d$_6$, 500 MHz) of compound 14a

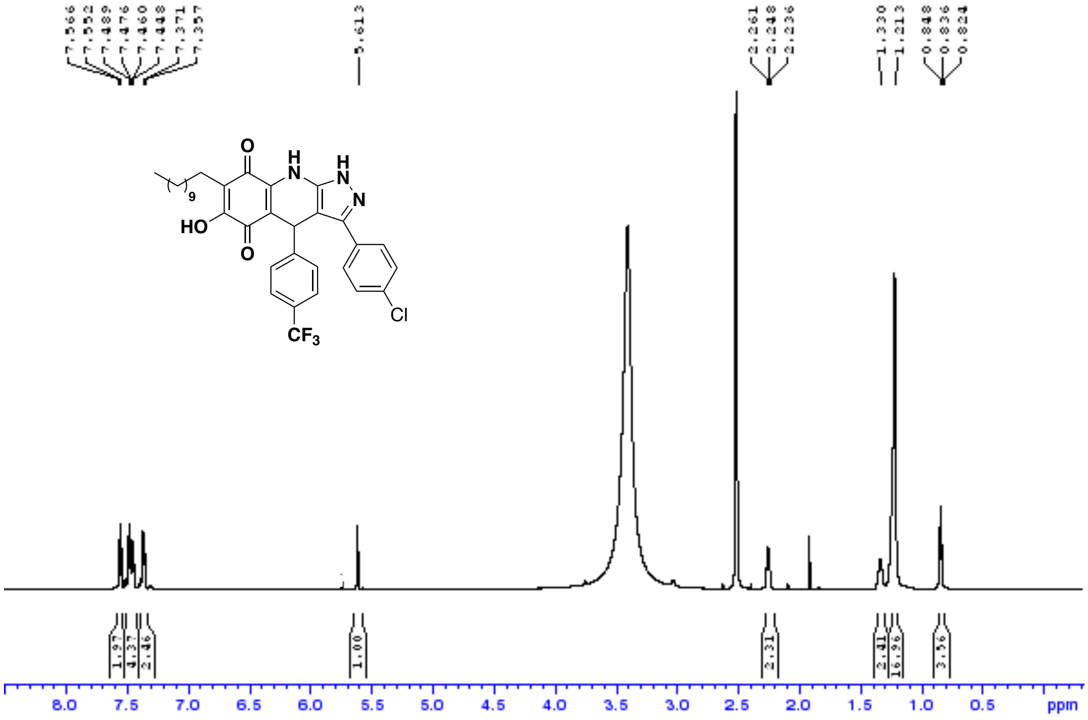

^{13}C-NMR (DMSO-d$_6$, 125 MHz) of compound 14a

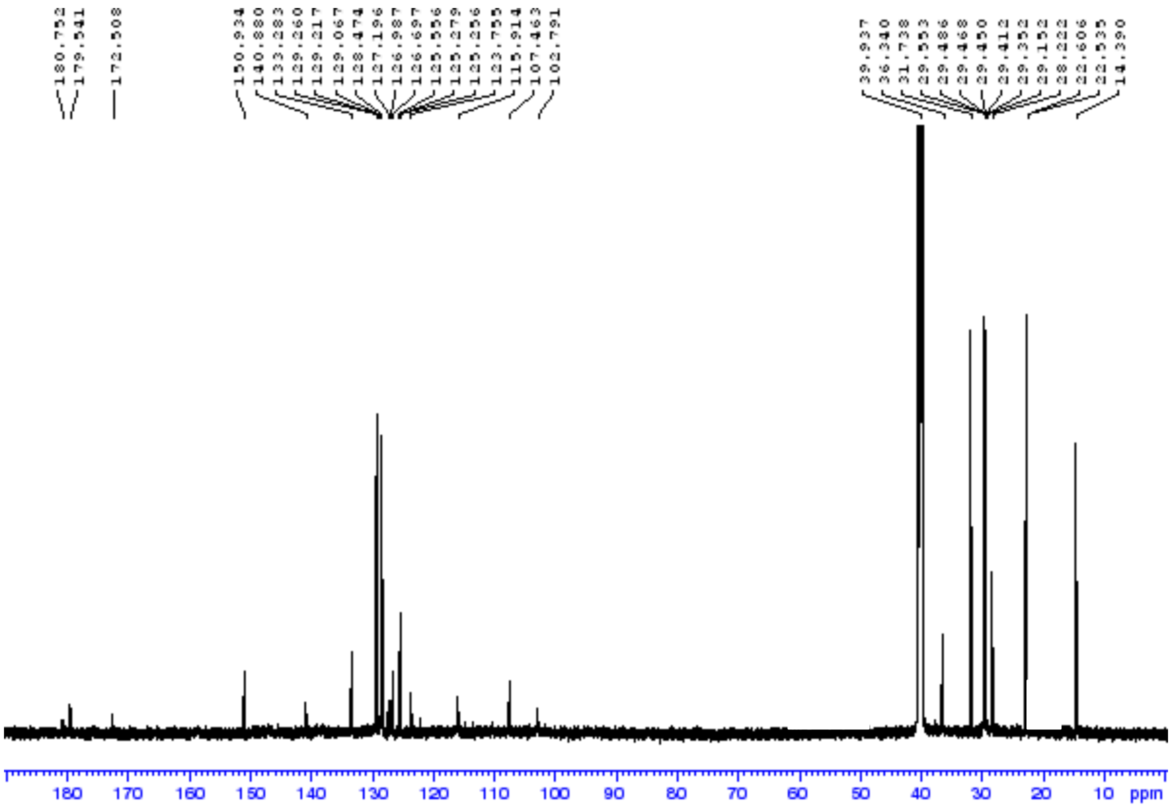

^{1}H-NMR (DMSO-d$_6$, 500 MHz) of compound 14b

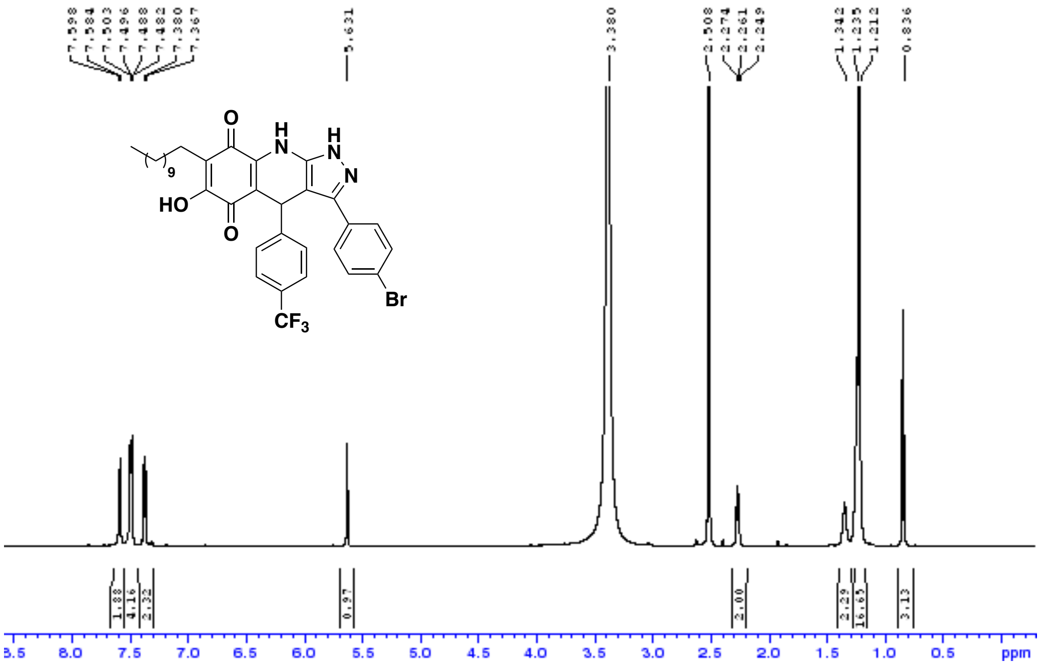

^{13}C-NMR (DMSO-d$_6$, 125 MHz) of compound 14b

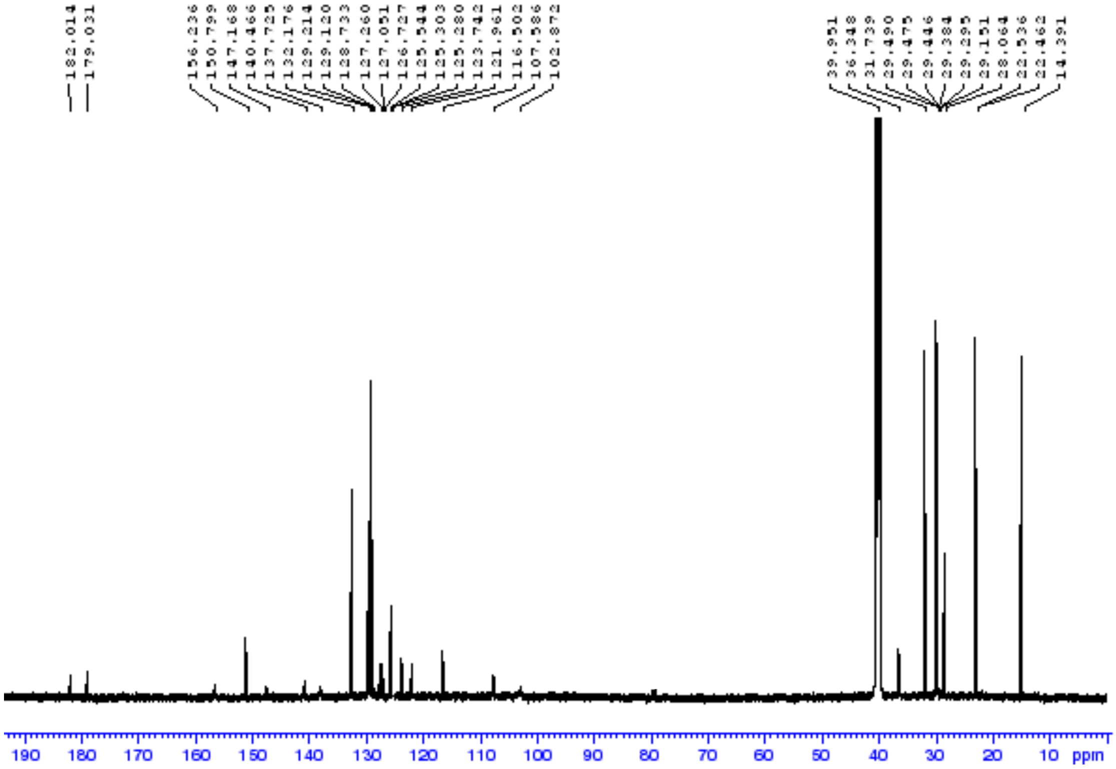

^{1}H-NMR (DMSO-d$_6$, 500 MHz) of compound 14c

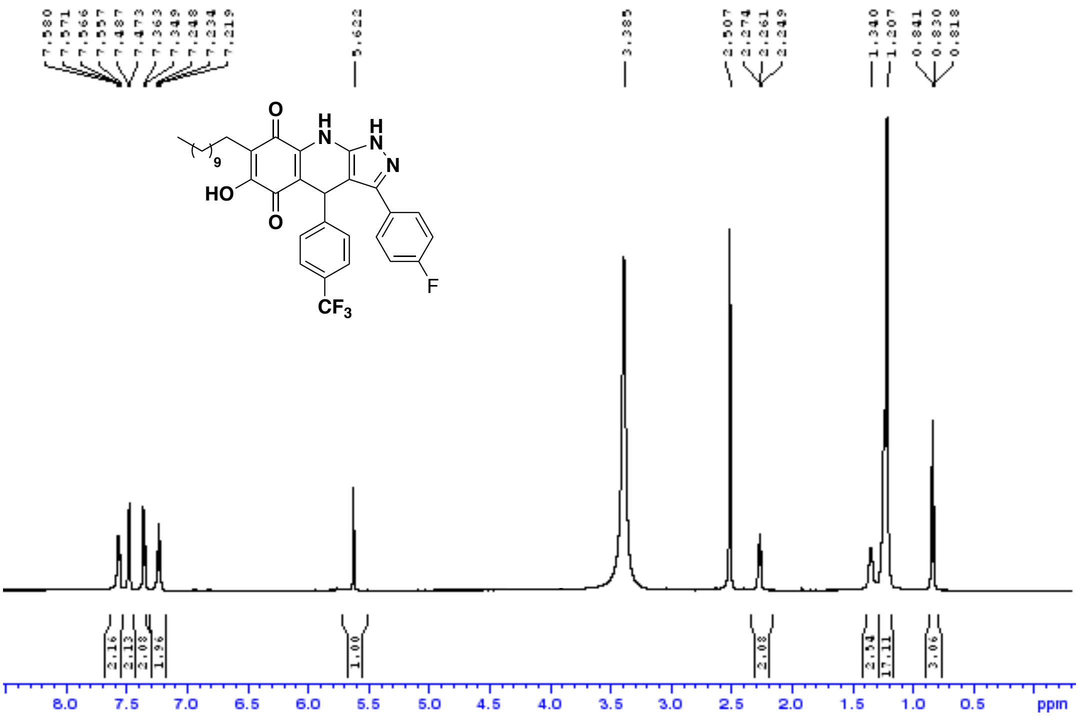

^{13}C-NMR (DMSO-d$_6$, 125 MHz) of compound 14c

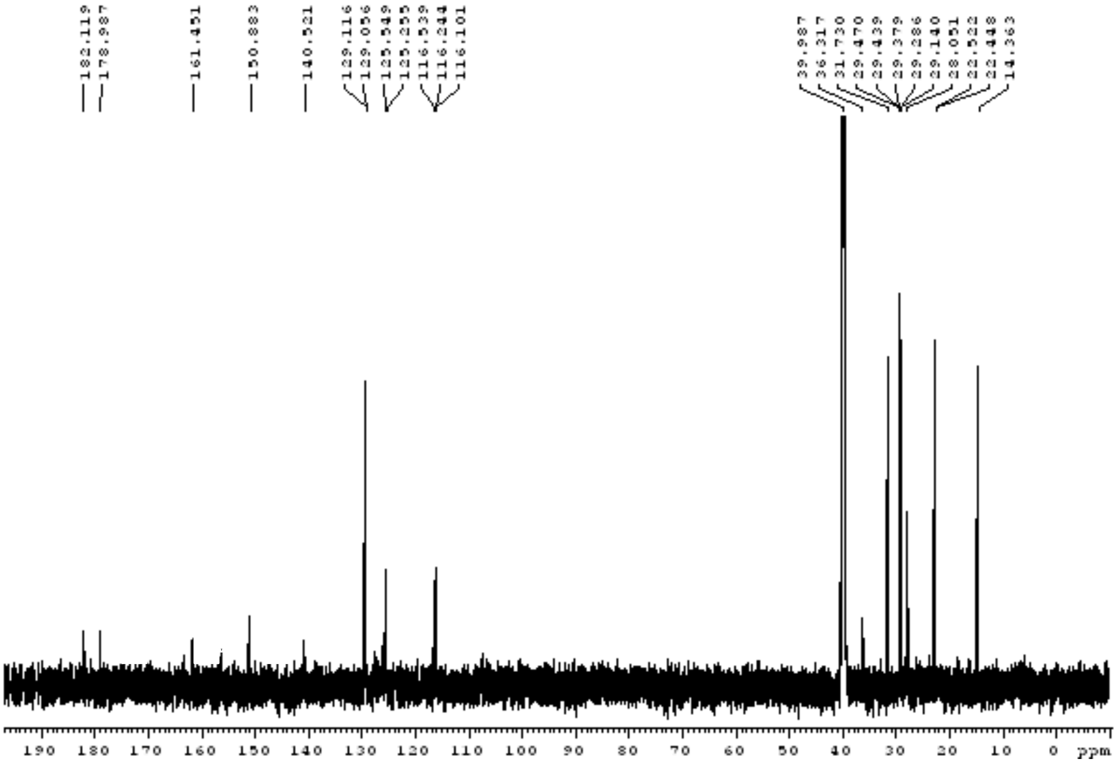

^{1}H-NMR (DMSO-d_6, 500 MHz) of compound 14d

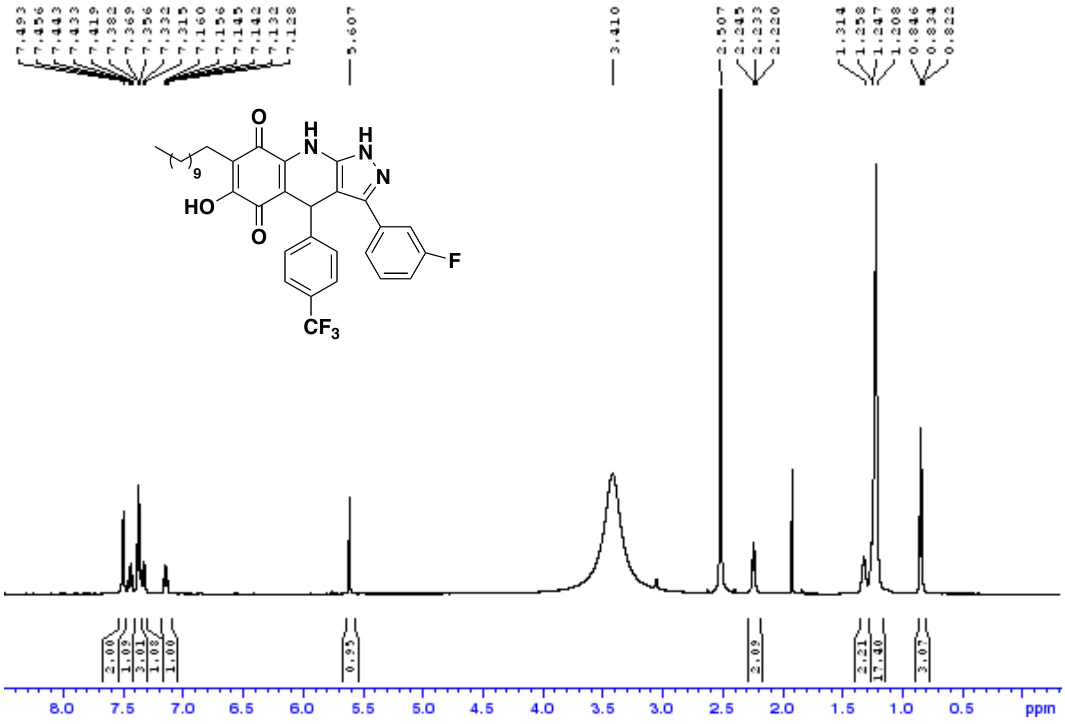

^{13}C-RMN (DMSO-d_6, 125 MHz) of compound 14d

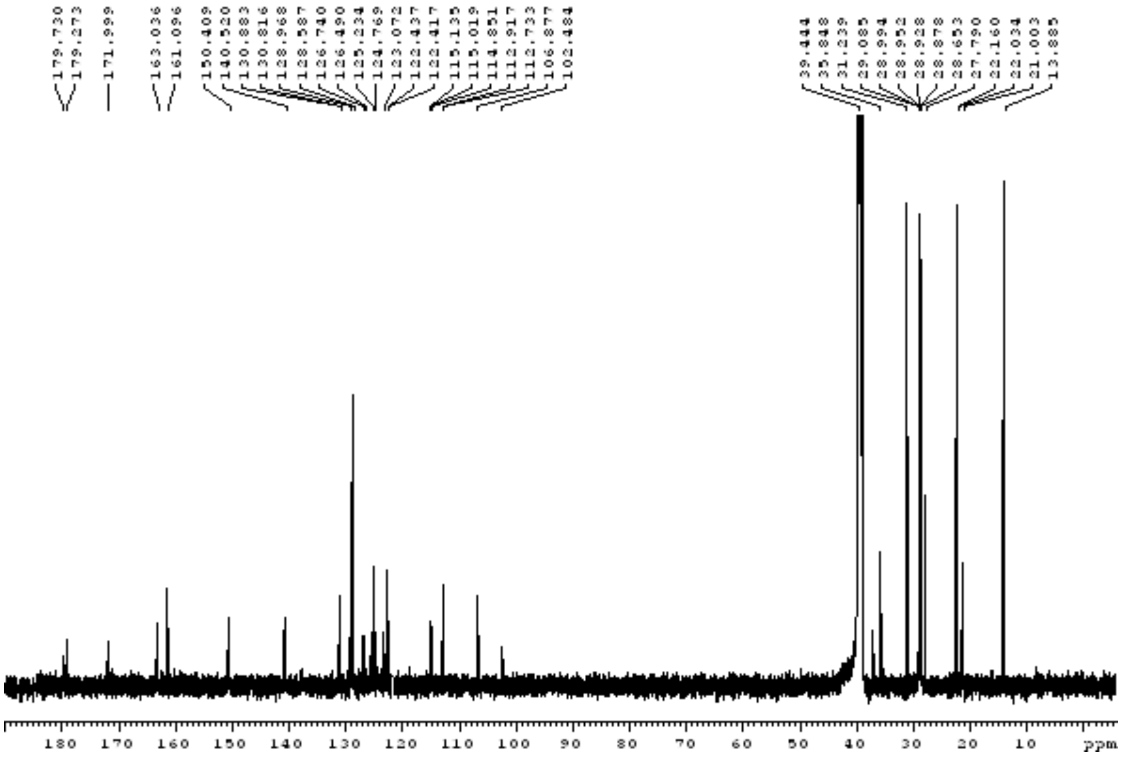

^{1}H-NMR (DMSO-d$_6$, 500 MHz) of compound 14e

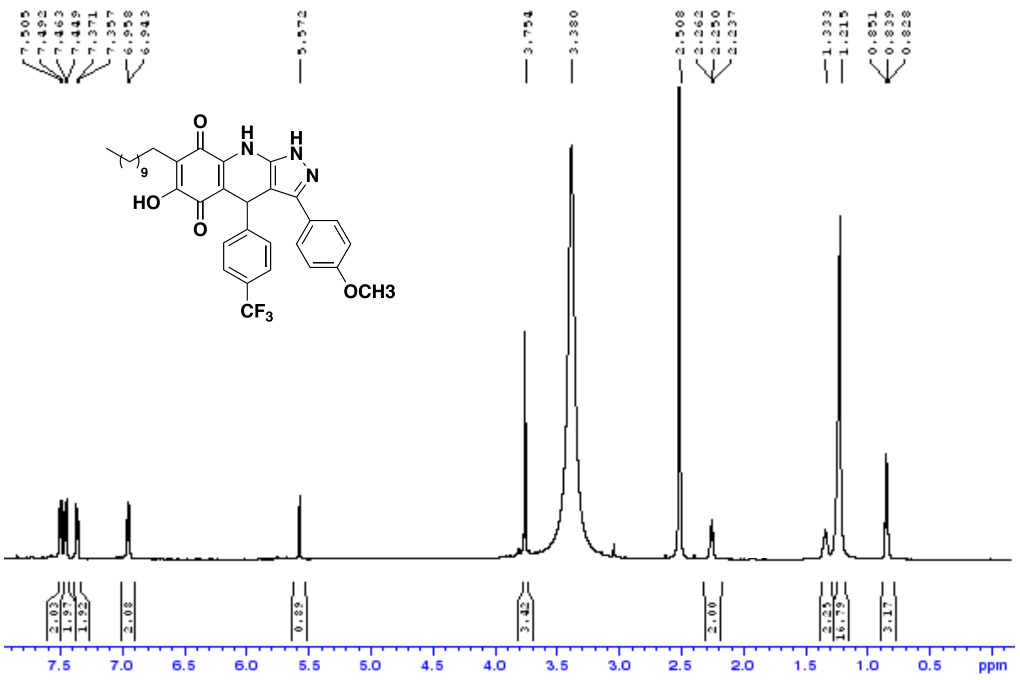

^{13}C-NMR (DMSO-d$_6$, 125 MHz) of compound 14e

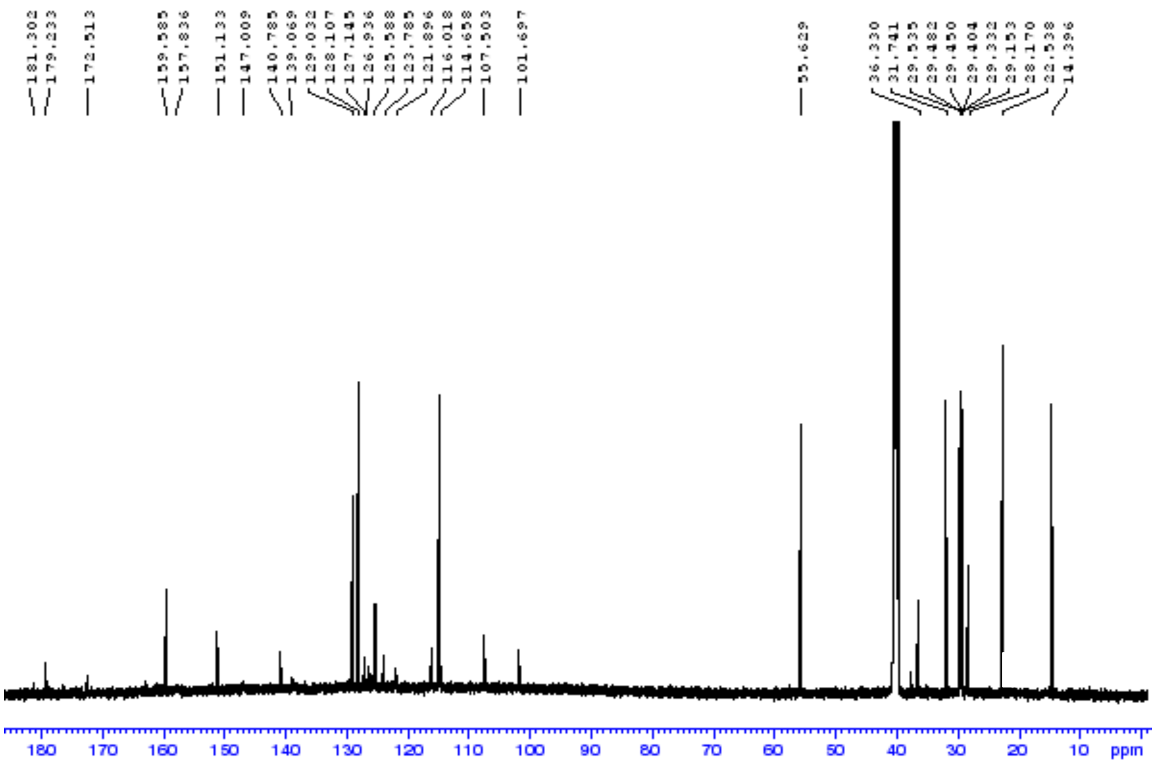

^{1}H-NMR (DMSO-d$_6$, 500 MHz) of compound 14f

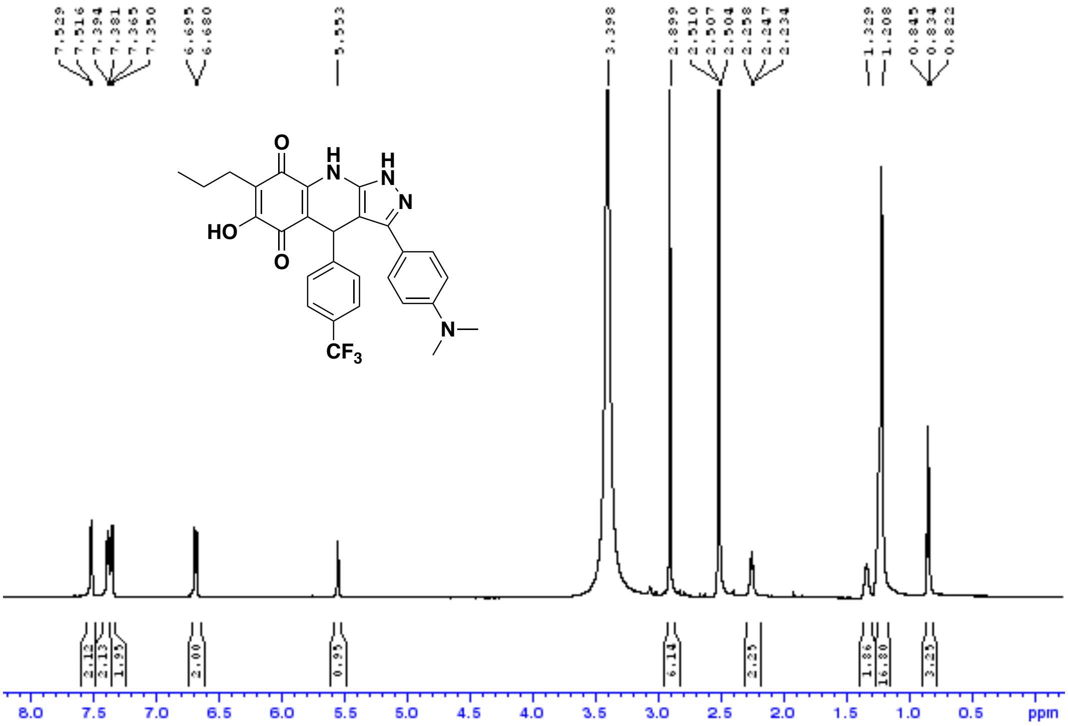

^{13}C-NMR DMSO-d$_6$, 125 MHz) of compound 14f

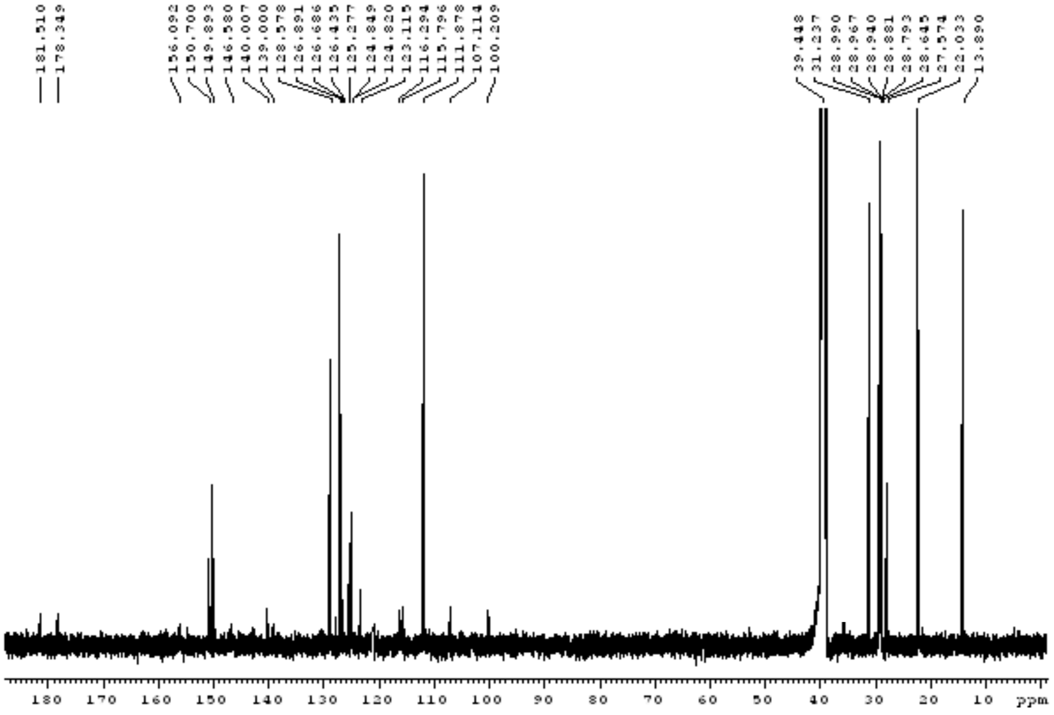

^{1}H-NMR (DMSO-d$_6$, 500 MHz) of compound 14g

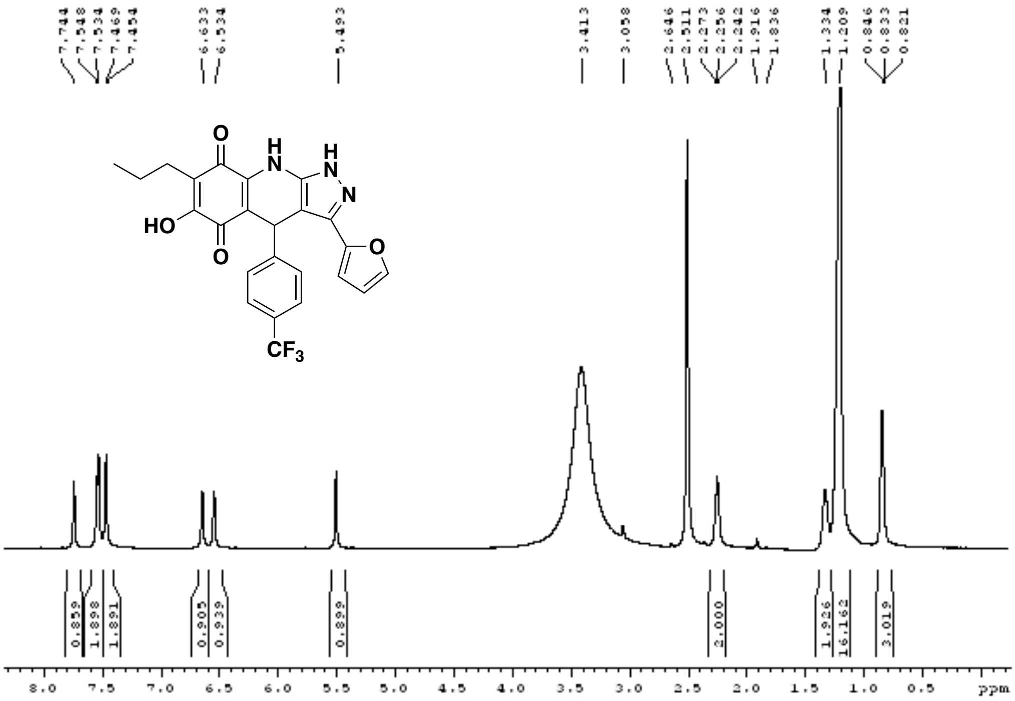

^{13}C-NMR (DMSO-d$_6$, 125 MHz) of compound 14g

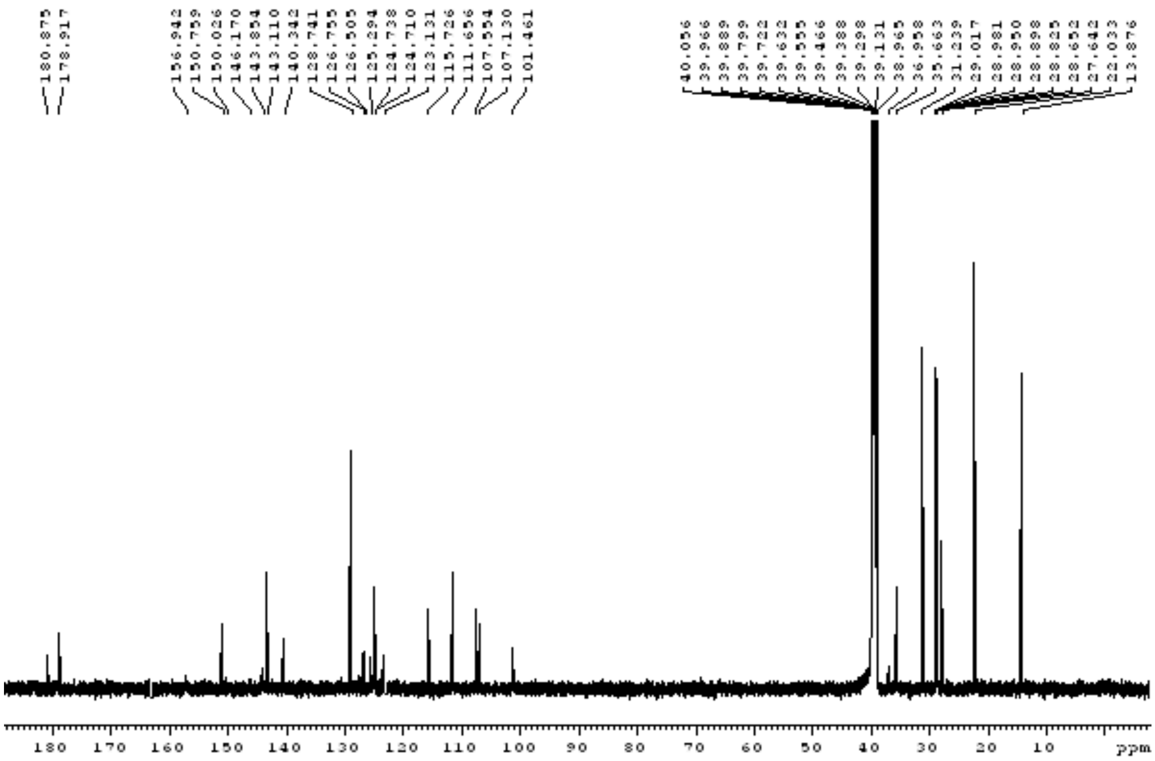

^{1}H-NMR (DMSO-d$_6$, 500 MHz) of compound 14h

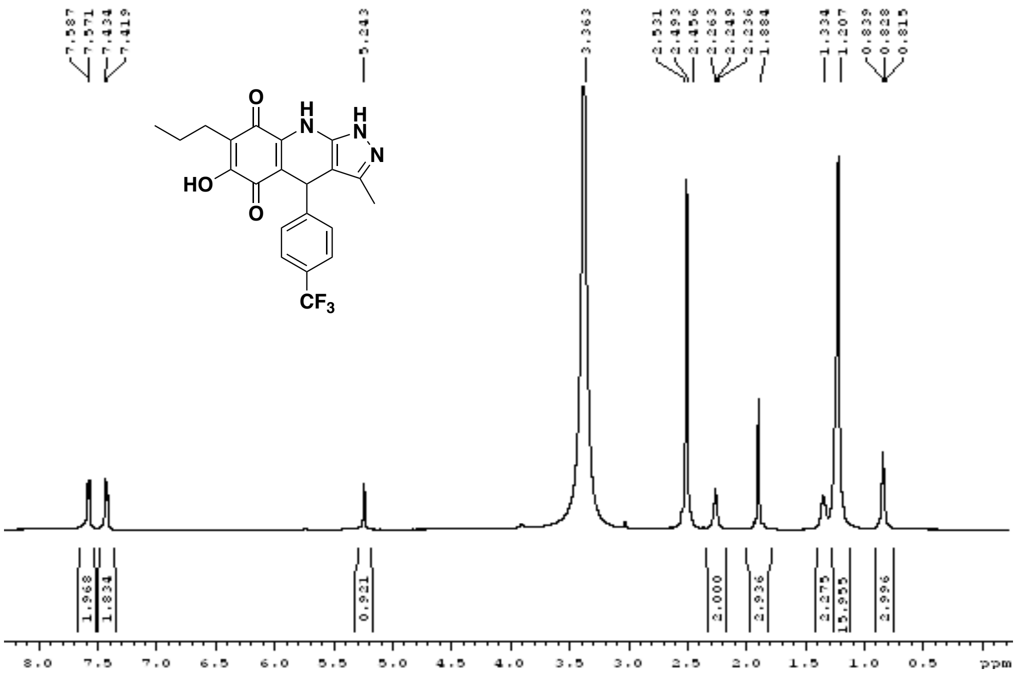

^{13}C-NMR (DMSO-d$_6$, 125 MHz) of compound 14h

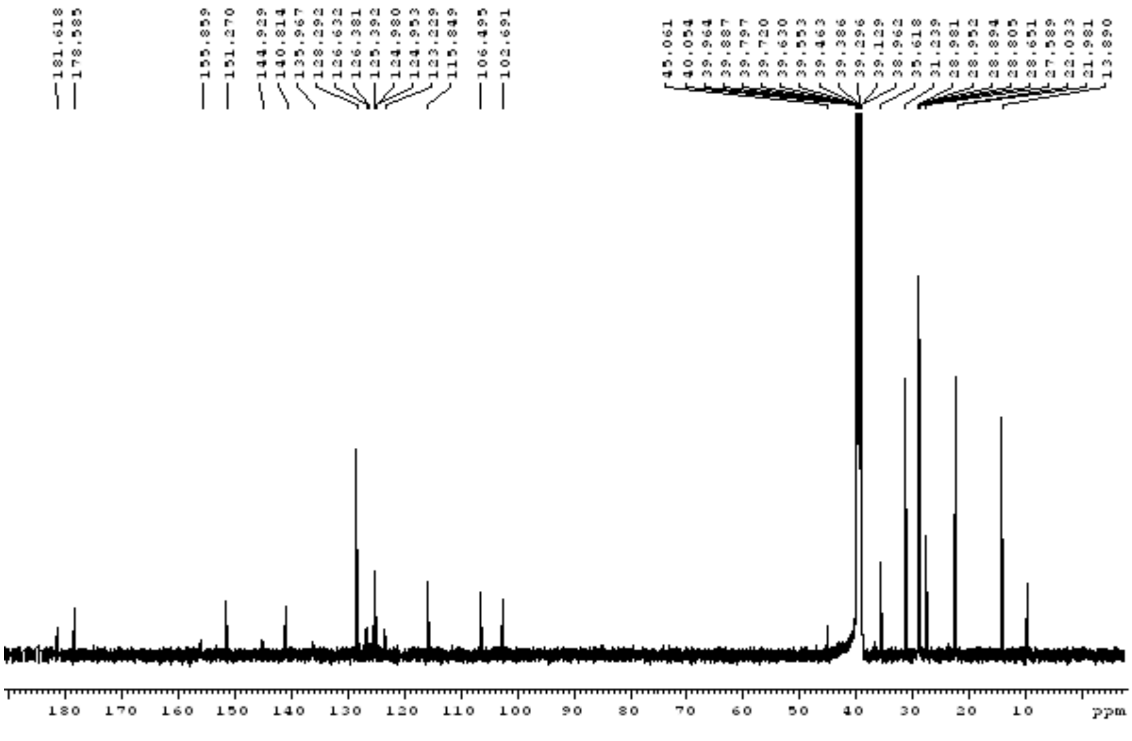

HSQC ($CDCl_3$, 500 MHz) of compound 4b

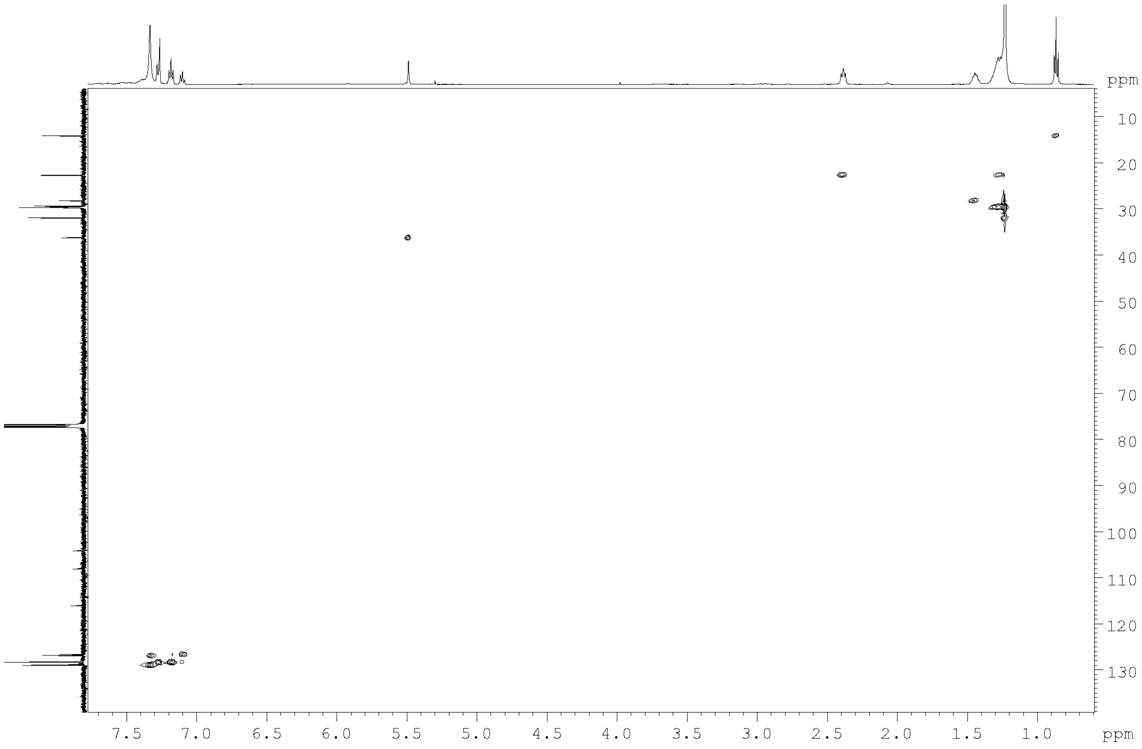

HMBC ($CDCl_3$, 500 MHz) of compound 4b

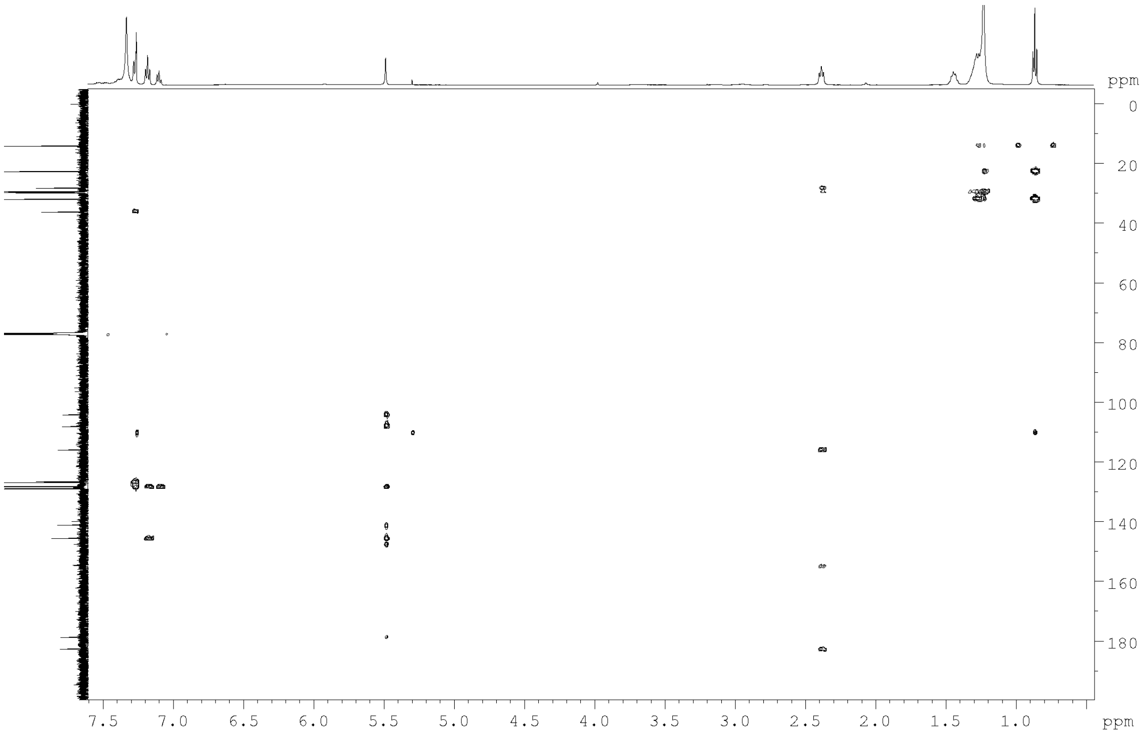

HSQC ($CDCl_3$, 500 MHz) of compound 4n

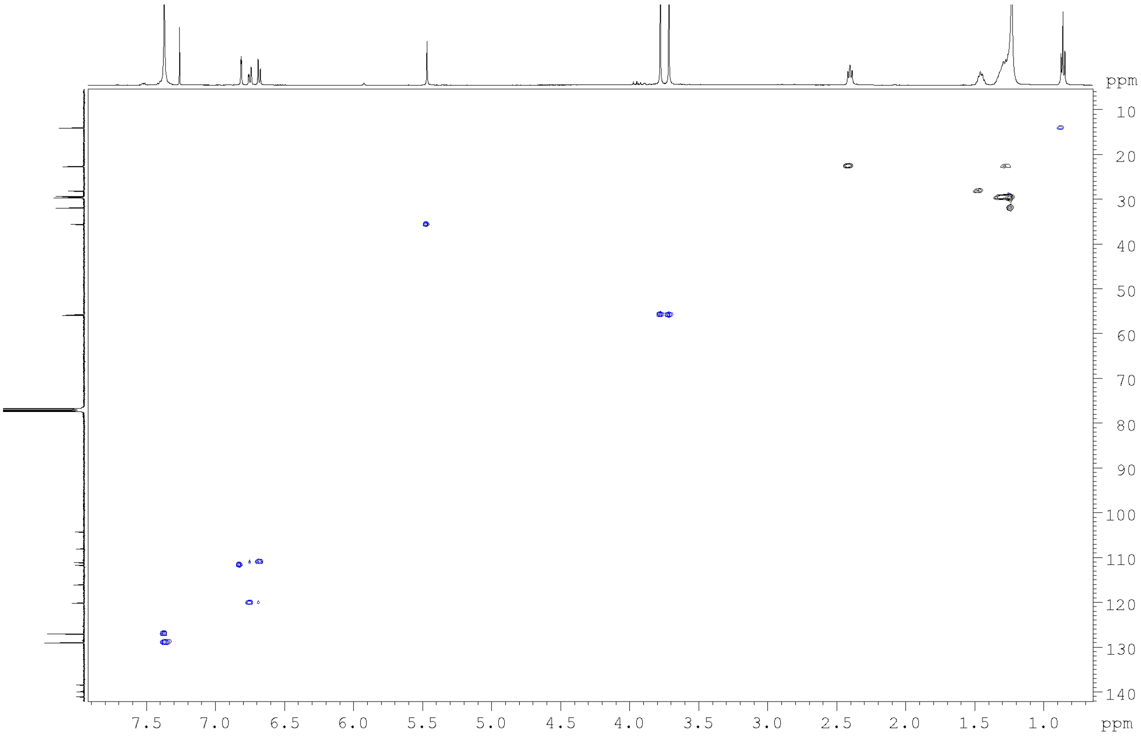

HMBC ($CDCl_3$, 500 MHz) of compound 4n

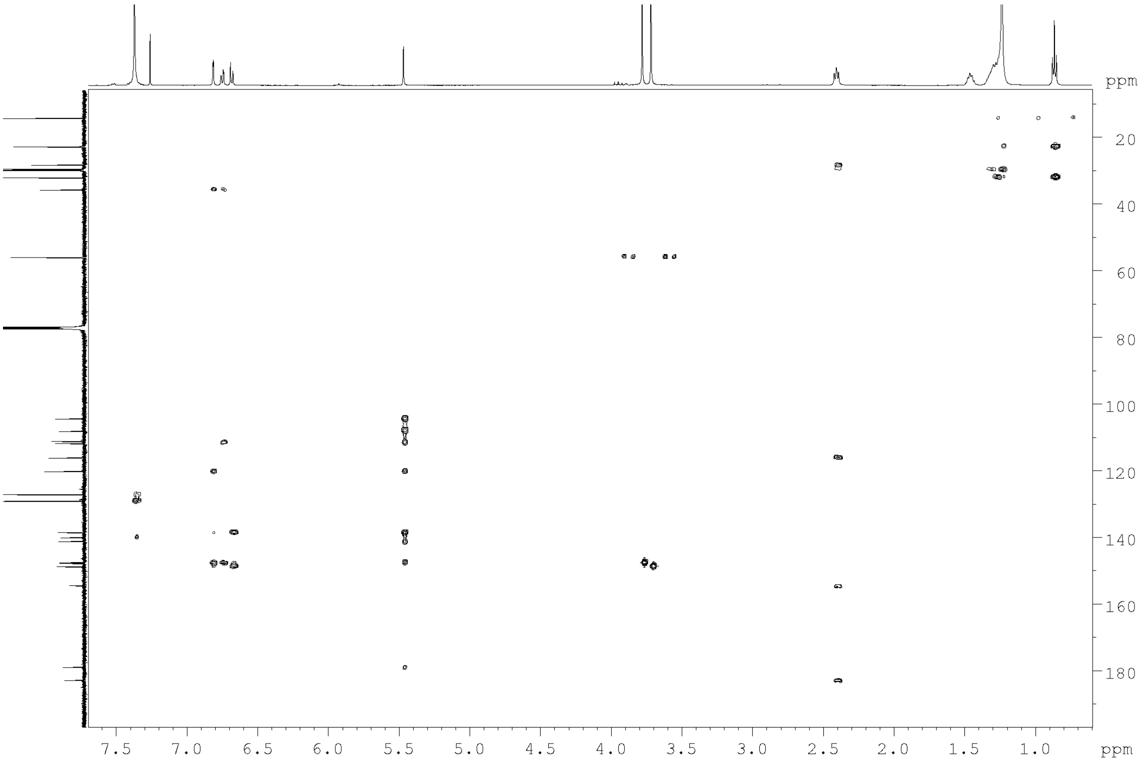

HMBC (DMSO-d$_6$, 500 MHz) of compound 4p

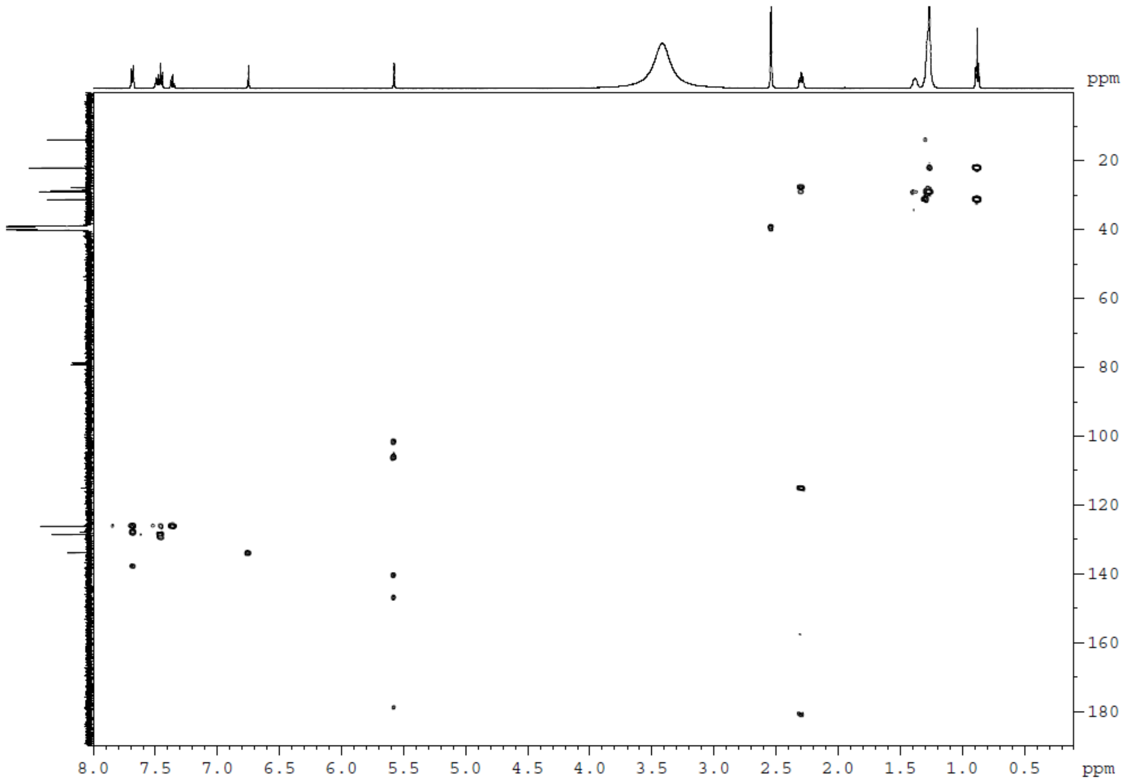

HMBC (CDCl$_3$, 500 MHz) of compound 4s

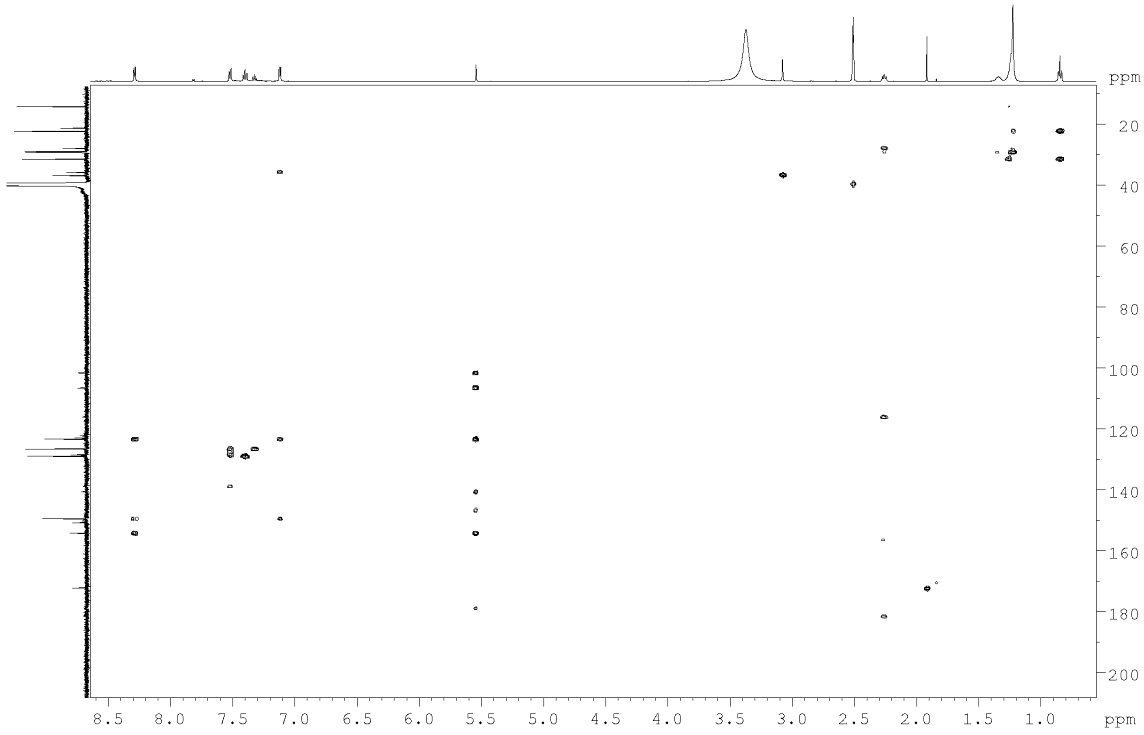

HMBC (DMSO-d$_6$, 500 MHz) of compound 4u

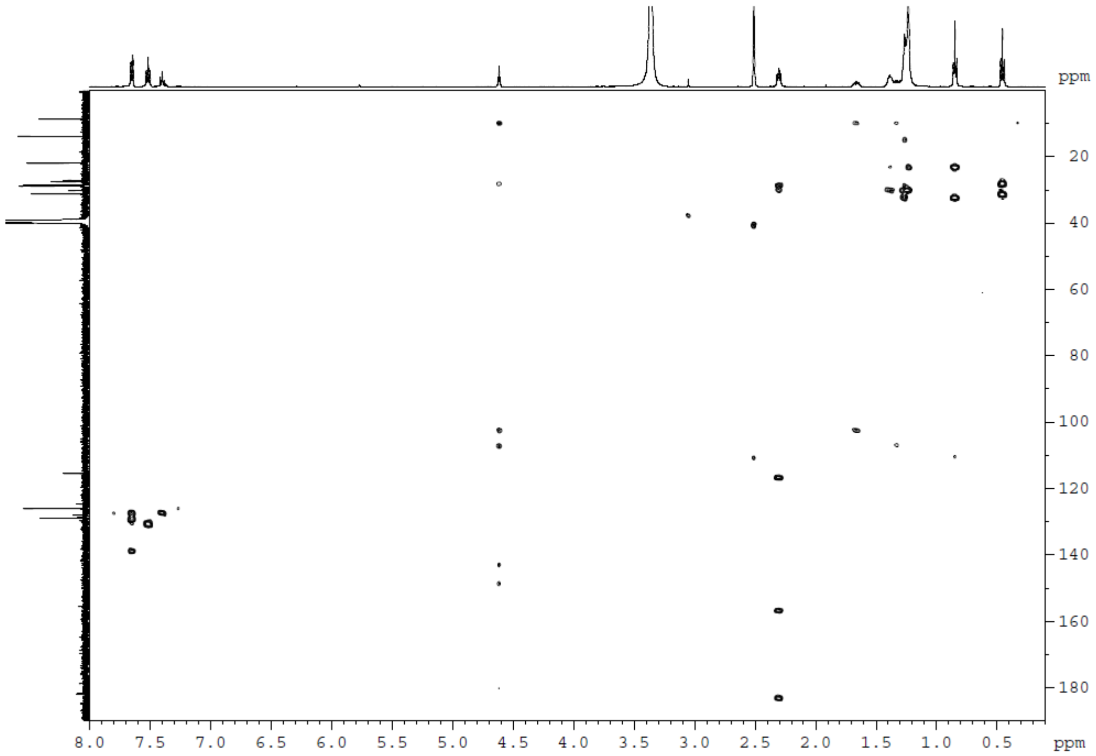

Supplement: Supplementary file 1 [file pharmaceuticals-14-01026-s001.zip › pharmaceuticals-1392251-supplementary.pdf]
